# Integrated Taxonomic Approaches to Gastrointestinal and Urinary Capillariid Nematodes from Wild and Domestic Mammals

**DOI:** 10.3390/pathogens14050455

**Published:** 2025-05-06

**Authors:** Masae Tamaru, Seiho Sakaguchi, Yasuhiro Souzu, Koichi Murata, Muchammad Yunus, Imron Rosyadi, Hiroshi Sato

**Affiliations:** 1 Laboratory of Parasitology, Joint Faculty of Veterinary Medicine, Yamaguchi University, 1677-1 Yoshida, Yamaguchi 753-8515, Japan; mtoda@city.ube.yamaguchi.jp (M.T.); a029tbu@yamaguchi-u.ac.jp (K.M.); 2 Department of Parasitology, Faculty of Veterinary Medicine, Airlangga University, Mulyorejo, Surabaya 60115, Indonesia; muhyunus_99@yahoo.com; 3 Joint Graduate School of Veterinary Medicine, Yamaguchi University, 1677-1 Yoshida, Yamaguchi 753-8515, Japan; imron.rosyadi@ugm.ac.id

**Keywords:** Capillariidae, taxonomy, SSU rDNA, morphology, *Aonchotheca*, *Calodium*, *Echinocoleus*, *Eucoleus*, *Liniscus*, *Pearsonema*, *Trichosomoides crassicauda*

## Abstract

Fine nematodes of the family Capillariidae parasitize various organs and tissues in fish, amphibians, reptiles, avians, and mammals. Currently classified into more than 20 genera, these nematodes are primarily distinguished based on the caudal structures of male worms. Morphological and molecular analyses were conducted on 15 mammal-parasitic species belonging to the genera *Aonchotheca* (*A. putorii*, *A. suzukii* n. sp., *A. suis* n. comb. (syn. *Capillaria suis*), *A. riukiuensis*, and *A. bilobata*), *Pearsonema* (*P. neoplica* n. sp., *P. feliscati*, *P. iharai* n. sp., and *P. toriii* n. sp.), *Liniscus* (*L. himizu*), *Calodium* (*C. hepaticum*), *Echinocoleus* (*E. yokoyamae* n. sp.), and *Eucoleus* (*E. kaneshiroi* n. sp., *E. aerophilus*, and *Eucoleus* sp.), using specimens from various wild and domestic animals in Japan and brown rats in Indonesia. As demonstrated in this study, nearly complete SSU rDNA sequencing is a powerful tool for differentiating closely related species and clarifying the phylogenetic relationships among morphologically similar capillariid worms. Additionally, most capillariid worms detected in dogs and cats are suspected to be shared with their respective wildlife reservoir mammals. Therefore, molecular characterization, combined with the microscopic observation of these parasites in wildlife mammals, provides a robust framework for accurate species identification, reliable classification, and epidemiological assessment.

## 1. Introduction

The taxonomy and classification of the family Capillariidae Railliet, 1915 (Nematoda: Trichocephalida: Trichinelloidea) have long been debated [1,2,3,4,5,6,7,8,9,10,11,12]. The current family or historical genus “*Capillaria*” sensu lato has been divided into either 23 [6], 27 [11], or 20 genera [12], with more than 400 species. Moravec and Beveridge [13] introduced a new genus, *Lobocapillaria* Moravec et Beveridge, 2017, to the family, prompting a revision of the classification in line with advancements in research on the fine and delicate nematodes of the family.

Previous studies [14,15,16] have morphologically and molecularly examined nine avian species of the family Capillariidae, including members of the genera *Eucoleus* Dujardin, 1845 (two species), *Aonchotheca* López-Neyra, 1947 (one species), *Capillaria* Zeder, 1800 (five species), and *Baruscapillaria* Moravec, 1982 (one species) based on the small subunit ribosomal RNA gene (SSU rDNA). Additional genetic data on the genera *Baruscapillaria*, *Calodium* Dujardin, 1945, *Capillaria*, *Eucoleus*, and *Pseudocapillaria* Freitas, 1959 have been published from avians and other categories of animals [17,18,19,20,21,22,23,24,25,26] using various molecular markers, such as the mitochondrial cytochrome *c* oxidase subunit 1 gene (*cox1*) for species such as *Eucolerus aerophilus* (Creplin, 1839) Dujardin, 1845, *Eucoleus boehmi* (Supperer, 1953) Moravec, 1982, and *Paracapillaria* spp. [20,25,27,28,29,30,31,32,33]. Phylogenetic analyses of SSU rDNA nucleotide sequences of capillariid worms have provisionally validated the latest classification of the family after Moravec’s generic redefinition in 1982 [5], which was primarily based on the morphology of the male caudal end [5,6,11,13].

Owing to their thin and fragile bodies, capillariid worms are difficult to identify based solely on morphological characteristics in laboratory settings. Furthermore, the identification of capillariid worms detected in field surveys and companion animal clinics often relies on capillariid eggs, which exhibit few morphological characteristics aside from their bioperculated barrel or lemon shapes and several texture patterns of the eggshell surface [27,30,31,34,35,36,37,38,39,40,41,42,43,44,45,46,47,48,49]. Therefore, modern taxonomic approaches integrating morphological and molecular analyses are essential to facilitate reliable specific identification of capillariid nematodes in field and clinical settings. Over the past decade, nuclear SSU rDNA or mitochondrial *cox1* nucleotide sequences have been increasingly collected and stored in GenBank at the National Center for Biotechnology Information (NCBI). This study presents the morphological and molecular traits of 15 mammalian capillariid species from the genera *Aonchotheca* (five species), *Pearsonema* Freitas et Mendonça, 1960 (four species), *Liniscus* Dujardin, 1845 (one species), *Calodium* (one species), *Echinocoleus* López-Neyra, 1947 (one species), and *Eucoleus* (three species). In addition, the SSU rDNA nucleotide sequence of *Trichosomoides crassicauda* (Bellingham, 1840) Railliet, 1895, a rat urinary parasite of the family Trichosomoididae Hall, 1916, was obtained. This family belongs to the superfamily Trichinelloidea Ward 1907, alongside the families Capillariidae, Trichuridae Ransom, 1911, and Trichinellidae Stiles et Crane, 1910

## 2. Materials and Methods

### 2.1. Animals Examined

Most capillariid worms analyzed in this study were collected between 1997 and 2020 from various wild mammals belonging to the families Canidae, Felidae, Mustelidae, Suidae, and Erinaceidae in Japan as follows: 153 raccoon dogs (*Nyctereutes viverrinus viverrinus* (Temmink, 1838)), 2011 feral alien raccoons (*Procyon lotor* (Linnaeus, 1758)), one feral domestic cat (*Felis silvestris catus* Linnaeus, 1758), 37 Japanese badgers (*Meles anakuma* Temminck, 1844), 101 Japanese martens (*Martes melampus melampus* (Wagner, 1841)), 14 alien Siberian weasels (*Mustela sibirica* (Pallas, 1773)), 8 Japanese weasels (*Mustela itatsi* (Temminck, 1884)), 76 feral alien American minks (*Neogale vison* (Schreber, 1777)) (syn. *Neovison vison* (Schreber, 1777)), 157 wild boars (*Sus scrofa leucomystax* (Temminck, 1842)), and 31 Amur hedgehogs (*Erinaceus amurensis* (Schrenk, 1858)), as shown in Supplementary Table S1. In addition, the urinary bladders were collected from 32 raccoon dogs, 477 feral alien raccoons, and 29 Japanese badgers from the southern part of the Wakayama Prefecture between February 2015 and September 2017. The gastrointestinal tract of a domestic goat (*Capra hircus* Linnaeus, 1758), capillariid eggs in urine samples from two domestic cats from different animal clinics, a dead Japanese weasel collected in Yamaguchi, and a dead Japanese shrew mole (*Urotrichus talpoides* Temminck, 1841) were also analyzed. The seasonal hunting of native mammals and year-round trapping of feral alien mammals were conducted in accordance with the general regulations and municipal annual plans issued by each prefecture in Japan. Frozen viscera (large mammals) or carcasses (small mammals) were provided for pathogen screening [50,51,52,53,54,55,56,57,58].

### 2.2. Parasitological Examination

Frozen viscera or carcasses were transported to the parasitology laboratory, and helminth parasites were collected according to standard procedures, as previously described [14,15,50,51,52,53,54,55]. Briefly, after a macroscopic inspection of all organs, digestive and respiratory tracts were opened longitudinally and immersed in tap water. Other luminal organs, such as the urinary bladders and gallbladders, were similarly processed. After the materials were left for a few hours, the mucosa was carefully scraped. Then, these materials were washed repeatedly via simple sedimentation in tap water. Sediments were checked thoroughly using a dissection microscope, and the collected parasites were grouped according to host organs, parasite sex, and certain morphological characteristics, and they were fixed in 10% neutral-buffered formalin solution or 70% alcohol. A conventional light microscope (Olympus BX60: Olympus Co., Shinjuku, Tokyo, Japan) was used for morphological observations of fixed specimens in 10% neutral-buffered formalin or 70% alcohol. Figures were drawn using a camera lucida. Measurements were performed on the figures using a digital curvimeter type S (Uchida Yoko, Tokyo, Japan) when necessary. Measurements are expressed in millimeters (mm) as ranges with mean values in parentheses. The collected specimens were deposited at the National Museum of Nature and Science, Tokyo, Japan (NSMT-As4071–As4292 and As4504-As4574).

An intensive morphological analysis of formalin-fixed specimens was performed using scanning electron microscopy (SEM). Specimens were washed three times in 0.2 M of Na_2_HPO_4_-NaH_2_PO_4_-buffered solution (PB), pH 7.8, and then immersed in 2.5% glutaraldehyde in PB overnight. The subsequent processing was similar to that described previously [15,55].

### 2.3. DNA Extraction, Polymerase Chain Reaction, and Sequencing

DNA of male and female worms of different isolates preserved in 70% alcohol was extracted using an Illustra™ tissue and cells genomicPrep Mini Spin Kit (GE Healthcare UK, Buckinghamshire, UK), according to the manufacturer’s instructions. Polymerase chain reaction (PCR) amplification of the SSU rDNA was performed as previously described [14]. When direct sequencing was unsatisfactory, the purified PCR products were cloned into a plasmid vector, pTA2 (Target Clone™; TOYOBO, Kita-ku, Osaka, Japan), and transformed into *Escherichia coli* JM109 (TOYOBO) according to the manufacturer’s instructions. After propagation, the plasmid DNA was extracted using a FastGene™ Plasmid Mini Kit (NIPPON Genetics Co., Tokyo, Japan), and inserts from at least three independent clones were sequenced using universal M13 forward and reverse primers. The nucleotide sequences reported in the present study are available in the DDBJ/EMBL/GenBank databases (accession nos. LC052349–LO052390, LC425007, LC425008, LC850894–LC850897, and LC858133–LC858139).

### 2.4. Phylogenetic Analysis

For phylogenetic analysis, the newly obtained SSU rDNA sequences of collected male and female worms and those of related species classified in the Capillariidae retrieved from the DDBJ/EMBL/GenBank databases were aligned using the CLUSTALW multiple alignment program [59] in MEGA7 [60] with subsequent manual adjustment. Regions that were poorly aligned and characters with a gap in any sequences were excluded from analyses; 1612 characters, of which 550 were variable, remained for the analysis of 58 sequences (Capillariidae, Trichuridae, and Trichosomoididae). Maximum likelihood (ML) analysis was performed using the program PhyML [61,62] provided on the “phylogeny.fr” website (https://www.phylogeny.fr/). The probability of the inferred branches was assessed using the approximate likelihood ratio test (aLRT), which is an alternative to the non-parametric bootstrap estimation of branch support [63]. The SSU rDNA sequence of *T. crassicauda* (LC425007) was used as the outgroup to construct the ML phylogenetic tree. Accession numbers of the analyzed sequences are shown in the phylogenetic tree. To analyze the fine phylogenetic relationships of members of the genera *Aonchotheca*, *Calodium*, *Liniscus*, and *Pearsonema*, together with those of the genera *Baruscapillaria* and *Pseudocapillaria*, 40 sequences were selected, and regions that were poorly aligned and characters with a gap in any sequences were excluded from analyses; 1701 characters, of which 201 were variable, remained. The accession numbers of the analyzed sequences are shown in the phylogenetic tree.

### 2.5. Putative Secondary Structure of the SSU rRNA

A putative secondary structure of the SSU rRNA of *A. putorii* (DDBJ/EMBL/GenBank accession no. LC052349) was constructed with reference to the proposed secondary structure of the SSU rRNA of *Homo sapiens* (K03432) [64]. After the basic secondary structure framework of the SSU rRNA molecule of *A. putorii* was predicted, the secondary structures of the non-conserved parts of the molecule were predicted using the Mfold web server (http://www.unafold.org/mfold/applications/rna-folding-form.php accessed on 24 December 2024), where RNA folding structures were proposed based on an energy minimization approach [65].

## 3. Results

Male and female nematodes of the family Capillariidae were collected from the respiratory tract, alimentary tract, and urinary bladder of the examined mammals. The collected nematodes were divided into 15 species belonging to the genera *Aonchotheca* (five species), *Echinocoleus* (one species), *Eucoleus* (three species), *Calodium* (one species), *Pearsonema* (four species), and *Liniscus* (one species), as detailed below based on the morphology of the male caudal end, female vulval position, vulval appendages, parasite location, and other morphometric features. Hepatic capillariasis was evident to the naked eye in the livers of brown rats, with granulomatous white patches containing numerous deposited capillariid eggs; however, isolating adult worms was challenging. *Calodium hepaticum* (Bancroft, 1893) Moravec, 1982, was identified based on the morphological characteristics of deposited eggs. Adult worms of *T. crassicauda* of the family Trichosomoididae (Nematoda: Trichocephalida: Trichinelloidea) were also collected from the urinary bladders of brown rats in Surabaya, Indonesia.

### 3.1. Aonchotheca putorii (Rudolphi, 1819) López-Neyra, 1947

#### 3.1.1. Worm Recovery and Morphological Observation

This species was found primarily in the stomach though occasionally in the upper small intestine of various mammals across more than 10 prefectures in Japan. These hosts included Japanese raccoon dogs, feral alien raccoons, Japanese badgers, Japanese martens, alien Siberian weasels, feral alien American minks, feral domestic cats, and feral alien Amur hedgehogs (Supplementary Table S1). Among 22 feral alien hedgehogs collected from four localities in Shizuoka Prefecture, 18 animals (prevalence 81.8%) were infected, with a notably high intensity. More than half of the examined hedgehogs (11 individuals) harbored over 100 worms, with the highest recorded recovery reaching 1641 worms (geometic mean: 111.4 [*n* = 11]). In contrast, nine feral alien hedgehogs from two other localities in the same prefecture showed no parasitic infection. In other mammalian hosts, the intensity was relatively low, with most cases yielding only 1–9 worms per individual.

**Figure 1:**
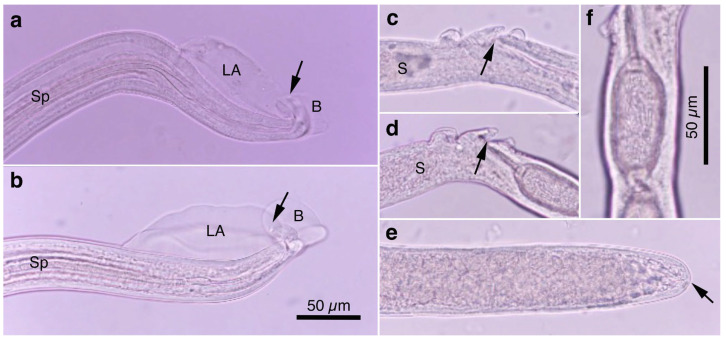
Morphology of *Aonchotheca putorii*. (**a**) Caudal end of morphotype “Type A” male worm with lateral alae (LA) and membranous bursa (B) supported with elongated digitiform projections (arrow). Spicule (Sp). (**b**) Caudal end of morphotype “Type B” male worm with LA and membranous B supported with stumpy digitiform projections (arrow). (**c**,**d**) Vulval appendages of female worms at three regions (anterior and posterior swellings of the vulva, and a vulval plate from the anterior edge of the vulval opening (arrows). End of stichosome (S). (**e**) Posterior end of a female worm with a terminal anus (arrow). (**f**) Intrauterine egg with striated eggshell surface texture. Photographs (**a**–**e**) at the same magnification and a scale bar is shown in (**b**).

**Figure 2:**
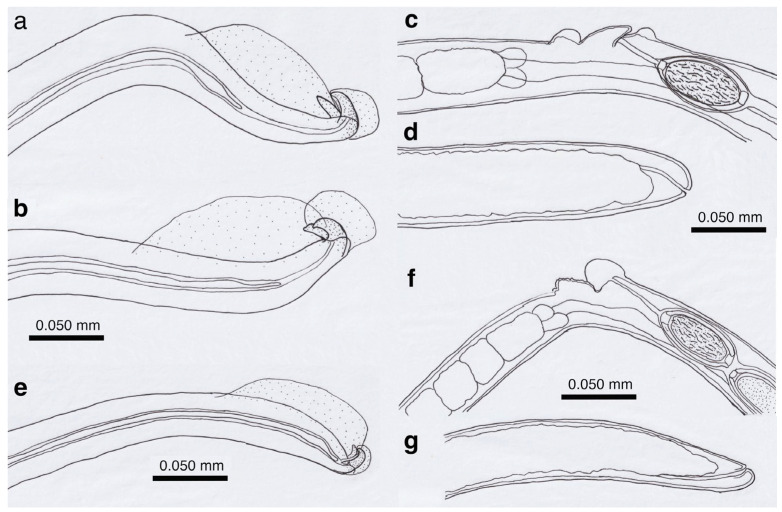
Line drawings of *Aonchotheca putorii* (**a**–**d**) and *A. suzukii* n. sp. (**e**–**g**). (**a**) Caudal end of morphotype “Type A” male worm. (**b**) Caudal end of morphotype “Type B” male worm. (**c**) Vulval appendages of a female worm at three regions (anterior and posterior swellings of the vulva, and a vulval plate from the anterior edge of the vulval opening), and intrauterine egg with striated eggshell surface texture. (**d**) Posterior end of a female worm with a terminal anus. (**e**) Caudal end of *A. suzukii* n. sp. male worm. (**f**) Vulval appendages of *A. suzukii* n. sp. female worm at two regions (small subcuticular elevation anterior to the vulval opening and cuticular ballooned swelling around it), and an intrauterine egg with striated eggshell surface texture. (**g**) Posterior end of *A. suzukii* n. sp. female worm with a subterminal anus.

Pronounced dimorphism was observed in two morphological features of male worms. Distinctions were evident in spicule lengths (0.30–0.48 mm [*n* = 44] vs. 0.20–0.25 mm [*n* = 14]) and in the morphology of ventrally bent lateral projections supporting the membranous bursa, which appeared either slender and elongated or thick and short (Figure 1a,b, Figure 2a,b, Table 1 and Supplementary Table S2). Male worms with longer spicules and narrower lateral projections at the caudal end were designated as Type A, whereas the others were designated Type B in the present study.

Typically, Type A male worms were found in feral alien Amur hedgehogs from Shizuoka, whereas Type B male worms were identified in feral alien American minks from Fukushima. However, in other animals, male worms of both types were found regardless of their geographical origins, and, in some cases, both types coexisted within the same host individual. Male worms of either type exhibited developed caudal lateral alae and membranous bursa supported with ventrally bent lateral projections, which were elongated in Type A and thick in Type B worms. The spicule was thin and sclerotized, with a lancet-shaped tip, and was longer in Type A (0.301–0.482 (0.363) mm) than in Type B (0.203–0.247 (0.216) mm). The spicular sheath surface was smooth but transversely striated. Female worms had a vulva located at approximately two-fifths of the body length from the anterior end, along with distinct vulval appendages in three regions—namely anterior and posterior swellings around the vulva and a vulval plate extending from the anterior edge of the vulval opening (Figure 1c,d and Figure 2c)—or at two areas. The posterior end of female worms was rounded with an almost terminal anus (Figure 1e and Figure 2d). Bioperculated barrel-shaped eggs were somewhat skewed with irregular longitudinal striae, often fused to form a reticulate pattern on the eggshell surface (Figure 1f and Figure 2c).

#### 3.1.2. Molecular Characterization

*Aonchotheca putorii* specimens, including male morphotypes A and B, were subjected to SSU rDNA sequencing, revealing four major genotypes (Ia/Ib, II, III, and IV), as shown in Table 2. Nucleotide substitutions were concentrated in specific hairpin loops, rather than being evenly distributed throughout the gene (Supplementary Figure S1). Nucleotide changes, including insertions or deletions (indels), were evident among the different genotypes, particularly at the tip of one hairpin loop between the 210th and 240th nucleotide positions of Genotype Ia (DDBJ/EMBL/GenBank accession no. LC052349). Nucleotide identities of comparable areas of the SSU rDNA gene between the major genotypes (I, II, III, and IV) ranged from 98.87% (1571/1589) to 99.79% (1805/1809), as shown in Table 3.

#### 3.1.3. Remarks

This species serves as the type species of the genus *Aonchotheca* [5], which parasitizes the stomach and small intestine of various mustelid hosts (*Mustela* spp. including *M. putorius*, *Neogale vison*, *Lutra lutra*, *Lutreola* spp., *Martes* spp., and *Meles* spp.), skunks, raccoons, foxes, wolves, domestic cats, dogs, and European hedgehogs (*Erinaceus europaeus* Linnaeus, 1758) [2,3,4,6,66,67,68,69,70]. Crum et al. [71] reported a high prevalence (38/53) of *A. putorii* in the stomach of North American black bears (*Ursus americanus* Pallas, 1789). In Japan, this species has been recorded in weasels, martens, raccoon dogs, feral alien raccoons, domestic dogs, and cats [50,51,72,73]; however, details regarding the morphological dimorphism of male worms have not been documented.

Male worms exhibited minor but apparent dimorphisms in spicule length and terminal lateral projections that supported the caudal membranous bursa, whereas female worms did not display clear polymorphisms in any morphological features. Although variations in vulval appendages among individual female worms were observed, these variations did not provide a basis for dividing collected female specimens into morphological groups. Dorney and Lauerman [74] previously documented morphological dimorphism in male *A. putorii* (syn. *Capillaria mustelorum* Cameron et Parnell, 1933; *C. erinacei* (Rudolphi, 1819) Travassos, 1915) from American minks in Wisconsin, USA, with differences in caudal lateral alae length (103–139 µm vs. 80–96 µm) and lateral projections supporting the caudal bursa (19–39 µm vs. 6–10 µm), whereas no such dimorphism was observed in female worms. This dimorphism was present in male worms isolated from the stomach and small intestine. Similarly, Butterworth and Beverley-Burton [4] observed the same dimorphism in male *A. putorii* collected from several mustelid hosts, striped skunks, hedgehogs, and common raccoons in Ontario, Canada. Host and tissue preferences for different morphotypes were also noted with one morphotype characterized by shorter caudal lateral alae and terminal lateral projections supporting the caudal bursa, preferentially parasitizing the stomach, rather than the small intestine of raccoons, and another morphotype in the small intestine, rather than the stomach, of American minks [4]. However, the male caudal dimorphism observed in the present and previous studies [4,74] is not identical, suggesting the existence of multiple morphotypes within currently recognized *A. putorii* specimens. Molecular markers, specifically the SSU rDNA gene, partially support the morphological division of *A. putorii*; however, the morphological traits of the different genetic lineages of this species are unknown. Further research is required to clarify the significance of multiple genotypes (genetic lineages) of *A. putorii*, including the possibility of reclassifying species or subspecies. The selection of appropriate SSU rDNA regions for genotyping of *A. putorii* or full-length sequencing of the gene is necessary, as indicated in Table 2. Kołodziej-Sobocińska et al. [75] characterized SSU rDNA sequences of *A. putorii* from feral alien American minks from Poland, sequencing fragments of the gene spanning from base positions 1037 to 1636 relative to the 5′-terminus of the sequence analyzed in this study (LC052349) using a primer pair of 18S 965 (5′-GGC GAT CAG ATA CCG CCC TAG TT-3′) and 18S 1573R (5′-TAC AAA GGG CAG GGA CGT AAT-3′), according to Powers et al. [76]. The sequencing region of the Polish study showed poor nucleotide variations among the different SSU rDNA genotypes identified in this study, rendering their molecular approach incapable of distinguishing the newly identified genotypes I, III, and IV (Table 2).

Transmission of *A. putorii* occurs through both direct and indirect routes, with earthworms serving as intermediate or paratenic hosts [6,77,78]. Given the high prevalence of this species in wildlife mammals [50,51,66,68,70,75,79], domestic cats and hunting dogs or stray dogs are susceptible to infection through the ingestion of contaminated foods containing embryonated eggs from soil or infected earthworms, some of which result in clinical gastritis [67,80,81].

### 3.2. Aonchotheca suzukii n. sp.

(Syn. *Capillaria* sp. sensu Uchida, Uchida, Murata et Udagawa, 1984; *Aonchotheca* (*Aonchotheca*) sp. Tamaru-2010)

#### 3.2.1. Worm Recovery and Morphological Observation

This species was detected in the stomach and small intestine of at least six raccoon dogs, 35 feral alien raccoons, six badgers, and one feral cat, as well as in the stomach of at least six wild boars. The animals were collected from seven prefectures in Japan. In wild Carnivora mammals, mixed infections with *A. putorii* were common, ranging from one-third to more than half of animals parasitized with gastrointestinal capillariid nematodes. However, in wild boars, the detection rate was comparatively lower: two (14.4%) of 14 animals caught in Hyogo Prefecture and four (16.0%) of 25 animals caught in Wakayama Prefecture. In contrast, *Aonchotheca suis* (Yamaguti, 1943) n. comb. was found in nine (62.3%) and 17 (68.0%) wild boars, and *Aonchotheca riukiuensis* (Shoho et Machida, 1979) Moravec, 1982 was found in 11 (78.6%) and 20 (80.0%) wild boars in these prefectures, respectively. Additionally, the infection intensity of this new *Aonchotheca* sp. in wild boars was lower than that of the other two *Aonchotheca* spp. in the same host animals.

**Figure 3:**
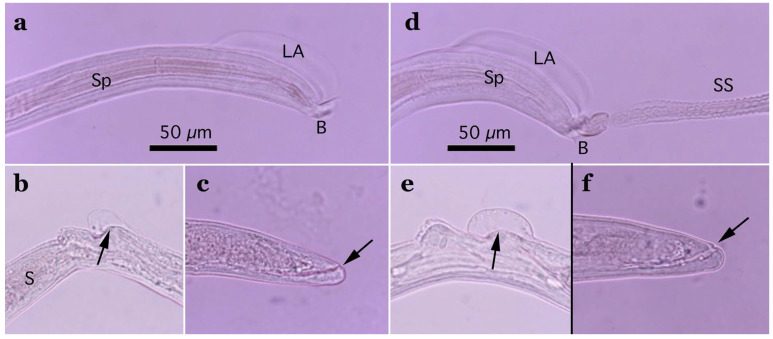
Morphology of *Aonchotheca suzukii* n. sp. ((**a**–**c**), specimens from feral alien raccoons, (**d**–**f**), specimens from wild boars). (**a**,**d**) Caudal end of male worm with lateral alae (LA) and membranous bursa (B) supported with short digitiform projections. Spicule (Sp), and extruded spicular sheath (SS) showing transverse striations. (**b**,**e**) Vulval appendages of female worm; small subcuticular elevation at the anterior to the vulval opening (arrow) and cuticular ballooned swelling around it. End of stichosome (S). (**c**,**f**) Posterior end of female worm with subterminal anus (arrow). Photographs (**a**–**f**) at the same magnification, and scale bar is shown in (**a**,**d**).

Morphologically, this species closely resembled *A. putorii* but differed in several key features. The male worm exhibited a gradual tapering of the caudal ends, in contrast to the abrupt tapering observed in *A. putorii*. The caudal bursa of male worms is poorly developed, with one small digitiform projection on two terminal lateral expansions. Female worms possessed vulval appendages, including a subcuticular elevation in the anterior portion of the vulva, a dome-shaped cuticular swelling around the vulval opening, and a subterminally opened anus (Figure 2e–g and Figure 3). *Aonchotheca suzukii* n. sp. has been described using specimens collected from feral alien raccoons. The measurements of male and females worms from different hosts are shown in Table 4.

##### *Aonchotheca suzukii* n. sp. (Nematoda: Trichocephalida: Capillariidae)

(Syn. *Capillaria* sp. sensu Uchida, Uchida, Murata et Udagawa, 1984)

Description: Thin filiform nematode with a rounded, narrowed head end and indistinct oral papillae. Worm body entirely covered with a transversely striated cuticle, and gradually tapered at both ends.

Male (*n* = 4): Body length of 5.78–6.66 (mean: 6.13) mm, maximum body width of 0.042–0.050 (0.045) mm around the middle of the body near the end of the esophagus (stichosome), situated 2.74–3.12 (2.93) mm from the head end. Ratio of the posterior part after the esophageal end to the anterior part almost equal at 1.05–1.13 (1.09). Posterior extremity of the body curved toward the back and gradually tapered with evident caudal lateral alae (Figure 2e and Figure 3a,d). Posterior end of the body with two lateral expansions and a membranous caudal bursa, supported with two ventrolaterally directed, relatively small digitiform projections bent anteriorly on each terminal expansion. Small papillae on the top of the ventrolateral projections. Filiform spicule, lightly to moderately sclerotized, 0.370–0.455 (0.410) mm long. Spicular sheath, non-spinous and transversely striated.

Female (*n* = 5): Female worms bigger than male worms, 6.82–9.23 (8.26) mm long, with a maximum body width of 0.049–0.057 (0.052) mm around the mid-uterine part. End of the stichosome at 2.44–3.29 (2.99) mm from the anterior end, and the posterior part after the esophageal end longer than the anterior part by a ratio of 1.67–1.86 (1.77). The vulva ventrally opened at the level of the esophageal end but posteriorly with a distance of 0.068–0.186 (0.135) mm. Small subcuticular elevation anterior to the vulval opening and cuticular ballooned swelling around it (Figure 2f and Figure 3b,e). Caudal extremity gradually tapered with subterminal anus (Figure 2g and Figure 3c,f). Bioperculated barrel-shaped eggs, 0.058–0.064 (0.060) mm by 0.023–0.029 (0.026) mm. Eggshell surfaces with longitudinal irregular striae, often fused to form a reticulate pattern (Figure 2f).

#### 3.2.2. Molecular Characterization

Nine SSU rDNA sequences of *A. suzukii* n. sp. (accession nos. LC052366–LC052374) were newly obtained using nine specimens of different origins in host species and locality, exhibiting a high identity with each other (≥99.89%; ≥1805/1807). When compared with *A. putorii* genotypes I to IV, the new species showed 97.68% (1765/1807)–98.06% (1772/ 1807) identity with five to 11 indels, indicating that *A. suzukii* n. sp. is distinct from *A. putorii* and other capillariid worms.

#### 3.2.3. Remarks

The morphology of this species closely resembles that of *A. putorii* except for some minor differences that could be misinterpreted as variations owing to different developing stages or juvenile characteristics of the nematodes. Uchida et al. [82] documented the presence of an *Aonchotheca* sp. distinct from *A. putorii* in the esophagus and stomach of raccoon dogs in Kanagawa Prefecture, Japan. Unique vulvar appendages and terminal ventrolateral projections were observed in both female and male specimens, distinguishing them from *A. putorii*. The caudal extremity of male worms of *A. putorii* dorsally curves, gradually tapers and then bends ventrally over the region of the caudal lateral alae. In contrast, the caudal extremity of *A. suzukii* n. sp. also curves dorsally but does not vend ventrally toward the terminus. These morphological features, illustrated as well by Uchida et al. [82], align with those recorded for *A. suzukii* n. sp. in the present study.

Molecular genetic analyses confirmed that *A. suzukii* n. sp. isolates, exhibiting identical morphology, were found in the stomach and small intestine of various wild Carnivora mammals and the stomachs of wild boars, demonstrating their conspecificity.

#### 3.2.4. Taxonomic Summary

Type host: *Procyon lotor* (Linnaeus, 1758) (Carnivora: Procyonidae), feral alien raccoons in Japan, introduced originally from North America.

Additional hosts: *Nyctereutes viverrinus viverrinus* (Temmink, 1838) (Carnivora: Canidae), *Meles anakuma* Temminck, 1842 (Carnivora: Mustelidae), *Felis silvestris catus* Linnaeus, 1758 (Carnivora: Felidae), *Sus scrofa leucomystax* Temminck, 1842 (Artiodactyla: Suidae)

Site of infection: stomach, and small intestine.

Type locality: Nagasaki Prefecture (Sasebo), Japan.

Additional localities: Nagasaki Prefecture (Hasami), Saga Prefecture (Takeo, Imari, Karatsu, Arita, Tosu, Saga, Kojo), Hyogo Prefecture (Tanba, Fukuchiyama, Mita, Kobe), Wakayama Prefecture (Tanabe, Hikigawa), Shiga Prefectue, Gunnma Prefecture, Japan.

Type specimens: deposited at the National Museum of Nature and Science, Tokyo. Holotype, NSMT-As4196 (female worm); allotype, NSMT-As4195 (male worm); paratypes, NSMT-As4194, As4197, As4198, As4208–As4210, As4221–As4223, As4243–As4248, As4252, As4262, As4270–As4274, As4285–As4289.

Deposited DNA sequence: DDBJ/EMBL/GenBank accession no. LC052366–LC052374 (SSU rDNA).

Prevalence: thirty-five out of 445 (7.87%) feral alien raccoons collected in Nagasaki, Saga, and Hyogo Prefectures, Japan.

Etiology: The species is named after Mr. Kazuo Suzuki, M.Sc., a mammalogist at Hikiiwa Park Center, Tanabe, Wakayama, who has been our research collaborator for many years on wildlife mammalian parasites.

ZooBank number for species: urn:lsid:zoobank.org:act:C9673908-D764-4A3F-8F18-9AB54228037C.

### 3.3. Aonchotheca suis (Yamaguti, 1943) n. comb. (Nematoda: Trichocephalida: Capillariidae)

(Syn. *Capillaria suis* Yamaguti, 1943)

#### 3.3.1. Worm Recovery and Morphological Observation

**Figure 4:**
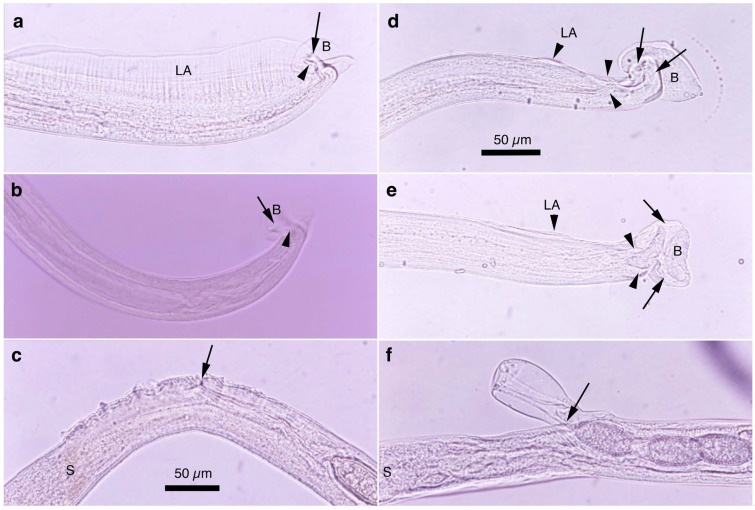
Morphology of *Aonchotheca suis* n. comb. (**a**–**c**) and *A. riukiuensis* (**d**–**f**) from the stomach of wild boars. (**a**) Caudal end of male worm with conspicuous lateral alae (LA) and membranous bursa (B) supported with two pairs of ventrolateral projections, simple digitiform ones (arrowheads) and hammer-shaped ones (arrow). (**b**) Caudal end of male worm lacking lateral alae but with membranous bursa (B) supported with two pairs of ventrolateral projections, simple digitiform ones (arrowhead) and hammer-shaped ones (arrow). (**c**) Evidently and finely roughened cuticular surface around the vulval opening (arrow). End of stichosome (S). (**d**,**e**) Caudal end of male worms with rudimentary lateral alae (LA) and membranous bursa (B) supported with two pairs of ventrolateral projections, simple digitiform ones (arrowhead) and thick hammer-shaped ones (arrow). (**f**) Bell-shaped vulval appendage at the vulval opening (arrow). End of stichosome (S). Photographs (**a**–**c**) at the same magnification, and scale is shown in (**c**). Similarly, photographs (**d**–**f**) at the same magnification, and scale bar is shown in (**d**).

This species was found in the stomachs of nine (62.3%) out of 14 wild boars (*Sus scrofa leucomystax*) collected in Hyogo Prefecture and 17 (68.0%) out of 25 wild boars in Wakayama Prefecture. Male worms exhibited well-developed caudal lateral alae with distinct striae and membranous bursa supported with two pairs of ventrolateral projections: simple digitiform projections in the anterior position and hammer-shaped projections at the posterior position (Figure 4a,b). The spicules were moderately sclerotized with a lancet-shaped tip, and the surface of the spicular sheath was non-spiny but transversely striated. The female worms had a vulva located at approximately one-third of the body length from the anterior end, with the cuticular surface around the vulva evidently and finely roughened (Figure 4c). The posterior end of the female was either bluntly rounded or conical with a terminal anus. Bioperculated barrel-shaped eggs had thick eggshells with smooth surfaces. Measurements of male and female worms are shown in Supplementary Table S3.

#### 3.3.2. Molecular Characterization

Two SSU rDNA sequences of *A. suis* collected from different hosts (accession nos. LC052375 and LC052376) were identical, spanning a partial gene sequence of 1800 bp. The highest sequence identity of *A. suis* SSU rDNA was observed in *A. riukiuensis* in the stomach of wild boars (LC052377 and LC052378): 98.44% (1770/1798) identity with two indels.

#### 3.3.3. Remarks

This species was originally described as “*Capillaria suis*” by Yamaguti in 1943 [83] using specimens collected from the stomachs of wild boars in Yamaguchi and Wakayama Prefectures, Japan. The present study confirmed that the caudal morphology of male specimens, the absence of vulval appendages, and smooth eggshell surfaces in female specimens, and morphometric values were consistent with the original description (Supplementary Table S3). However, Yamaguti [83] did not document the presence of highly developed caudal lateral alae, as observed in the present study. Among 21 male worms collected from the stomach of a wild boar in Wakayama, two lacked caudal lateral alae. Although this finding confirms the existence of individuals without these structures, as originally described by Yamaguti [83], most specimens exhibited prominently developed caudal lateral alae. This species was recorded in the stomachs of 15 (75%) of 20 and seven (78%) of nine wild boars collected in Wakayama and Hyogo Prefectures, respectively [54]. However, as demonstrated later in the present study, these previously reported specimens [54] may contain not only *A. suis* but also other *Aonchotheca* spp., such as *A. riukiuensis* and *A. suzukii* n. sp.

### 3.4. Aonchotheca riukiuensis (Shoho et Machida, 1979) Moravec, 1982

#### 3.4.1. Worm Recovery and Morphological Observation

Among the four capillariid species identified in the stomachs of wild boars, this species exhibited the highest prevalence, with an occurrence rate of 78.6% in the 14 animals collected from Hyogo Prefecture and 80.0% in the 25 animals from Wakayama Prefecture. Male worms possessed narrow caudal lateral alae with a well-developed membranous bursa supported with a pair of thick, ventrolateral, hammer-shaped projections (Figure 4d,e). Additionally, a pair of digitiform projections were present at the level of the cloaca. The thin sclerotized spicules were tapered distally, and the spicular sheath was non-spiny and transversely wrinkled. Female worms had a vulva approximately one-fifth to one-third of the body length from the anterior end. The vulval appendages were bell-shaped (Figure 4f), directed to the anterior side, and ended bluntly with the subterminal anus. The eggshell surface of the bioperculated barrel-shaped eggs was longitudinally irregularly striated and often fused to form a reticulate pattern. The measurements of male and female worms are shown in Supplementary Table S3.

#### 3.4.2. Molecular Characterization

Three SSU rDNA sequences of *A. riukiuensis* collected from different hosts (accession nos. LC052377, LC052378, and LC850897) were identical to the partial gene sequences of 1800 bp. The highest sequence identity was observed with *A. suis* as mentioned above. *Aonchotheca riukiuensis* and *A. suis* exhibited high SSU rDNA sequence identity with *A. suzukii* n. sp., identified in the stomach of wild boars, with a 98.33% (1770/1800) sequence identity when excluding nine indels and a 98.00% (1764/1800) sequence identity when excluding nine indels, respectively. These findings confirm the genetic distinction between these three species.

#### 3.4.3. Remarks

This species was originally described as “*Capillaria riukiuensis*” by Shoho and Machida [84], based on specimens from the stomach of Ryukyu wild boars (*Sus scrofa riukiuanus* Kuroda, 1924) on Iriomote Island, Okinawa Prefecture, Japan. Later, Uchida et al. [85] identified the same species in Ryukyu wild boars on Amami-Oshima Island, Kagoshima Prefecture, Japan. Previous studies on parasites of Japanese wild boars had only recorded *A. suis* [54]. However, the present study demonstrates that *A. riukiuensis* is not only distributed in the Ryukyu archipelago of Japan but also in Honshu and potentially on Kyushu and Shikoku, Japan’s other main islands. Additionally, this study clarifies that Japanese wild boars host at least four species of Capillariidae in their stomachs: *A. suis*, *A. riukiuensis*, and *A. suzukii* n. sp., which is also found in wild Carnivora mammals, as well as *Echinocoleus yokoyamae* n. sp. mentioned later.

### 3.5. Aonchotheca bilobata (Bhalerao, 1933) Moravec, 1982

(Syn. *Aonchotheca musimon* sensu Tamaru et al., 2015; *Aonchotheca musimon* sensu Sakaguchi et al., 2020).

#### 3.5.1. Worm Recovery and Morphological Observation

Fine filiform specimens were collected from the abomasum (7 male and 11 female worms) and upper small intestine (21 male and 21 female worms) of a domestic goat (*Capra hircus* Linnaeus, 1758) housed in Tokiwa Citizens’ Park, Ube City, Yamaguchi Prefecture, for temporal exhibition in a grass plot. The goat died, owing to gastric obstruction caused by a ball of entangled plastic tape in January 2009. Morphological comparisons of worms from different organs—the abomasum and the upper small intestine (mainly the duodenum)—indicated that they were morphologically identical (Supplementary Table S4).

Male worms, 12.0–13.6 mm long and 0.050–0.068 mm wide, possessed small triangular caudal lateral alae and a conspicuous membranous bursa supported with a pair of ventrolateral projections bifurcated at their ends into small nodules (Supplementary Figure S2). The spicules were well sclerotized, 0.21–0.23 mm long, widening near the distal end before tapering to a blunt tip. The spicular sheath lacked spines but exhibited a fold near the cloaca (likely corresponding to spicular sheath balloons when exerted), after the fold widening with transverse wrinkles. Female worms, 19.4–22.0 mm long and 0.064–0.086 mm wide, had a vulva positioned approximately one-third of the body length from the anterior end, with no vulval appendages but with a roughened cuticular surface around the vulva. The body ended bluntly with a subterminal anus. The lemon-shaped eggs were bioperculated, 0.054–0.063 (0.058) mm by 0.023–0.032 (0.027) mm, with striated eggshell surfaces.

#### 3.5.2. Molecular Characterization

The SSU rDNA sequence of *Aonchotheca bilobata* from a goat (accession no. LC052379), 1813 bp in length, showed high nucleotide identity with the same gene sequences of other *Aonchotheca* spp., ranging between 97.0% and 98.5%, with several indels.

#### 3.5.3. Remarks

Various *Aonchotheca* spp. have been described from the alimentary tract of ruminants [2,5,86,87,88,89,90]. Currently, three species—*A. bilobata*, *A. musimon* Pisanu et Bain, 1999, and *A. bovis* (Schnyder, 1906), remain valid following the synonymization of others, primarily based on the morphology of male caudal structures, including spicule length, spicular sheath, or caudal lateral alae, as well as site preferences (abomasum vs. upper small intestine). The specimens in this study were easily differentiated from *A. bovis* based on the distinct lengths of the spicules and caudal alae in male worms, different positions of the anus opening (subterminal vs. terminal), and different egg dimensions in female worms [88,89,90]. Pisanu and Bain [90] indicated that *A. musimon* is morphologically similar to *A. bilobata* and the present specimens, except for the different shapes and sizes of the caudal lateral alae. The molecular characterization of these two species in their type-host ruminants is highly recommended.

### 3.6. Pearsonema neoplica *n. sp.*

(Syn. *Pearsonema* sp. Toda-2010c ex. raccoon Toda et al., 2015)

#### 3.6.1. Worm Recovery and Morphological Observation

Two morphotypes of this species—distinguished by the presence or absence of a caudal triangular membranous bursa—were recovered from the urinary bladders of raccoon dogs trapped between February 2015 and July 2017 in southern Wakayama Prefecture (Table 5). The prevalence of the morphotype with triangular membranous bursa in male worms was 25.0% (8/32), while that of the morphotype lacking bursa was 18.8% (6/32). The maximum worm burden recorded was 13 (5 male and 8 female) or 12 (8 male and 4 female) worms, with other hosts carrying only a few parasites. Despite the presence or absence of a terminal triangular membranous bursa in male worms, other morphological traits were identical, suggesting conspecificity. The morphological features of the present species were similar to *Pearsonema plica* (Rudolphi, 1819) Moravec, 1982; however, the length of the spicule was distinct (≤2.0 mm in the present isolate). A molecular analysis of the SSU rDNA sequences supported the specific independence of the present new isolate.

*Pearsonema neoplica* n. sp. (Nematoda: Trichocephalida: Capillariidae)

Description: Thin filiform nematode with a rounded, narrowed head end and indistinct oral papillae. Worm body entirely covered with a transversely striated cuticle, and gradually tapered at both ends.

**Figure 5:**
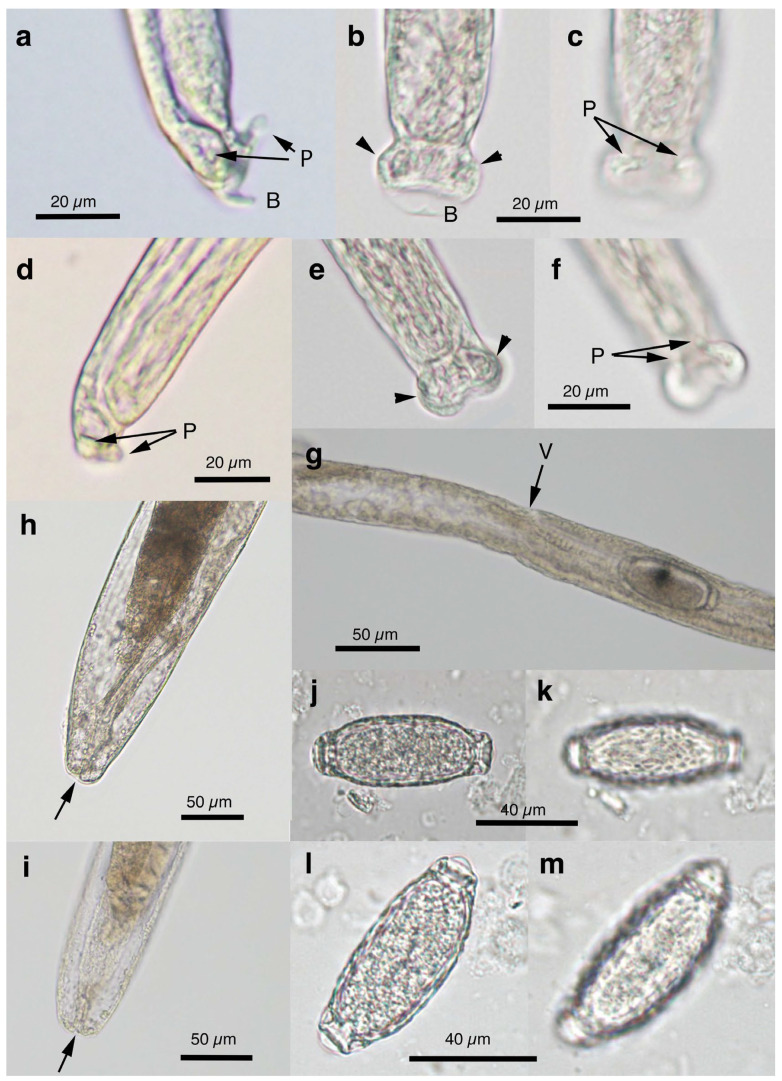
Morphology of *Pearsonema neoplica* n. sp. (**a**–**c**) Caudal end of male worm with caudal triangular membranous bursa (B) connected to dorsolateral lobes (arrowheads) and supported with digitiform projections (P). (**d**–**f**) Caudal end of male worm lacking caudal triangular membranous bursa. Dorsolateral lobes (arrowheads) and digitiform projections (P). (**g**) Vulva (V) without appendages of female worm. (**h**,**i**) Posterior end of female worms with terminal anus (arrow). (**j**–**m**) Bioperculated barrel-shaped eggs, with reticulated eggshell surface texture. Sagittal section (**j**,**l**), and reticulated eggshell surface (**k**,**m**). Scale bar is shown in each photograph.

**Figure 6:**
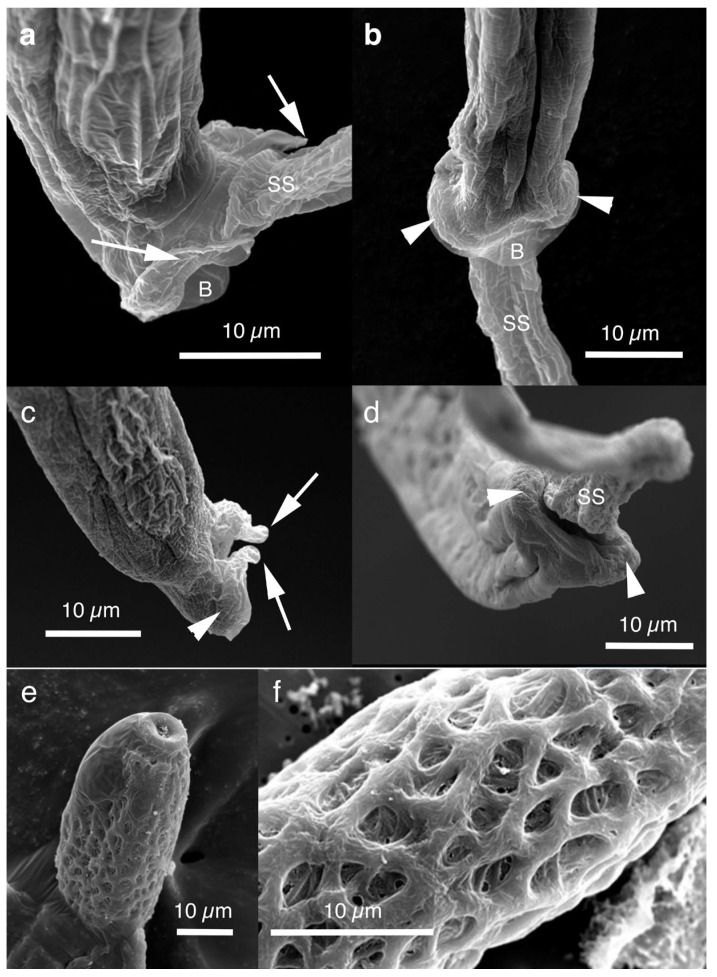
Scanning electron microscopic view of *Pearsonema neoplica* n. sp. (**a**,**b**) Caudal end of male worm with caudal triangular membranous bursa (B) connected to dorsolateral lobes (arrowheads) and supported with digitiform projections (arrows). Extruded spicular sheath (SS) from cloaca. (**c**,**d**) Caudal end of male worm lacking caudal triangular membranous bursa. Dorsolateral lobes (arrowheads) and digitiform projections (arrows). Extruded spicular sheath (SS) from cloaca. (**e**,**f**) Bioperculated barrel-shaped eggs, with reticulated eggshell surface texture. Scale bar is shown in each photograph.

**Figure 7:**
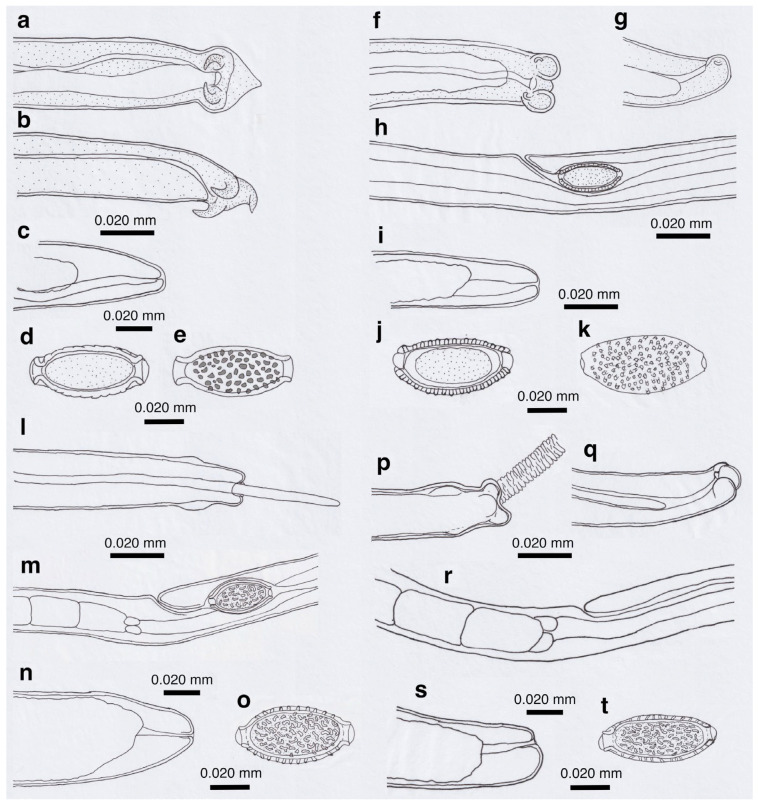
Line drawings of *Pearsonema neoplica* n. sp. (**a**–**e**), *P. feliscati* (**f**–**k**), *P iharai* n. sp. (**l**–**o**), and *P. toriii* n. sp. (**p**,**q**). Caudal end of male worms (**a**,**b**,**f**,**g**,**l**,**p**,**q**), vulval region of female worms (**h**,**m**,**r**), posterior end of female worms (**c**,**i**,**n**,**s**), sagittal section of eggs (**d**,**j**), eggshell surface (**e**,**k**), and jointed view of sagittal egg section and eggshell surface (**o**,**t**).

Male (*n* = 4): Body length of 19.29–23.85 (20.65) mm, maximum body width of 0.052–0.061 (0.056) mm around the middle of the body near the end of the esophagus (stichosome) situated 4.97–7.39 (6.25) mm from the head end. The ratio of the posterior part after the esophageal end to the anterior part, 1.61–3.80 (2.41). The posterior extremity of the body without caudal lateral alae ended in the dorsolateral lobes with a triangular membranous bursa supported with a pair of relatively long digitiform projections on the dorsolateral lobes (Figure 5a–c, Figure 6a,b and Figure 7a,b). Male worms frequently lacked caudal triangular membranous bursa (Figure 5d–f and Figure 6c,d). Thin spicules 1.55–1.89 (1.78) mm in length, and spicular sheaths non-spiny and transversely striated.

Female (*n* = 7): Female worms longer than male worms, 20.45–30.63 (26.34) mm long, and the maximum body width of 0.070–0.103 (0.082) mm around the mid-uterine part. End of the stichosome at 6.59–8.31 (7.79) mm from the anterior end, and the posterior part after the esophageal end longer than the anterior part by a ratio of 2.06–2.97 (2.39). Vulva ventrally opened at the level of the esophageal end but posteriorly at a distance of 0.052–0.235 (0.107) mm. No vulval appendages (Figure 5g). The posterior extremities ended bluntly with the terminal anus (Figure 5h and Figure 7c). Eggshell surface of bioperculated barrel-shaped eggs, 0.060–0.068 (0.064) mm by 0.027–0.030 (0.028) mm, with a reticulated eggshell surface texture (Figure 5j,k, Figure 6e,f and Figure 7d,e).

Alternative form without caudal triangular membranous bursa in male worms:

Thin filiform nematode, gradually tapered to both ends, exhibiting the defining morphological features of *P. neoplica* n. sp., except for the absence of a triangular membranous bursa at the caudal end of male worms.

Male (*n* = 8): Filiform, 16.76–22.21 (20.60) mm long and 0.044–0.057 (0.051) mm in maximum width, with no caudal lateral alae, ended in dorsolateral lobes with small membranous bursa between lobes, on which long digitiform projections (Figure 5d–f and Figure 6c,d). Thin spicules 1.47–1.80 (1.61) mm in length, and spicular sheaths non-spiny and irregularly wrinkled.

Female (*n* = 9): Female worms larger than male worms, measuring 19.96–26.69 (21.80) mm long and 0.067–0.087 (0.079) mm in maximum width. End of the stichosome at 7.15–9.84 (8.10) mm from the anterior end, and the posterior part after the esophageal end longer than the anterior part by a ratio of 1.48–2.02 (1.73). Vulva ventrally opened at the level of the esophageal end but posteriorly at a distance of 0.058–0.208 (0.128) mm. No vulval appendages. The posterior extremities ended bluntly with the terminal anus (Figure 5i). Eggshell surface of bioperculated barrel-shaped eggs, 0.064–0.073 (0.068) mm by 0.026–0.029 (0.028) mm, with a reticulated eggshell surface (Figure 5l,m).

#### 3.6.2. Molecular Characterization

The SSU rDNA sequences of four *P. neoplica* n. sp. worms isolated from the urinary bladders of four raccoon dogs trapped in February 2015 in Wakayama Prefecture were identical (accession no. LC858133–LC858136; 1808 bp). This sequence was nearly identical to that of *P. neoplica* n. sp. from a feral alien raccoon trapped in August 2009 in Hyogo Prefecture (LC052390), with an identity of 99.61% (1801/1808). Furthermore, *P. neoplica* n. sp. sequences from urinary capillariid worms from raccoon dogs and a raccoon exhibited 98.97% (1725/1743) or 99.25% (1730/1743) identities with *P. plica* from the urinary bladder of a red fox in western Germany (Rhineland-Palatinate) (MF621034). Several short SSU rDNA sequences of *P. plica* in the urinary bladders of red foxes, golden jackals, European wildcats, and wolves (JX456614–JX456623, KX962319, KX962320, KX962326–KX962328, KX962336, KX962352, OP159859, and OP159919; 528–598 bp), corresponded to a partial sequence between nucleotides 1031 and 1641 of the *P. neoplica* n. sp. SSU rDNA sequence (LC858133) and were identical to those of both *P. plica* (MF621034) and *P. neoplica* n. sp. (LC858133–LC858136 and LC052390).

#### 3.6.3. Remarks

The newly described species is morphologically similar to *P. plica*, a globally distributed species isolated from the urinary bladder of various Carnivora mammals such as wolves, foxes, golden jackals, dogs, martens, badgers, raccoons, raccoon dogs, and brown bears (*Ursus arctos* Linnaeus, 1758) [3,4,43,45,66,68,69,70,91,92]. Male worms of both species have caudal ends with triangular membranous bursa, supported with a pair of fairly long digitiform projections on the dorsolateral lobes (Figure 5, Figure 6 and Figure 7a–e). Female worms produce bioperculated barrel-shaped eggs with reticulated eggshell surface texture, a key morphological hallmark for diagnosing *P. plica* infection in wild canids and clinical canine and feline cases [35,39,41,42,43,44,45,47,49]. Rarely, eggshell surface texture distinct from those of *P. plica* and *P. neoplica* n. sp., resembling the vermiculated eggshell surface texture of *Pearsonema iharai* n. sp. or *P. toriii* n. sp. eggs, were reported as *P. plica* eggs (e.g., Figure 2 of Bork-Mimm and Rinder [36]). Typical *P. plica* male worms have a long filiform spicule (>2.0 mm), and female worms have a cylindrical vulval appendage [2,3,4], in contrast to *P. neoplica* n. sp. described in this study (Table 6). The morphological separation of *P. neoplica* n. sp. from *P. plica*, prevalent in red foxes in Europe, was supported with molecular genetic analyses of the SSU rDNA (Table 7). As discussed above for *A. putorii*, which comprises four major genotypes, currently recognized *P. plica* likely consists of multiple morphotypes and genotypes. To clarify their taxonomic status, further genetic studies on urinary bladder worm specimens—both adult worms and eggs—are essential. When using the SSU rDNA gene for this purpose, the sequences must contain hypervariable regions of nucleotide changes necessary for species-level identification of capillariid worms (Table 7).

The newly described species is morphologically differentiated from other urinary bladder worms of the genus *Pearsonema*, including *P. feliscati* (Diesing, 1851) Freitas et Mendonça, 1960 from felids, such as domestic cats, *P. linsi* (Freitas et Lent, 1935) Freitas et Mendoca, 1960, from *Grison vittata* Schreber, 1776 (Mustelidae) in Brazil, *P. mucronate* (Molin, 1858) Moravec, 1982 from various musterids in Europe, and *P. pearsoni* Freitas et Mendonça, 1960 from *Procyon cancrivorous* in Brazil [2,3,4,93] (Supplementary Table S5).

#### 3.6.4. Taxonomic Summary

Type host: *Nyctereutes viverrinus viverrinus* (Temmink, 1838) (Carnivora: Canidae), native raccoon dogs in Japan.

Additional hosts: *Procyon lotor* (Linnaeus, 1758) (Carnivora: Procyonidae), feral alien raccoons in Japan, introduced originally from North America.

Site of infection: urinary bladder.

Type locality: Wakayama Prefecture (Tanabe, Shirahama, Minabe), Japan.

Additional localities: Hyogo Prefecture (Mita, Kobe), Japan.

Type specimens: deposited at the National Museum of Nature and Science, Tokyo. Holotype, NSMT-As4504 (male worm); allotype, NSMT-As4505 (female worm); para-types, NSMT-As4506–As4515 (male worms with triangular membranous bursa, and female worms), and As4516–As4521 (male worms without triangular membranous bursa, and female worms).

Deposited DNA sequence: DDBJ/EMBL/GenBank accession nos. LC052390, and LC858133–LC858136 (SSU rDNA).

Prevalence: 7 out of 32 (21.9%) raccoon dogs collected in Wakayama Prefecture, Japan. Prevalence of *Pearsonema* spp. (*P. neoplica* n. sp. and *P. feliscati*) in feral alien raccoons in Hyogo and Wakayama Prefectures were 5.8% (9/154) and 5.5% (26/477), respectively.

Etiology: The new species (*P. neoplica* n. sp.) is named after its close resemblance in morphology to *Pearsonema plica* except for shorter spicules and lack of cylindrical vaginal appendages.

ZooBank number for species: urn:lsid:zoobank.org:act:916C1D05-2611-4413-A4A8-95C03D5FDBC4.

### 3.7. Pearsonema feliscati (Diesing, 1851) Freitas et Mendonça, 1960

#### 3.7.1. Worm Recovery and Morphological Observation

This species was found in the urinary bladder of 5.5% (26/477) feral alien raccoons trapped between February 2015 and July 2017 in the southern part of Wakayama Prefecture. The average intensity was 1.7 worms per animal.

**Figure 8:**
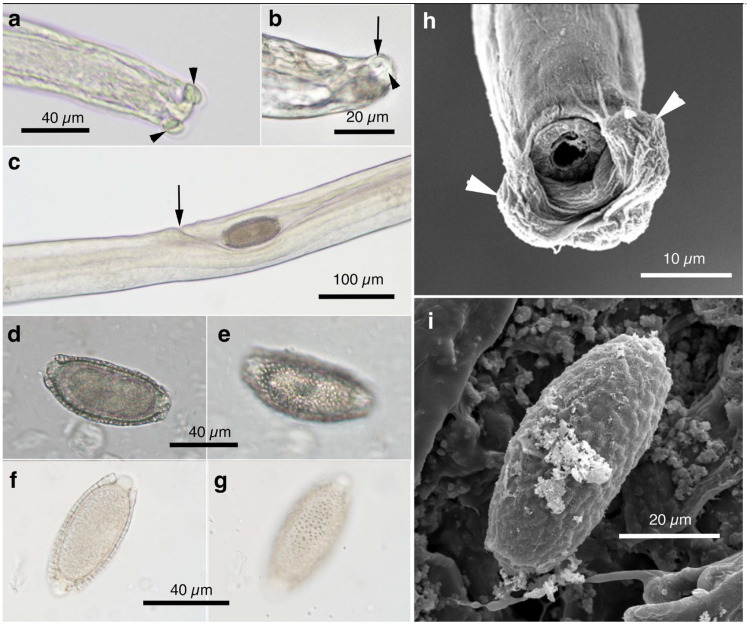
Morphology of *Pearsonema feliscati*. (**a**,**b**) Ventral and lateral views of caudal end of male worms, lacking apparent membranous bursa. Dorsolateral lobes (arrowheads) and a nodular projection (arrow). (**c**) Vulva (arrow) without appendages of female worm. (**d**–**g**) Bioperculated barrel-shaped egg, with punctuated eggshell surface texture, collected from urine of a feral alien raccoon (**d**,**e**), or a domestic cat (**f**,**g**). (**h**) Scanning electron microscopic view of male caudal end with dorsolateral lobes (arrowheads) in ventral view. (**i**) Scanning electron microscopic view of *P. feliscati* egg surface. Scale bar is shown in each photograph.

Male filiform worms (*n* = 4), 20.54–24.23 (22.27) mm long and 0.046–0.055 (0.050) mm in maximum width, had no caudal lateral alae and ended in a pair of dorsolateral lobes on which minute vented projections were localized ventrolaterally (Figure 7f,g and Figure 8a,b,h). The esophagus measured 4.06–7.17 (6.04) mm, and the ratio of the posterior part after the esophageal end to the anterior part was 2.32–4.33 (2.85). Thin spicules measured 2.22–2.46 (2.33) mm in length, and spicular sheaths were non-spiny and transversely wrinkled.

Female filiform worms (*n* = 10) were larger than male worms, measuring 13.32–34.70 (22.91) mm long and 0.047–0.108 (0.081) mm in maximum width. The end of the stichosome was at 5.22–13.48 (9.22) mm from the anterior end, and the posterior part after the esophageal end was longer than the anterior part, with a ratio of 1.21–2.08 (1.52). The vulva opened ventrally at the level of the esophageal end but posteriorly at a distance of 0.073–0.525 (0.248) mm (Figure 7h and Figure 8c). No vulval appendages were observed. The posterior extremities ended bluntly with a terminal anus (Figure 7i). Eggshell surfaces of the bioperculated barrel-shaped eggs, 0.057–0.078 (0.063) mm by 0.022–0.031 (0.026) mm, were punctuated (Figure 7j,k and Figure 8d,e,i). In cross-section, eggshells showed a palisade or striated texture. The measurements of the male and female worms are compared with those previously recorded by Freitas and Lent [1] in Table 6.

#### 3.7.2. Eggs Excreted in the Urine of a Cat

A mongrel cat (1.5 years old, female) was examined for anorexia at a veterinary clinic in Tokyo in November 2020, and a laboratory examination revealed capillariid eggs in the urine. Bioperculated barrel-shaped eggs (*n* = 10) measured 0.067–0.072 (0.070) mm by 0.029–0.031 (0.030) mm, had a punctuated surface, and were striated in the sagittal section (Figure 8f,g). The eggshell length without plug protrusions was 0.059–0.064 (0.062) mm, and plugs measured 6.7–9.2 (7.5) µm by 7.5–8.8 (8.1) µm. Another cat case (details unknown) in Nara in August 2022 excreted similar eggs with a punctuated eggshell surface in the urine.

#### 3.7.3. Molecular Characterization

The SSU rDNA sequences (1824 bp and 1827bp) of two *P. feliscati* worms from the urinary bladders of two raccoons trapped in July 2009 in Hyogo Prefecture were identical, excluding a site of three indels (accession nos. LC052388 and LC052389). The SSU rDNA sequences (1827bp) of three *P. feliscati* worms from the urinary bladders of three raccoons trapped between February and March 2015 in Wakayama Prefecture were nearly identical to the sequences mentioned above (LC052389), with 99.95% (1826/1827) –100% identities. These sequences were identical to the SSU rDNA sequences of *P. feliscati* eggs in the urine of domestic cats from two cases in Tokyo and Nara Prefectures (LC850894). A partial SSU rDNA sequence (ON390990; 563-bp) of immature *Pearsonema* eggs in the urine sediment of a cat from Sri Lanka was identical to those of *P. feliscati* reported in this study (LC052388, LC052389. LC850894, and LC858139) and distinct from those of *P. plica* (MF621034, JX456614–JX456624, KX962319, KX962320, KX962326–KX962328, KX962336, KX962352, OP159859, and OP159919) or *P. neoplica* n. sp. (LC052390 and LC858133–LC858136) with approximately 98.94% (558/564) identity and four indels.

#### 3.7.4. Remarks

*Pearsonema feliscati* is a urinary bladder capillariid worm that is found in domestic cats and other felids. It is characterized by male worms lacking a triangular membranous bursa but possessing minute bent ventrolateral projections on caudal lobes, as well as bioperculated eggs with punctuated eggshell surfaces or striated eggshell in cross-section [1,3,34,94,95,96]. Although the life cycle of *P. feliscati* has not been fully documented, it is speculated to be indirect, with the earthworm serving as an intermediate host, similar to *P. plica* and *P. mucronate* [47,77,94]. Wilson-Hanson and Prescott [96] conducted a 12-month survey based on postmortem findings from 400 cats of varying ages in Brisbane, Queensland, Australia, in 1979, revealing an 18.3% (20/108) prevalence of *P. feliscati* infections in cats older than two years, 5.6% (4/71) in 10–15-month-old cats, and 0.0045% (1/221) in cats younger than 8 months. The maximum number of adult worms found in the urinary bladder was 25. Prior to that study, Waddell [94,95] reported 34% (17/50) and 31% (31/100) *P. feliscati* infections in necropsied cats of mixed ages in Brisbane in the late 1960s, with a maximum recovery of 19 worms.

Capillariid worms collected from feral alien raccoons were morphologically congruent with male and female *P. feliscati* worms, and mature eggs had a punctuated eggshell surface texture, typical of *P. feliscati* [1,34,94]. In the present study, similar eggs were collected from the urine of two domestic cats with cystitis, and parasite samples from raccoons (adult worms) and cats (eggs) showed identical SSU rDNA sequences. De Silva et al. [97] obtained the same SSU rDNA sequence from immature capillariid eggs in the urine of a cat with cystitis in Sri Lanka, suggesting that urinary bladder worms collected from feral alien raccoons in Japan are *P. feliscati*, a parasite prevalent in cats and other felids worldwide. Butterworth and Beverley-Burton [4] reported a high prevalence of *P. plica* in native raccoons from North America (71.7% (38/53) in juveniles and 91.4% (64/70) in adults), whereas Heddergott et al. [98] reported a low prevalence (6.2% [31/499]) of *P. plica* infection in feral introduced raccoons in Central Europe. In Japan, *P. feliscati* was recorded in eight out of 37 native raccoon dogs in Kanagawa Prefecture (intensity ranged between two and 17) and in three out of eight raccoon dogs in Tokyo Prefecture (intensity between two and six) [79], in addition to domestic cats [99]. To the best of our knowledge, feral alien raccoons are a new host record for *P. feliscati*.

### 3.8. Pearsonema iharai *n. sp.*

(Syn. *Pearsonema* sp. Toda-2010a ex. mink Toda et al., 2015)

#### 3.8.1. Worm Recovery and Morphological Observation

Urinary bladder capillariid worms were highly prevalent in feral alien American minks trapped in Koriyama, Fukushima Prefecture, Japan, at a rate of 31 (40.8%) of 76 feral alien American minks collected from August to October 2010. The infection intensity ranged from one to 11 worms (3.87 worms on average and 2.94 worms in geometric mean). In total, almost equal numbers of male and female worms were collected (62 male worms, and 58 female worms).

**Figure 9:**
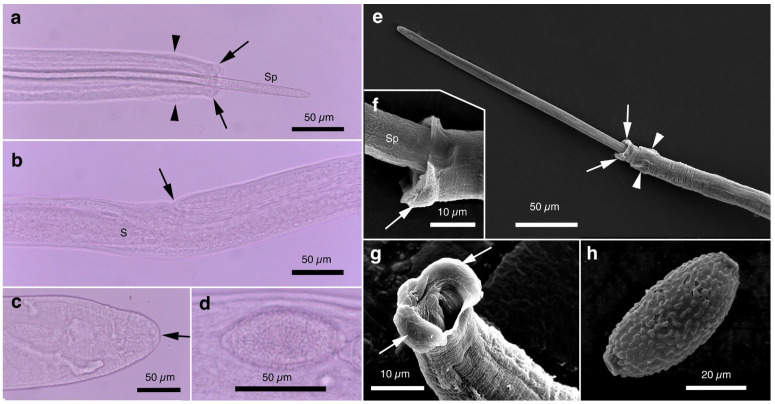
Morphology of *Pearsonema iharai* n. sp. (**a**) Ventral view of caudal end of male worm, lacking apparent membranous bursa. Circular cuticular swelling (arrowheads) before caudal end with dorsolateral lobes (arrows). Protruded spicule (Sp). (**b**) Vulva (arrow) without appendages of female worm. (**c**) Caudal end of female worm with a terminal anus (arrow). (**d**) Intrauterine bioperculated lemon-shaped egg, with vermiculated eggshell surface texture. (**e**–**g**) Scanning electron microscopic view of male caudal end with dorsolateral lobes (arrows) in ventral view. Circular cuticular swelling (arrowheads) prior to the caudal end. (**h**) Scanning electron microscopic view of *P. iharai* n. sp. egg with vermiculated eggshell surface. Scale bar is shown in each photograph.

In this study, the filiform capillariid worms from the urinary bladders of minks were thicker than *P. neoplica* n. sp. and *P. feliscati* worms from the urinary bladders of raccoon dogs and raccoons, respectively (Table 8). Furthermore, the texture of the eggshell surface of mink urinary bladder worms was distinct from *P. plica*, *P. neoplica* n. sp. (reticulated), and *P. feliscati* (punctuated), showing a vermiculated pattern (Figure 7o and Figure 9d,h).

##### *Pearsonema iharai* n. sp. (Nematoda: Trichocephalida: Capillariidae)

Description: Thin filiform nematode with a rounded, narrowed head end and indistinct oral papillae. Worm body entirely covered by a transversely striated cuticle, and gradually tapered at both ends.

Male (*n* = 10): Body length of 16.19–26.19 (21.09) mm, maximum body width of 0.054–0.070 (0.065) mm. Anterior body with a short muscular esophagus and a single row of stichocytes (stichosome), gradually increased in its width, ended at 4.90–6.49 (5.80) mm from the head end. Ratio of the posterior to anterior body part, 1.99–3.12 (2.62). Caudal end terminated with small ventrolateral lobes lacking digitiform projections (Figure 7l and Figure 9a,e–g). Lateral cuticular swelling near the caudal end. Filiform spicules measured 1.60–2.46 (2.18) mm long, and spicular sheaths non-spiny and transversely striated.

Female (*n* = 11): Female worms bigger than male worms, 18.68–29.07 (23.85) mm in length, and 0.104–0.148 (0.118) mm in maximum width. Anterior body with a short muscular esophagus and a single row of stichocytes (stichosome), gradually increased in its width, ended at 7.45–9.40 (8.24) mm from the anterior end. Ratio of the posterior to anterior body, 1.41–2.24 (1.89). Vulva situated at 0.071–0.148 (0.118) mm posterior to esophageal end, without vulvar appendages (Figure 7m and Figure 9b). Posterior body bluntly ended with terminal anus (Figure 7n and Figure 9c). Bioperculated lemon-shaped eggs 0.064–0.075 (0.069) by 0.026–0.034 (0.030) mm, with vermiculated eggshell surfaces, like a bitter melon surface (Figure 7o and Figure 9d,h).

#### 3.8.2. Molecular Characterization

The SSU rDNA sequence (1810 bp) of this species from the urinary bladder of a feral alien American mink (accession no. LC052386) differed from that of *P. plica* from a red fox in Germany (MF621034) with 98.51% (1714/1740) identity and eight indels. Comparison with *P. neoplica* n. sp. from raccoon dogs (LC858133–LC858136) and a raccoon (LC052390) revealed sequence identities of 98.72% (1777/1800) to 98.84% (1784/1805) with eight indels, while *P. feliscati* from raccoons and cats (LC052388, LC052389, LC850894, and LC858137–LC858139) showed sequence identities of 98.28% (1773/1804) to 98.34% (1780/ 1810), also with eight indels. The newly identified species demonstrated a comparable level of genetic divergence from *P. plica*, *P. neoplica* n. sp., and *P. feliscati.* The highest nucleotide sequence identity, 99.83% (1807/1810), was observed between the present isolate from a feral alien mink in Fukushima Prefecture (LC052386) and another isolate from the urinary bladder of a native Japanese weasel from Yamaguchi Prefecture (LC850896). These two sequences exhibited a six-nucleotide deletion at sites 226–231 from the 5′-terminus of LC850896, a sequence from a capillariid species from a native weasel. This Japanese weasel was collected after being killed in a traffic accident in March 2021 near Yamaguchi University. Because only a female adult worm was recovered, morphological characterization had not been conducted before DNA extraction, except for the vermiculated eggshell surface texture. Three nucleotide substitutions were detected between these sequences around the indel site.

#### 3.8.3. Remarks

The morphology of the caudal end of the newly identified species was distinct from that of the genus *Pearsonema* in the absence of digitiform projections originating from the two dorsolateral lobes located at the caudal end. Despite being collected from the urinary bladders of mustelids, the species exhibited morphological characteristics aligning more closely with the genus *Liniscus* Dujardin, 1845, which is defined by male worms possessing two simple, rounded dorsolateral lobes at the caudal end [5]. Capillariid parasites from the urinary bladder of Carnivora mammals have been classified in the genus *Pearsonema*. In contrast, those in the urinary bladder, ureter, and kidneys of rodents, soricids, and talpids have been classified in the genus *Liniscus* with *L. incrassatus* Diesing, 1851, from *Rattus* spp., being the type species. Moravec [5] previously noticed that the lack of sufficient information on the male caudal ends of the species listed in the genus *Liniscus* (then eight species) necessitated a future redefinition of the genus. In 2008, Robles et al. [100] reported a new species of *Liniscus* (*Liniscus diazae*) from the urinary bladders of two Neotropical rodents (*Oxymycterus rufus* (Fischer, 1814) and *Akodon azarae* (Fischer, 1829)) using SEM. They confirmed most of the generic features proposed by Moravec [5] in 1982 and observed several new features. They redefined the genus as follows: “the tails of males possess two paracloacal lateral lobes, each with one or two papillae; dorsally, the lobes are joined by a thin fold; caudal and lateral alae are absent; and the spicular sheath is not spinose and is folded. The vulva may be elevated, or not.” [100]. The present species shared the lack of digitiform projections observed in the genus definitions by Moravec [5] and Robles et al. [100]. Although the new species showed morphological fitness to the genus *Liniscus*, its SSU rDNA sequences formed a clade with *P. plica*, *P. neoplica* n. sp., and *P. feliscati*, but not with *Liniscus himizu* (Ohbayashi, Masegi et Kubota, 1972) Moravec, 1982, mentioned latter. Therefore, the new species is classified in the genus *Pearsonema* and designated *P. iharai* n. sp.

#### 3.8.4. Taxonomic Summary

Type host: *Neogale vison* (Schreber, 1777) (Carnivora: Mustelidae), feral alien American mink in Japan, introduced originally from North America.

Additional hosts: *Mustela itatsi* Temminck, 1844 (Carnivora: Mustelidae), Japanese weasel.

Site of infection: urinary bladder.

Type locality: Fukushima Prefecture (Koriyama), Japan.

Additional localities: Yamaguchi Prefecture (Yamaguchi), Japan.

Type specimens: deposited at the National Museum of Nature and Science, Tokyo. Holotype, NSMT-As4089a (male worm); allotype, NSMT-As4089b (female worm); paratypes, NSMT-As4090–As4124.

Deposited DNA sequence: DDBJ/EMBL/GenBank accession no. LC052386, and LC850896 (SSU rDNA) from a feral alien American mink and native Japanese weasel, respectively.

Prevalence: 31 out of 76 (40.8%) feral alien American minks examined.

Infection intensity: 1–11 worms (3.87 worms in average, and 2.94 worms in geometric mean).

Etiology: The species is named after Dr. Sadao Ihara, a mammalogist at Ohu University, Fukushima, at the time of mink collection, and currently at Hokkaido University of Education, who has been our research collaborator for many years on wildlife mammalian parasites.

ZooBank number for species: urn:lsid:zoobank.org:act:FDEA4292-DEFB-4845-920C-B499C97A0B27.

### 3.9. Pearsonema toriii *n. sp.*

(Syn. *Pearsonema* sp. Toda-2010a ex. badger Toda et al., 2015)

#### 3.9.1. Worm Recovery and Morphological Observation

Slender capillariid worms were collected from 13 (44.8%) of 29 urinary bladders of Japanese badgers collected between March 2015 and December 2016 in southern Wakayama Prefecture. Preliminary morphological observations indicated that the urinary bladder worms from these native badgers were similar to *P. iharai* n. sp. However, the SSU rDNA sequences of these two isolates showed < 98.7% identity, with multiple indels, indicating their distinction as separate species.

*Pearsonema toriii* n. sp. (Nematoda: Trichocephalida: Capillariidae)

Description: Thin filiform nematode with a rounded, narrowed head end and indistinct oral papillae. Worm body entirely covered with a transversely striated cuticle, and gradually tapered at both ends.

**Figure 10:**
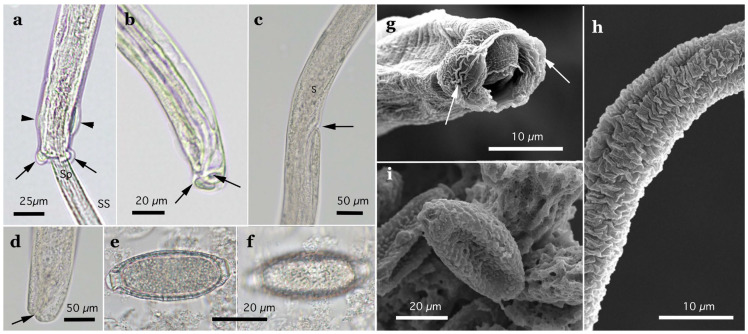
Morphology of *Pearsonema toriii* n. sp. (**a**) Dorsal view of caudal end of male worm, lacking apparent membranous bursa. Lateral cuticular swelling (arrowheads) before caudal end with conspicuous spheroid dorsolateral lobes (arrows). Protruded spicular sheath (SS) and spicule (Sp). (**b**) Lateral view of caudal end of male worm. Dorsolateral lobes (arrows). (**c**) Vulva (arrow) without appendages of female worm. End of stichosome (S). (**d**) Caudal end of female worm with subterminal anus (arrow). (**e**,**f**) Bioperculated barrel-shaped egg, with vermiculated eggshell surface texture. (**g**) Scanning electron microscopic view of male caudal end with spheroid dorsolateral lobes (arrows). (**h**) Scanning electron microscopic view of transversely wrinkled spicular sheath. (**i**) Scanning electron microscopic view of *P. toriii* n. sp. egg with vermiculated eggshell surface. Scale bar is shown in each photograph.

Male (*n* = 4): Body length of 14.48–18.82 (15.86) mm, maximum body width of 0.035–0.049 (0.043) mm. Anterior body with short muscular esophagus and a single row of stichocytes (stichosome), gradually increased in its width and ended at 3.85–5.46 (4.91) mm from the head end. Ratio of the posterior to anterior body part, 1.73–2.76 (2.27). Caudal end terminated with spheroid ventrolateral lobes lacking digitiform projections (Figure 7p,q and Figure 10a,b,g). Lateral cuticular swelling of body close to the caudal end (Figure 7p and Figure 10a). Filiform spicules measured 1.95–2.10 (2.03) mm long and spicular sheaths non-spiny and transversely wrinkled (Figure 10a,h).

Female (*n* = 7): Female worms bigger than male worms, 18.32–26.53 (23.50) mm in length, and 0.080–0.120 (0.103) mm in maximum width. Anterior body with a short muscular esophagus and a single row of stichocytes (stichosome), gradually increased in its width ended at 7.29–9.02 (8.08) mm from the anterior end. Ratio of the posterior to anterior body, 1.50–2.27 (1.91). Vulva situated 0.214–0.667 (0.346) mm posterior to the esophageal end, without vulvar appendages (Figure 7r and Figure 10c). Posterior body abruptly ended, forming a trapezoidal appearance with a subterminal anus (Figure 7s and Figure 10d). Bioperculated lemon-shaped eggs 0.058–0.074 (0.067) by 0.027–0.031 (0.029) mm, with a vermiculated eggshell surface resembling a bitter melon surface (Figure 7t and Figure 10e,f,i).

#### 3.9.2. Molecular Characterization

The SSU rDNA sequence (1813 bp) of *P. toriii* n. sp. isolated from the urinary bladder of a native Japanese badger (accession nos. LC052387) differed from those of morphologically closer *P. iharai* n. sp. from a feral alien American mink and native Japanese weasel (LC052386 and LC850896, respectively), with 98.62% (1785/1810) identity and three indels and 98.29% (1780/1811) identity and seven indels, respectively. The molecular relationships among the five *Pearsonema* spp. based on SSU rDNA sequences are shown in Table 9.

#### 3.9.3. Remarks

The two newly described *P. iharai* n. sp. and *P. toriii* n. sp. from the urinary bladders of American minks and Japanese badgers are morphologically similar to each other, except for minor differences in body sizes and morphology of the caudal end of male (simple vs. spheroid dorsolateral lobes) and female worms (terminal vs. subterminal anus). Despite their apparent resemblance, including caudal cuticular swelling in male worms, molecular genetic analysis of the SSU rDNA sequences indicates their genetic distinction from each other, as well as from *P. plica*, *P. neoplica* n. sp., and *P. feliscati* (Table 9).

#### 3.9.4. Taxonomic Summary

Type host: *Meles anakuma* Temminck, 1842 (Carnivora: Mustelidae), Japanese badger.

Site of infection: urinary bladder.

Type locality: Wakayama Prefecture (Minabe), Japan.

Type specimens: deposited at the National Museum of Nature and Science, Tokyo. Holotype, NSMT-As4550 (female worm); allotype, NSMT-As4554 (male worm); paratypes, NSMT-As4551–As4553, and As4555–As4557.

Deposited DNA sequence: DDBJ/EMBL/GenBank accession no. LC052387 (SSU rDNA).

Prevalence: 13 (44.8%) of 29 Japanese badgers examined.

Infection intensity: a few worms up to three worms/animal.

Etiology: The species is named after Dr. Harumi Torii, a mammalogist at Kyoto University of Education, who has been our research collaborator for many years on wildlife mammalian parasites.

ZooBank number for species: urn:lsid:zoobank.org:act:A4789A0F-0361-4FF4-9358-CCFD99DE4560.

### 3.10. Liniscus himizu (Ohbayashi, Masegi et Kubota, 1972) Moravec, 1982

#### 3.10.1. Worm Recovery and Morphological Observation

A dead Japanese shrew mole, *Urotrichus talpoides* Temminck, 1841 was discovered on a roadside adjacent to paddy fields in a rural area of Yamaguchi City on 29 September 2022. A single filiform worm was collected from the urinary bladder. Because the male urinary bladder worm was destroyed during microscopic observation, only fragmental information was available: maximum body width, 0.054 mm; spicule, 0.372 mm in length and 0.009 mm in width; and an aspinose spicular sheath with conspicuous transverse striations (Supplementary Figure S3). The simple caudal end had no dorsolateral lobes or projections. These morphological features were congruent with the description of *L. himizu* collected from a Japanese shrew mole from Central Japan (Saiko, Yamanashi Prefecture) by Ohbayashi et al. [101].

#### 3.10.2. Molecular Characterization

The newly obtained SSU rDNA sequences (1798 bp) of a male *L. himizu* worm in this study (accession no. LC850895) exhibited the highest identity with *P. neoplica* n. sp. isolated from raccoon dogs (LC858133–LC858136) and raccoons (LC052390); 97.77% (1756/1796)–98.05% (1761/1796) with seven indels. The SSU rDNA sequence of *L. himizu* showed identities of 98.05% (1759/1794) with 13 indels and 97.88% (1756/1794) with 19 indels when compared to *P. iharai* n. sp. from the urinary bladder of feral alien American mink (LC052386) and Japanese weasel (LC850896), respectively. Similarly, an identity of 97.16% (1744/1795) with 14 indels was observed with *P. toriii* n. sp. (LC052387).

#### 3.10.3. Remarks

The taxonomic position of mammalian urinary bladder worms lacking membranous pseudobursa and caudal projections, such as *P. iharai* n. sp. and *P. toriii* n. sp., remain uncertain, as their morphological characteristics do not appear congruent with the generic definition of *Pearsonema* [5]. Therefore, molecular genetic information, specifically the SSU rDNA sequence of *L. himizu,* is of significant importance. As demonstrated latter, the two new *Pearsonema* spp. of uncertain taxonomic position based on morphology formed a robust clade with *P. plica*, *P. neoplica* n. sp., and *P. feliscati*, but not with *L. himizu*. Further collection of molecular genetic data of *Liniscus* spp. could elucidate the phylogenetic relationships between the two genera of capillariid urinary bladder worms from Carnivora mammals (*Pearsonema*) and rodents, soricids, and talpids (*Liniscus*) [5,11].

### 3.11. Calodium hepaticum (Bancroft, 1893) Moravec, 1982

#### 3.11.1. Worm Recovery and Morphological Observation

All nine brown rats (*Ratus norvegicus* (Berkenhout, 1769)) collected from Surabaya, Indonesia, exhibited dispersed milky white patchy lesions on the liver surface (Supplementary Figure S4a). Histological analysis revealed numerous deposited eggs aggregated in the fibrotic tissues, accompanied by the infiltration of fewer inflammatory cells, including lymphocytes, plasma cells, and macrophages, indicative of chronic granulomatous responses to infection (Supplementary Figure S4b). Most worms detected in these lesions were degenerative. Isolated dark eggs were bioperculated and oval in shape (Supplementary Figure S4c), measuring 0.053–0.057 (0.055) mm by 0.027–0.035 (0.033) mm. The eggshell comprised four layers, that is, a thin layer covering the entire structure and three layers, of which the middle layer was blackish and contained one to four cells. The external surfaces of the eggshells were punctuated.

#### 3.11.2. Molecular Characterization

The SSU rDNA sequences (accession no. LC425008; 1809 bp) of *C. hepaticum* eggs from the liver of a brown rat in Surabaya showed the highest identities with capillariid worms belonging to the genera *Aonchotheca* and *Pearsonema*. However, when the entire sequence was compared, the BLAST analysis (https://blast.ncbi.nlm.nih.gov/Blast.cgi accessed on 24 December 2024) revealed up to 98.0% identity, accompanied with a few to several indels. Partial SSU rDNA sequences of *C. hepaticum* from various rodents and a fox in Europe, Argentina, and India (accession nos. JX456631, JX456635, KT875351, KY488184, MF287972, MG686613, OR135793–OR135795, and PQ241654–PQ241656; 539–635 bp) were identical to the sequence (LC425008) obtained in this study, whereas a lower identity (98.10% (517/527)) was observed with *Calodium splenaecum* (KC753538; 645 bp).

#### 3.11.3. Remarks

This species, previously referred to as *Trichocephalus hepaticus* Bancroft, 1893, *Hepaticola hepatica* (Bancroft, 1893) Hall, 1916, *Capillaria hepatica* (Bancroft, 1893) Baylis, 1931, *Eucoleus hepatica* (Bancroft, 1893) López-Neyra, 1947 or with other scientific names [3], was classified by Moravec [5] into the genus *Calodium* Dujardin, 1845, which includes mammalian tissue-parasitic species such as *C. splenaecum* (Dujardin, 1843) Dujardin, 1845 in the spleen of shrews, *C. soricicola* (Yokogawa in Nishigori, 1924) Moravec, 1982 in the liver of shrews, *C. cholidicola* (Sołtys, 1952) Moravec, 1982 in the liver of shrews [5,102]. Zoonotic *C. hepaticum*, which has a cosmopolitan distribution, has been reported in over 180 mammalian species, including humans (more than 70 cases of hepatic capillariasis). However, brown rats remain the most prominent definitive hosts, exhibiting consistently high incidences of infection [103,104,105]. Recently, using *C. hepaticum* eggs isolated from the livers of brown rats in Poland, a 1025-bp long SSU rDNA nucleotide was reported (accession no. KY488184) [106]. This rDNA sequence was identical to that of the *C. hepaticum* specimens obtained in this study (LC425008) and from black rats (*Rattus rattus* (Linnaeus, 1758)) in India (PQ764976–PQ764980; 1396–1415 bp).

### 3.12. Echinocoleus yokoyamae *n. sp.*

(Syn. *Eucoleus* sp. Toda-2010a ex. badger Toda et al., 2015)

#### 3.12.1. Worm Recovery and Morphological Observation

This species was identified in the stomachs of a Japanese badger and a feral domestic cat in Wakayama Prefecture and two raccoon dogs in Saga Prefecture. With the exception of worms recovered from a Japanese badger (11 male and 11 female worms), only a few worms were collected from other parasitized animals. Additionally, the same species was detected in the stomach of one of the twenty-five wild boars collected in Wakayama Prefecture, from which five male and fourteen female worms were recovered. Thin filiform worms classified in the family Capillariidae were collected with a single row of stichocysts (a stichosome) following a short muscular esophagus. The male worm body, without caudal lateral alae, ended in two lobes with dorsolateral sickle-shaped projections, supporting two-lobed membranous bursa. Spicular sheaths had dense spines. These morphological characteristics are consistent with the definition of the genus *Echinocoleus* López-Neyra, 1947 [5].

*Echinocoleus yokoyamae* n. sp. (Nematoda: Trichocephalida: Capillariidae)

**Figure 11:**
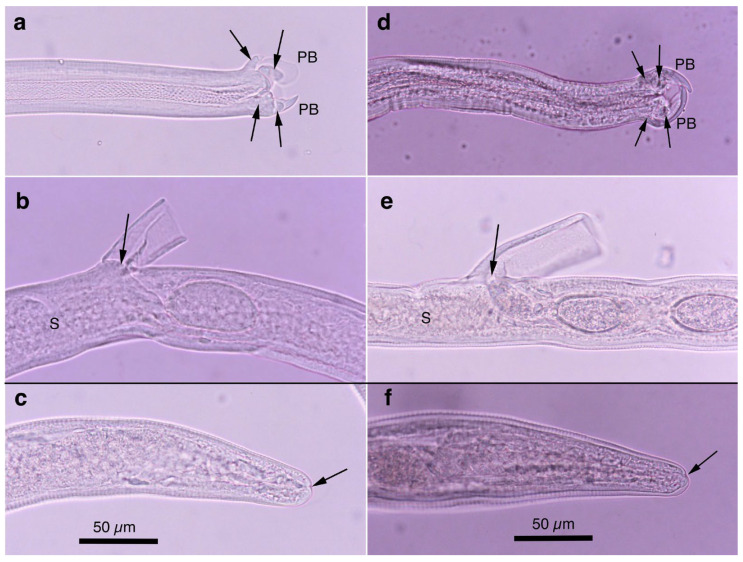
Morphology of *Echinocoleus yokoyamae* n. sp. (**a**–**c**) specimens from badgers; (**d**–**f**) specimens from wild boars. (**a**,**d**) Ventral view of caudal end of male worm, terminated in two lobes, extending to sickle-shaped thick projections (arrows on right side), which support two-lobed membranous pseudobursa (PB). In addition, a pair of digitiform ventrolateral projections at precloacal level (arrows on left side) border anterior edge of membranous bursal lobes. (**b**,**e**) Vulva (arrow) with cylindrical vulvar appendage. (**c**,**f**) Caudal end of female worm with terminal anus (arrow). All photograph at the same magnification, and scale is shown in (**c**,**f**).

**Figure 12:**
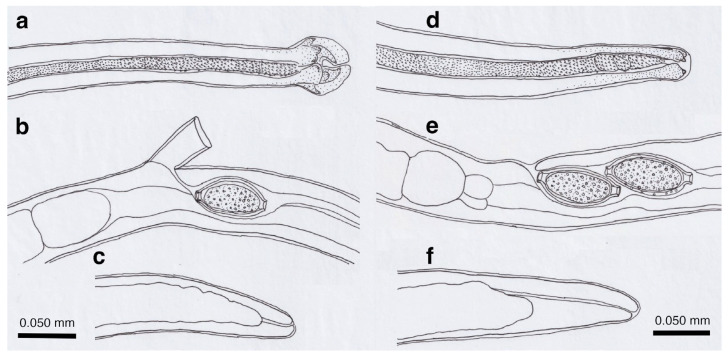
Line drawings of *Echinocoleus yokoyamae* n. sp. (**a**–**c**), and *Eucoleus kaneshiroi* n. sp. (**d**–**f**). (**a**,**d**) Ventral view of caudal end of male worms. (**b**,**e**) Vulval region of female worms, and intrauterine eggs. (**c**,**f**) Caudal end of female worm with terminal anus.

Description: Small-sized, filiform nematode with narrowed, rounded head end and indistinct oral papillae. Worm body thoroughly covered with a cuticle with fine transverse striations, gradually tapered to the anterior end, and most part of the posterior body nearly constant in width. Worms collected from the stomach of a badger were measured as follows:

Male (*n* = 9): Body length of 5.75–8.44 (7.21) mm, maximum body width of 0.035–0.050 (0.041) mm. Anterior body with a short muscular esophagus and a single row of stichocytes (stichosome), gradually increased in its body width, ended at 3.07–3.97 (3.49) mm from the head end. Ratio of the posterior body part to the anterior body part, 0.88–1.20 (1.07). The posterior body end terminated in two lobes, without caudal lateral alae. Sickle-shaped thick projections extended from each body-end lobe, supporting a two-lobed membranous bursa (Figure 11a,d and Figure 12a). A pair of digitiform ventrolateral projections at the precloacal level, bordering the anterior edge of the membranous bursal lobe (Figure 11a,d and Figure 12a). A single papilla on the ventral side of the proximal part of sickle-shaped dorsolateral projections, and another papilla on top of the precloacal projection. Spicular sheaths armed with dense spines. Spicules weakly sclerotized, observed with difficulty.

Female (*n* = 9): Female worms slightly bigger than male worms, 7.32–9.26 (8.61) mm in length, and 0.046–0.060 (0.052) mm in maximum width. Anterior body with a shortmuscular esophagus and a single row of stichocytes (stichosome), gradually increased in its body width, ended at 3.32–4.11 (3.90) mm from the anterior end. Ratio of the posterior body to the anterior body, 1.11–1.29 (1.21). Vulva at 0.027–0.079 (0.057) mm posterior to esophageal end, with a cylindrical vulvar appendage (Figure 11b,e and Figure 12b). Posterior body abruptly tapered with a blunt end and terminal anus (Figure 11c,f and Figure 12c). Bioperculated lemon-shaped eggs, measuring 0.053–0.063 (0.058) by 0.024–0.032 (0.027) mm, with smooth but minutely pitted eggshell surfaces (Figure 12b,e).

Additional measurements of worms collected from the stomach of a wild boar are shown in Supplementary Table S6.

#### 3.12.2. Molecular Characterization

The SSU rDNA sequences (1804 bp) of two *E. yokoyamae* n. sp. worms from the stomach of a badger trapped in December 2009 in Wakayama Prefecture (accession nos. LC052380 and LC052381) were nearly identical (99.83% (1801/1804) identity). These SSU rDNA sequences exhibited the highest identity with *Eucoleus kaneshiroi* n. sp. collected from the small intestine of a badger in the same locality (LC052383), with 97.45% (1756/ 1802) and 97.28% (1753/1802) identity with four indels, respectively.

#### 3.12.3. Remarks

The morphological characteristics of the newly described species were consistent with the definition of the genus *Echinocoleus* by Moravec [5], except for the well-sclerotized spicules. The type species of this genus is *E. cyanopicae* López-Neyra, 1947 described in the small intestine of Azure-winged magpies (*Cyanopica cyanus* (Pallas, 1776)) in Spain. Among the several capillariid species classified in this genus, a few mammal-parasitizing species are notable, such as *Echinocoleus hydrochoeri* (Travassos, 1916) Moravec, 1982 from the stomach and small intestine of capybara (*Hydrochoerus hydrochaeris* (Linnaeus, 1766)) from Brazil, Argentina, and Venezuela, and *Echinocoleus auritae* (Travassos, 1914) López-Neyra, 1947 from the small intestine of big-eared opossums (*Didelphis aurita* Wied-Neuwied, 1826) in Brazil [2,107]. The present new species, *E. yokoyamae* n. sp., differs from *E. hydrochoeri* in several ways, including smaller body size, larger egg size, different proportions of anterior to posterior body parts, different spicule conditions, and the presence of a pair of stumpy precloacal projections. The morphometrics of *E. auritae* are close to those of the newly described *E. yokoyamae* n. sp.; however, *E. auritae* has distinct male caudal end structures, including the absence of a pair of stumpy precloacal projections and vulvular appendages in female worms.

#### 3.12.4. Taxonomic Summary

Type host: *Meles anakuma* Temminck, 1842 (Carnivora: Mustelidae), Japanese badger.

Additional hosts: *Nyctereutes viverrinus viverrinus* (Temmink, 1838), Japanese raccoon dog (Carnivora: Canidae); *Felis silvestris catus* Linnaeus, 1758, feral domestic cat (Carnivora: Felidae); and *Sus scrofa leucomystax* Temminck, 1842, Japanese wild boar (Artiodactyla: Suidae).

Site of infection: stomach and small intestine.

Type locality: Wakayama Prefecture (Tanabe), Japan.

Additional locality: Saga Prefecture (Imari and Takeo), Japan.

Type specimens: deposited at the National Museum of Nature and Science, Tokyo. Holotype, NSMT-As4275a; paratypes, NSMT- As4263 (cat), As4275b (badger), As4249–As4250 (wild boar), and As4290–As4291 (raccoon dog).

Deposited DNA sequence: DDBJ/EMBL/GenBank accession nos. LC052380 and LC052381 (SSU rDNA).

Prevalence: one (4.8%) of 21 Japanese badgers examined, two (50%) of four Japanese raccoon dogs, one of one feral domestic cat examined, and one (4.0%) of twenty-five wild boars examined.

Infection intensity: a few, exceptionally 22 worms in the stomach of a Japanese badger.

Etiology: The species is named after Dr. Mayumi Yokoyama, a mammalogist at the Institute of Natural and Environmental Sciences, University of Hyogo, who has been our research collaborator for many years on wildlife mammalian parasites.

ZooBank number for species: urn:lsid:zoobank.org:act:31DE0977-DA95-4F0C-BD01-C9017235C9A5.

### 3.13. Eucoleus kaneshiroi *n. sp.*

(Syn. *Eucoleus* sp. Toda-2010b ex. badger Toda et al., 2015)

#### 3.13.1. Worm Recovery and Morphological Observation

This species was collected from the small intestines of four (19.0%) Japanese badgers in Wakayama Prefecture and three Japanese badgers collected in Saga Prefecture. Typically classified in the genus *Eucoleus* Dujardin, 1845, these species have a caudal end narrowed to small, rounded terminal lobes connected via a rudimentary pseudobursa with an indistinct spicule and notable spines on the spicular sheath. In Carnivora mammals, *E. aerophilus* (Creplin, 1839) Dujardin, 1845, is frequently recorded in the trachea, and *E. boehmi* (Supperer, 1953) Moravec, 1982, is found in the nasal sinuses [28,30,31,33,37,38,40,44,108,109]; however, additional species have been recorded (Supplementary Table S7). Newly isolated specimens from the small intestines of Japanese badgers were distinct from the nominal species previously reported.

*Eucoleus kaneshiroi* n. sp. (Nematoda: Trichocephalida: Capillariidae)

Description: Small-sized, filiform nematode with narrowed, rounded head end and indistinct oral papillae. Worm body entirely covered with a cuticle with fine transverse striations, gradually tapered to the anterior end, and most parts of the posterior body nearly constant in width.

**Figure 13:**
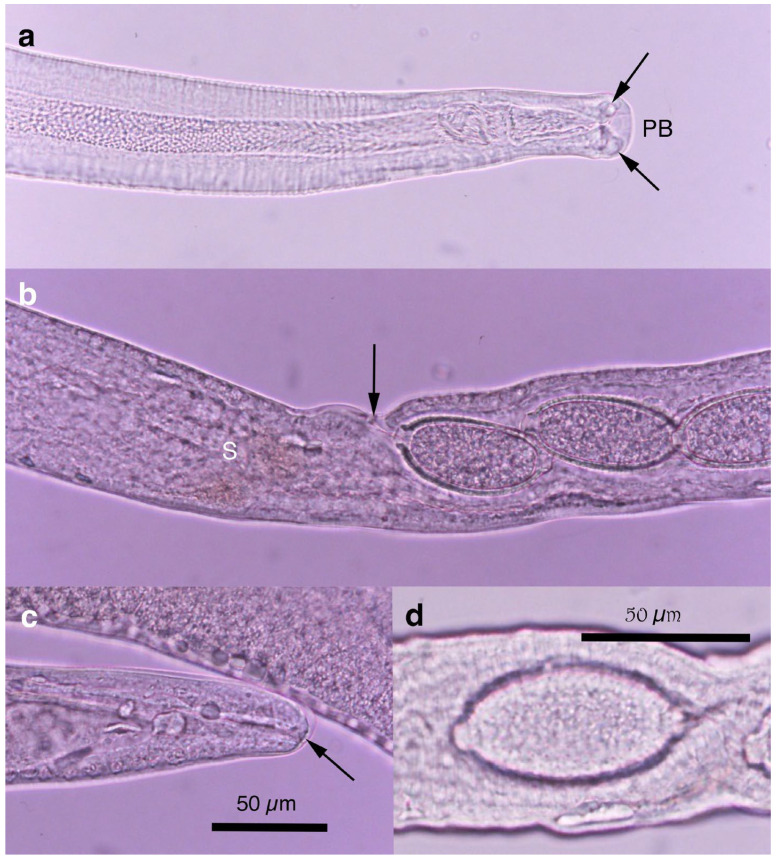
Morphology of *Eucoleus kaneshiroi* n. sp. (**a**) Ventral view of caudal end of male worm, terminated in two small lobes (arrows), supporting clear semilunar-shape membranous pseudobursa (PB). A pair of papillae, close to each other, on each caudal lobe. (**b**) Vulva (arrow) without vulvar appendage. (**c**) Caudal end of female worm with terminal anus (arrow). (**d**) Intrauterine bioperculated lemon-shaped egg, with minutely pitted eggshell surface. Photographs (**a**–**c**) at the same magnification, and scale is shown in (**c**).

Male (*n* = 17): Body length of 9.12–12.19 (10.42) mm, maximum body width of 0.044–0.059 (0.052) mm. Anterior body with a short muscular esophagus and a single row of stichocytes (stichosome), gradually increased in its body width, ending at 4.41–5.86 (5.31) mm from the anterior end. Ratio of the posterior body part to the anterior body part, 0.78–1.17 (0.97). Posterior body gradually tapered and abruptly ended into two small lobes, without caudal lateral alae, supporting a clear semilunar-shaped membranous pseudobursa (Figure 12d and Figure 13a). Two pairs of papillae close to each other on the ventral sides of the caudal lobes. Spicular sheaths with dense spines, larger spines in the proximal zone (0.074–0.132 mm in length), and fine spines in other parts. Indistinct spicule.

Female (*n* = 8): Female worms apparently bigger than male worms, 14.19–18.33 (16.05) mm in length, and 0.070–0.091 (0.077) mm in maximum width. Anterior body with a short muscular esophagus and a single row of stichocytes (stichosome), gradually increased in its width, ended at 4.49–6.41 (5.50) mm from the anterior end. The ratio of the posterior body to the anterior body, 1.49–2.19 (1.94). Vulva situated at 0.033–0.164 (0.082) mm posterior to the esophageal end, without vulvar appendages (Figure 12e and Figure 13b). Posterior body abruptly tapered with a blunt end and terminal anus (Figure 12f and Figure 13c). Bioperculated lemon-shaped eggs 0.062–0.079 (0.069) by 0.028–0.037 (0.033) mm, with minutely pitted eggshell surfaces (Figure 12e and Figure 13d).

#### 3.13.2. Molecular Characterization

The SSU rDNA sequences (1770 bp and 1804 bp) of two *E. kaneshiroi* n. sp. worms from the small intestines of two badgers trapped in December 2009 in Wakayama Prefecture were identical (accession nos. LC052382 and LC052383). These SSU rDNA sequences (represented as LC052383) showed the highest identities with *Echinocoleus yokoyamae* n. sp. (LC052380 and LC052381), with 97.45% (1756/1802) and 97.28% (1753/1802) with four indels, respectively. Similarly, with *Eucoleus boehmi* from dogs (JX456628 and JX456629) and red foxes (KC341986 and KC341987) in Italy, the identity of the *E. kaneshiroi* n. sp. SSU rDNA ranged from 97.83% (496/507) to 98.00% (538/549). The SSU rDNA sequences of *Eucoleus aerophilus* worms from the lungs of a red fox in Germany and the esophagus of red foxes in Austria (MF599385 and MW709573, respectively) showed 99.88% (1677/ 1679) identity with each other, and 95.91% (1576/1664) identity and 21 indels or 96.05% (1727/1798) identity and 22 indels with that of *E. kaneshiroi* n. sp. The SSU rDNA sequences of the present new species showed 96.55% (1568/1624) identity with 48 indels with *E. garfiai* isolated from the tongues of wild boars in Japan (LC484432 [19]).

#### 3.13.3. Remarks

The present new species, *Eucoleus kaneshiroi* n. sp., exhibits the typical morphology of the genus *Eucoleus* Dujardin, 1845, as redefined by Moravec [5], although it is located in the small intestine and not in the respiratory system, such as the trachea (e.g., *E. aerophilus* [2,3,4]), bronchioles (e.g., *E. didelphis* [110]), and nasal sinuses (e.g., *E. boehmi*, and *E. fluminensis* [2,3,111,112]), or tongue (e.g., *E. garfiai* [19,113,114]), and esophagus (*E. eberthi*, *E. procyonis* and *E. schvalovoj* [2,3,4,115,116,117]) (see Supplementary Table S7). Butterworth and Beverley-Burton [4] described several morphological traits that differentiate one species from another, for example, eggshell texture between *E. aerophilus* and *E. boehmi* (vermiculated pattern vs. finely pitted), along with distinct tissue preferences (trachea vs. nasal sinuses). Molecular differentiation presents a promising approach for the taxonomy of members of the genus *Eucoleus*, which has a simple morphology of the caudal ends in male worms. However, analysis of short SSU rDNA sequences of *E. boehmi* (JX456628, JX456629, KC341986 and KC341987 [29]; 507-592-bp sequences between 1044th and 1688th nucleotides relative to the 5′-terminus of a SSU rDNA sequence of *E. aerophilus* (MF599385), and morphologically distinct species, such as *E. aerophilus*, revealed a high degree of similarity, ranging from 99.49% (589/592) to 99.64% (547/549), with no indels. This finding suggested low interspecific variability of the SSU rDNA gene, complicating the specific identification of capillariid worms [29]. In the same 592-bp region, the SSU rDNA sequences of *E. aerophilus* (MW709573) and *E. kaneshiroi* n. sp. (LC052383) showed only 14% nucleotide substitutions and no indels, whereas 86% of 71 nucleotide substitutions and 100% of 22 indels were localized in the remaining regions of the SSU rDNA gene. *Eucoleus kaneshiroi* n. sp. is the only *Eucoleus* species reported to occur in the small intestine.

#### 3.13.4. Taxonomic Summary

Type host: *Meles anakuma* Temminck, 1842 (Carnivora: Mustelidae), Japanese badger.

Site of infection: upper small intestine.

Type locality: Wakayama Prefecture (Tanabe), Japan.

Additional locality: Saga Prefecture (Imari and Takeo), Japan.

Type specimens: deposited at the National Museum of Nature and Science, Tokyo. Holotype, NSMT-As4196 (female worm); allotype, NSMT-As4195 (male worm); paratypes, NSMT-As4194. As4197, As4198, As4208–As4210, As4221–As4223, As4243–As4248, As4252, As4262, As4270–As4274, As4285–As4289.

Deposited DNA sequence: DDBJ/EMBL/GenBank accession no. LC052382, LC052383 (SSU rDNA).

Prevalence: one (4.8%) of 21 Japanese badgers in Wakayama Prefecture, and all three Japanese badgers examined in Saga Prefecture, Japan.

Etiology: The species is named after Mr. Yoshinori Kaneshiro, a naturalist at the Nonprofit Organization Shikoku Institute of Natural History, Suzaki, Kochi, who has been our research colleague and contributed to this study as well.

ZooBank number for species: urn:lsid:zoobank.org:act:290932A4-40CB-4D73-8891-4DC497AABE27.

### 3.14. Two other Eucoleus spp.

*Eucoleus aerophilus* (Creplin, 1839) Dujardin, 1845.

(Syn. *Eucoleus* sp. Toda-2010c ex. weasel; trachea)

*Eucoleus* sp. Toda-2010d_raccoon (esophagus).

#### 3.14.1. Worm Recovery and Morphological Observation

Two *Eucoleus* specimens were obtained from the trachea of a feral alien Siberian weasel (*Mustela sibirica* Pallas, 1773) collected in December 2009 and from the esophagus of a feral alien raccoon collected in January 2010 in the Saga Prefecture. However, their morphological details were not recorded because only a few worms were collected.

#### 3.14.2. Molecular Characterization

The SSU rDNA sequences (1814 bp and 1819 bp) of *Eucoleus* worms from the trachea of a weasel and the esophagus of a raccoon were sequenced (accession nos. LC052385 and LC052384). The sequence from the weasel showed the highest identity with the SSU rDNA sequences of *E. aerophilus* from red foxes in Germany and Australia (MF599385 and MW709573), with 99.60% (1740/1747) and 99.83% (1809/1812) identity and four indels (Supplementary Table S8). Two *E. aerophilus* isolates of geographical distance but of the same origin (red foxes in Australia were introduced from Europe 150 years ago) showed a few nucleotide substitutions in the SSU rDNA sequence, with 99.83% (1746/1749) identity. An isolate from the bronchoalveolar lavage of an adult cat (MW709574) showed a 99.34% (1813/1.825) identity with *E. aerophilus* from a fox (MW709573), with four indels. An *Eucoleus* isolate from the esophagus of a raccoon (LC052384) exhibited lower identities with other *Eucoleus* isolates, the highest being 99.12% (1792/1808) with 17 indels, matching the sequence from the trachea of a weasel in this study (LC052385).

#### 3.14.3. Remarks

Although no morphological observations were made for *Eucoleus* isolates from a weasel and raccoon, molecular analyses of the SSU rDNA suggest that at least three isolates from two red foxes in Germany and Australia and a weasel in Japan (MF599385, MW709573, and LC052385) could be conspecific and classified as *E. aerophilus*. However, these isolates showed a range of nucleotide variations, including four indels, similar to the variation in *A. putorii* (four major genotypes). Another isolate, *Eucoleus* sp., from the esophagus of a raccoon, requires additional collection of specimens and morphological and molecular genetic analyses to determine its taxonomic position

### 3.15. Trichosomoides crassicauda (Bellingham, 1840) Railliet, 1895 (Trichocephalida: Trichosomoididae)

#### 3.15.1. Worm Recovery and Morphological Observation

The anterior part of this species was embedded in the epithelial lining of the urinary bladder of all nine brown rats (*Rattus norvegicus*) examined in Surabaya, Indonesia, while the posterior part was free in the lumen. An average of 6.2 female worms (range: 2–9) were collected from the six rats used only for worm recovery (not used for histological examination). Histological examination of the parasitized urinary bladder, which was conducted using the remaining three rats, showed the localization of adult worms in the transitional epithelium without a host inflammatory reaction (Supplementary Figure S5a,b).

Female filiform worms (*n* = 10) with marked transverse striation of the cuticular surface, particularly in the anterior portion between the mouth and vulva, with conspicuous serrations (Supplementary Figure S5c–e), measured 14.32–17.34 (15.69) mm in length and 0.168–0.220 (0.190) mm in maximum width. The maximum width was obtained near the posterior end and tapered gradually toward the anterior end. The esophagus consisted of short muscular and long stichosome parts, the latter of which was a series of irregularly polygonal stichocytes aligned (Supplementary Figure S5c,d), measuring 2.13–2.55 (2.36) mm in length. The ratio of the posterior part to the anterior part was 5.1–6.2 (5.7). The vulva was situated close to the end of the esophagus (stichosome), posterior to it by 0.019–0.137 (0.080) mm, without an appendage (Supplementary Figure S5e). Small-sized male worms living within the uterus were observed in two female worms (Supplementary Figure S5f), measuring 1.95 mm and 2.40 mm in length and 0.032 mm and 0.033 mm in width, respectively. In other female worms, the intrauterine eggs hindered the male worms. The posterior end of the female worm was blunt with a terminal anus (Supplementary Figure S5g). Barrel-shaped eggs measured 0.060–0.084 (0.068) mm by 0.030–0.045 (0.039) mm, containing developed larvae in its thick eggshells (Supplementary Figure S5h). Opercular plugs were not protruded.

#### 3.15.2. Molecular Characterization

The SSU rDNA sequence (1794 bp) of an adult *T. crassicauda* worm isolated from the urinary bladder of a brown rat collected in September 2017 in Indonesia was sequenced (accession no. LC425007), showing the highest identity with *Trichinella* spp. (Trichocephalida: Trichinellidae), but with up to 85% identity and 55–60 indels when the entire sequence was submitted to BLAST search.

#### 3.15.3. Remarks

This species belongs to the genus *Trichosomoides* Railliet, 1895. Marked sexual dimorphism is evident, and dwarf male worms are typically found within the uteri of female worms [78,118,119,120]. The morphology of the worms was consistent with the detailed description of the species by Hall [118]. This species is transmitted from infected to naïve rats through the direct ingestion of larvated eggs in urine or contaminated environments [78,118,119,120]. Because of this direct transmission, laboratory rats kept in some facilities have been reported to be highly infected with this species [121,122]. The pathogenicity of the nematode to the urinary bladder wall of infected rats is relatively low, even when the anterior part of the worms is embedded in the bladder urothelium, causing a mild, flat, hyperplastic response [121,122,123].

### 3.16. Phylogenetic Analysis

**Figure 14:**
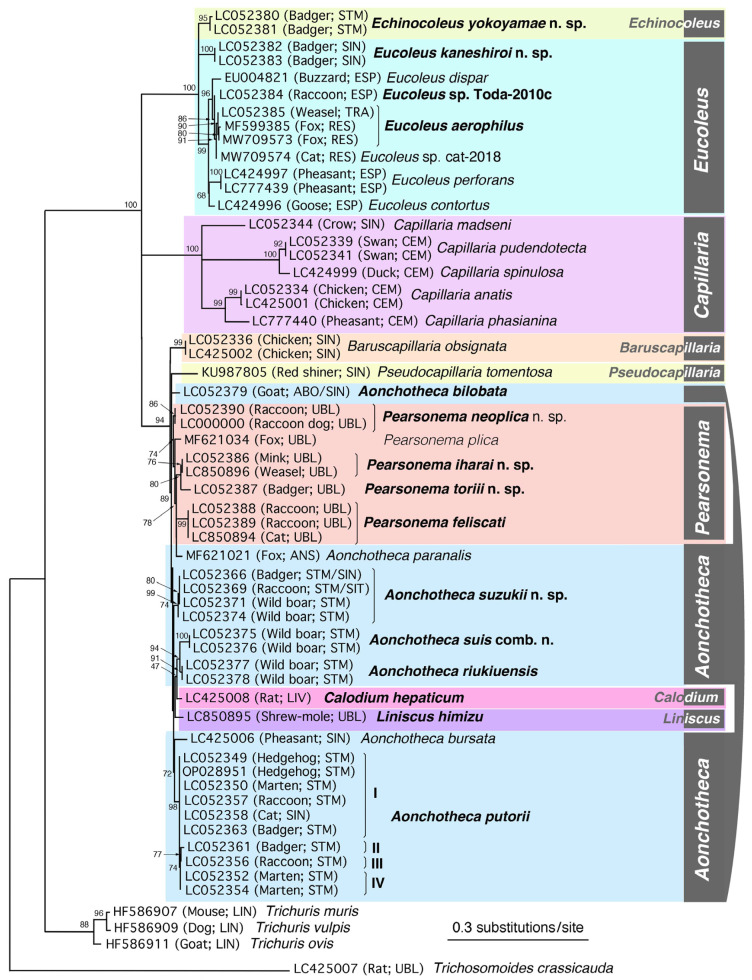
ML phylogenetic tree based on the SSU rDNA sequence. The GenBank accession number is followed by the host and isolation organ in parentheses and the species name. ABO, abomasum; CEM, cecum; ESP, esophagus; LIN, large intestine; LIV, liver; RES, respiratory system; SIN, small intestine; STM, stomach; TRA, trachea; and UBL, urinary bladder. Capillariid species reported in this study are indicated in bold letters. Sequences of isolates belonging to the same genus are placed on a single background color: *Aonchotheca*, light blue; *Baruscapillaria*, light orange; *Calodium*, pink; *Capillaria*, light violet; *Echinocoleus*, light gree; *Eucoleus*, emerald green, *Liniscus*, violet; *Pearsonema*, orange; and *Pseudocapillaria*, light yellow.

**Figure 15:**
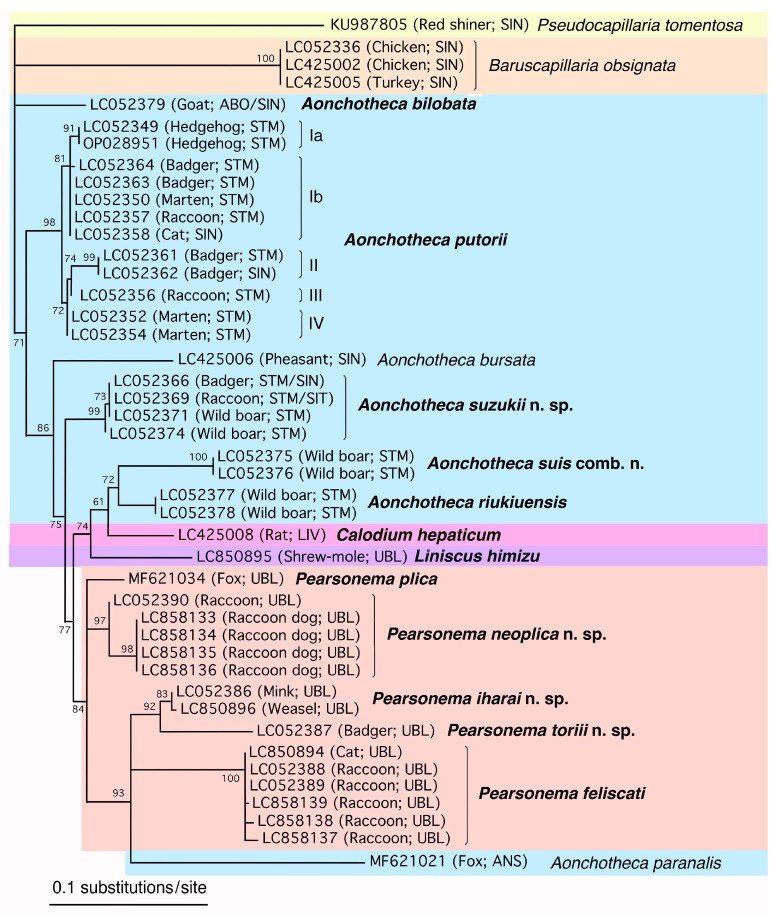
ML phylogenetic tree based on the SSU rDNA sequence, focusing on a clade of *Aonchotheca*/*Pearsonema*/*Calodium*/*Liniscus*. The labeling of each sequence, organ abbreviation, and species name are expressed as explained in the legend for Figure 14.

Nucleotide sequences of SSU rDNA, 1800–1851 bp long, were newly obtained from various capillariid worms in mammals (15 species of six genera). Including several sequences of the same gene from avian and piscine capillariids (12 species of five genera), an ML phylogenetic tree of capillariid nematodes, classified into nine genera (Trichocephalida: Capillariidae), was constructed with *Trichuris* spp. (Trichocephalida: Trichuridae) and *Trichosomoides crassicauda* (Trichocephalida: Trichosomoididae) serving as outgroups (Figure 14). Three major clades were identified: clade **A**, *Echinocoleus* and *Eucoleus* spp.; clade **B**, *Capillaria* spp.; and clade **C**, *Baruscapillaria*, *Pseudocapillaria*, *Aonchotheca*, *Pearsonema*, *Calodium* and *Liniscus* spp. Another ML phylogenetic tree was constructed using *Pseudocapillaria tomentosa* (KU987805) as an outgroup to enhance the resolution of the molecular relationships of the capillariid taxa in clade **C** (Figure 15). Members of the remaining five genera (*Bauscapillaria*, *Aonchotheca*, *Pearsonema*, *Calodium*, and *Liniscus*) were separated into several clades, irrespective of the taxonomic positions of the parasites (genus), their hosts (order), or organ specificity of the parasites. *Calodium hepaticum* (hepatic capillariid worm) and *Liniscus himizu* (shrew mole urinary bladder capillariid worm) exhibited sister relationships with *Aonchotheca* spp. in the stomach of wild boars (*A. suis* comb. n., and *A. riukiuensis*). Five *Pearsonema* spp. from the urinary bladder of Carnivora mammals formed a robust clade, however, along with a capillariid worm from the anal sac of various Carnivora mammals, originally named *Capillaria paranalis* Forstner et Geisel, 1980 [124], but recently relabeled as *Aonchotheca paranalis* by Tomczuk et al. [125]. The original description [124] highlights the lateral alae and membranous bursa of *A. paranalis* despite its inclusion in the *Pearsonema* clade.

**Figure 16:**
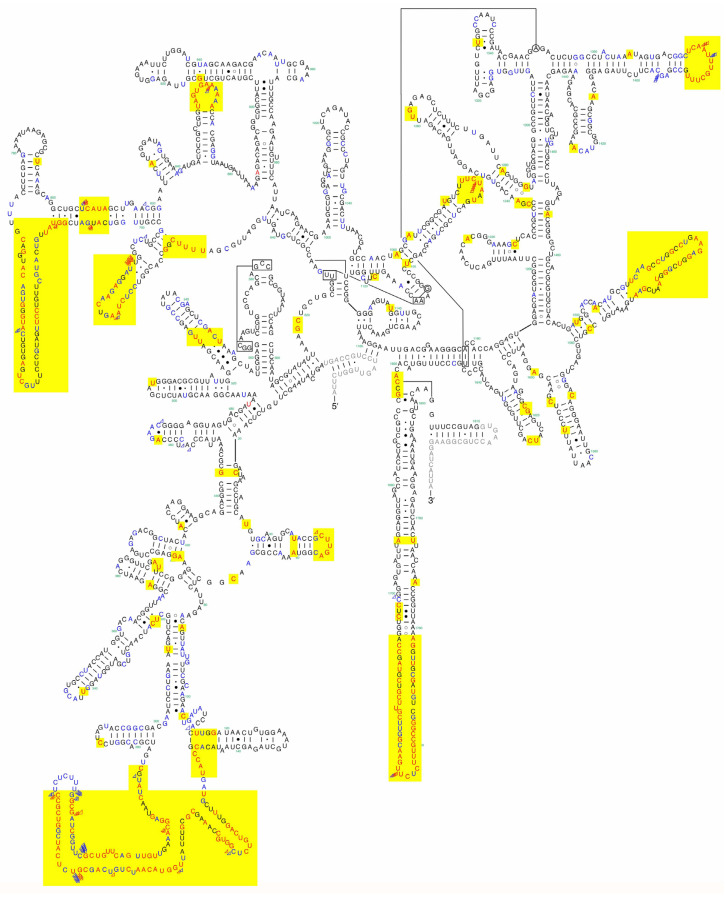
Locations of interspecific and intraspecific nucleotide changes (substitutions and indels) of 88 isolates (classified into 28 species of 9 genera) plotted on a putative SSU rDNA secondary structure of *A. putorii* (DDBJ/EMBL/GenBank accession no. LC052349). Eighty-eight sequences (isolates) are classified as *Aonchotheca* (34 sequences of 7 species), *Baruscapillaria* (5 sequences of *B. obsignata*), *Capillaria* (15 sequences of 5 species), *Echinocoleus* (2 sequences of *E. yokoyamae* n. sp.), *Eucoleus* (13 sequences of 8 species), *Pseudocapillaria* (one sequence of *P._tomentosa*), *Calodium* (one sequence of *C. hepaticum*), *Pearsonema* (16 sequence of 5 species), and *Liniscus* (one sequence of *L. himizu*). Base sites of intergeneric and interspecific nucleotide variation are expressed in red letters on a yellow background color, and those of intraspecific nucleotide variation are with blue letters. Bases expressed in black letters are highly conserved nucleotides with all capillariid isolates analyzed here.

Locations of interspecific and intraspecific nucleotide changes, including substitutions and indels, in 88 isolates classified into 28 species across nine genera are plotted on a putative SSU rDNA secondary structure of *A. putorii* (LC052349), as shown in Figure 16. Nucleotides in most stem structures were well-conserved in capillariid worms, regardless of their genera, and nucleotide changes were concentrated in certain hairpin loops (Figure 16; Supplementary Figure S6). Furthermore, the prominent locations of interspecific and intraspecific nucleotide changes differed according to the genus.

## 4. Discussion

The family Capillariidae includes more than 400 nominal species that parasitize various vertebrate hosts (fishes, amphibians, reptiles, birds, and mammals) and are distributed worldwide [2,3,4,6,64,78]. The current taxonomic system of Capillariidae, proposed by Moravec in 1982 [5] based on the morphology of the caudal end of male worms, has been provisionally assessed using a molecular approach based on SSU rDNA ([14,15,16], present study). Species differentiation is often complicated by intraspecific morphological and morphometric variations, which are vaguely attributed to physiological or immunological differences among hosts, geographic variations within the same species, or discrepancies in laboratory processing techniques. The present study demonstrated a spectrum of morphometric variations in genetically specific species. A comprehensive sampling of Capillariidae species is necessary to genetically characterize well-known species, clarify the extent of morphological variations, and confirm species identification [14,15,16].

Nucleotide changes (substitutions and indels) essential for generic or specific differentiation of capillariid nematodes occur in certain areas, depending on the genus, of the entire length of SSU rDNA (approximately 1800–1900 bp). Compiling extensive SSU rDNA sequence data for multiple capillariid species of each genus is crucial to identifying highly variable regions of taxonomic importance. This will facilitate the use of shorter partial sequences of SSU rDNA for specific differentiation in future studies. This problem is exemplified and discussed in relation to the specific differentiation of *Pearsonema* spp. and *Eucoleus* spp., and the intraspecific grouping of *A. putorii* in this study. The limitations of relying on short partial sequences (e.g., approximately 600 bp) to conclude that SSU rDNA is highly conserved among different capillariid species [29] are highlighted. Although mitochondrial DNA markers, such as *cox1*, could be a powerful tool to differentiate closely related species and intraspecific lineages [27,28,29], the implementation of specific identification for diverse capillariid nematodes based on *cox1* sequences may be challenging because of the limited applicability of universal primers.

The intraspecific diversity of the SSU rDNA sequences, ranging from 99.0% to 99.7% over a 1812-bp length, distinguished four major genotypes (I, II, III, and IV) of *A putorii* in this study. Taxonomic relationships between *A. putorii*, *A. erinacei*, and *A. mustelorum* have been debated [1,74,126,127,128,129,130,131,132,133], and *A. erinacei* and *A. mustelorum* are currently synonymous with *A. putorii* based on their morphological similarities [4]. The differentiation of the four major genotypes (I, II, III, and IV) in Japan can be partially ascribed to the introduction of alien wild mammalian hosts, such as Siberian weasels, Amur hedgehogs, common raccoons, and American minks. Notably, the full-length SSU rDNA sequences of *A. putorii* genotype Ia (1813 bp) from an alien Amur hedgehog naturalized in Japan and a hedgehog in China (LC052349 and OP028951, respectively) are identical, consistently differing from genotype Ib in two nucleotide substitutions. *Aonchotheca putorii* genotype Ib was isolated from four different hosts (a cat, a marten, badgers, and raccoons) from geographically distant locations in Japan. Three additional genetic lineages (II, III, and IV) were detected in different host mammals from various locations in Japan. Although three genotypes of *A. putorii* (Ib, III, and IV) were isolated from feral alien raccoons, the original hosts of these isolates are likely other wild mammals native to or previously naturalized in Japan, as worm recovery from feral alien raccoons is commonly low and few ([52], present study). Global genotyping of *A. putorii* from various Carnivora mammals may provide further insights into this complex species. *Aonchotheca putorii* (syn. *A. erinacei*) worms from a European hedgehog (*Erinaceus europaeus* Linnaeus, 1758) in Poland [106] (KY488186; 1024 bp) showed three nucleotide substitutions at the 1508th, 1525th, and 1536th base positions of LC052349 from sequences of all five Japanese *A. putorii* genotypes, suggesting an additional genotype closest to *A. putorii* Genotype Ia, with a 99.71% (1021/1024) partial SSU rDNA sequence identity.

The molecular characterization of capillariid worms is a powerful tool to distinguishing distinct species with the same organ or tissue preferences and higher morphological resemblance. This approach was exemplified by the recognition of *A. suzukii* n. sp. in this study. Despite minor morphological differences between *A. putorii* and *A. suzukii* n. sp., which might be considered intraspecific variations or developmental changes, SSU rDNA sequences of these two species showed 97.68–98.06% identities with five to 11 indels, indicating specific independence and euryxenous nature. Similarly, three new *Pearsonema* spp. (*P. neoplica* n. sp., *P. iharai* n. sp., and *P. toriii* n. sp.) were identified in wild canids, felids, and musterids, in addition to *P. plica* and *P. feliscati*. *Pearsonema plica* and *P. feliscati*, urinary bladder worms of canids and felids, including dogs and cats, respectively, exhibit distinct host associations. Although raccoons (*Procyon lotor*) in endemic (North America) or introduced areas (Europe) are one of the prominent definitive hosts of *P. plica* [4,66,98], feral alien raccoons in Japan harbor *P. feliscati*. Previous reports in Japan documented *P. feliscati* infections in the urinary bladders of wild raccoon dogs [79] and sporadic cases in domestic cats [99]. Newly detected *Pearsonema* spp. (*P. iharai* n. sp. and *P. toriii* n. sp.) in the urinary bladder of mustelids, such as feral alien American minks, a native Japanese weasel, and Japanese badgers in Japan exhibit distinct eggshell surface textures, a vermiculated pattern in contrast to the reticulated or punctuated eggshell surface texture patterns of *P. plica, P. neoplica* n. sp. and *P. feliscati*. Pelligra et al. [48] examined the prevalence of *Pearsonema* spp. in central and northern Italy and detected the parasites in two of 26 cats examined (7.7%), one of 83 dogs (1.2%), and 38 of 42 foxes (90.5%). Photographs show that the eggshell surfaces of their *Pearsonema* spp. [48] are distinct from *P. plica* and *P. feliscati*, suggesting a wide distribution of *Pearsonema* spp. with a vermiculated eggshell texture pattern (*P. iharai* n.sp., *P. torii* n. sp., or other species).

*Pearsonema* infections have been sporadically reported worldwide in cats and dogs [35,39,43,47,97,99]. The most commonly observed clinical signs include dysuria, pollakiuria, hematuria, pyuria, and urinary incontinence due to cystitis [35,39,97,99]. Transmission of urinary bladder worms from wild mammals to domestic cats and dogs occurs through ingestion of larvae in earthworms living in soil contaminated with urine containing *Pearsonema* eggs [36,44,45]. In the past, when urbanization was limited, a high prevalence of *Pearsonema* infections was recorded [94,95,96]. Sympatric relationships between wild and domestic mammals may significantly affect the prevalence of *Pearsonema* spp. in dogs and cats. However, further research is needed to clarify the relationship between the causative species and their pathogenicity. Similarly, other capillariid infections (capillariasis) caused by *A. putorii* (gastritis), *E. aerophilus* (respiratory distress), and *E. boehmi* (nasal discharge and sneezing) that are prevalent in wild mammals [28,30,33,38,40,46,69,91,108,109,134,135] have the same transmission pattern in small companion mammals [27,28,31,37,46,67,80,81,136,137,138,139,140,141].

As repeatedly emphasized in this study, full SSU rDNA sequencing is a powerful tool for differentiating closely related species of capillariid worms with morphological resemblances using universal eukaryotic SSU rDNA primers. Nucleotide changes in the gene, including nucleotide substitutions and indels, are localized at certain locations and vary among capillariid genera. In the absence of such molecular data, the taxonomic significance of the partial sequencing of the capillariid SSU rDNA gene remains uncertain, potentially leading to the synonymization of distinct species. Furthermore, molecular approaches to capillariid eggs with limited availability in epidemiological surveys or clinical cases of capillariasis in small companion animals (dogs and cats) enable accurate and reliable diagnosis, particularly when immature eggs are excreted in the urine or feces. Because most capillariid infections in dogs and cats may originate from wildlife reservoir host mammals, greater efforts are required to enhance understanding of capillariid diversity and the current transmission dynamics of these species among reservoir host mammals in nature ecosystems.

## 5. Conclusions—Overview of the Contents

Fine nematodes of the family *Capillariidae* parasitize various organs and tissues in fish, amphibians, reptiles, avians, and mammals. Currently classified into more than 20 genera, these nematodes are primarily distinguished based on the caudal structures of male worms. Because of their thin and fragile filiform bodies, taxonomic identification based solely on microscopic observations is challenging. Morphological and molecular analyses were conducted on 15 mammal-parasitic species belonging to the genera *Aonchotheca* (five species), *Pearsonema* (four species), *Liniscus* (one species), *Calodium* (one species), *Echinocoleus* (one species), and *Eucoleus* (three species), using specimens from various wild and domestic animals in Japan and brown rats in Indonesia. *Aonchotheca putorii*, commonly found in the stomachs of members of Canidae, Felidae, Mustelidae, and Erinaceidae, exhibited substantial genetic and morphological diversity, consisting of different morphotypes and at least four major genotypes based on the small subunit ribosomal RNA gene (SSU rDNA). Another *Aonchotheca* species, *Aonchotheca suzukii* n. sp., was identified in the stomach of badgers, raccoon dogs, feral alien raccoons, and wild boars. This new species is clearly differentiated from *Aonchotheca suis* (Yamaguti, 1943) n. comb. (syn. *Capillaria suis*) and *Aonchotheca riukiuensis*, which commonly infect the stomach of wild boars, and *A. putorii*, which parasitizes Carnivora mammals. These distinctions are based on morphological and molecular genetic traits. Urinary bladder capillariid worms from native raccoon dogs and feral alien raccoons closely resembled *Pearsonema plica*, a well-known capillariid species found in the urinary bladder of caniids worldwide. Similarities included a reticulated eggshell surface texture and triangular caudal bursa in male worms. However, the newly identified isolate, *Pearsonema neoplica* n. sp., exhibited a shorter spicule length (<2.0 mm vs. >2.0 mm) and a lack of cylindrical vulvar appendages, distinguishing it from *P. plica*. The molecular analysis further supported the classification of this new species. Urinary bladder capillariid worms recovered from domestic cats and feral alien raccoons were identified as *Pearsonema feliscati*, characterized by a punctuated eggshell surface texture. In addition to *P. neoplica* n. sp. and *P. feliscati*, new isolates with vermiculated eggshell surface textures, *Pearsonema iharai* n. sp. and *P. toriii* n. sp., are described using specimens from the urinary bladders of musterids, including feral alien American minks, a native Japanese weasel, and badgers. These newly identified species lacked digitiform projections on the caudal dorsolateral lobes in male worms. This characteristic diverged from the generic definition of *Pearsonema* and instead resembled the male caudal morphology of the genus *Liniscus* found in the urinary bladder of small mammals (rodents, soricids, and talpids). In particular, the new mustelid-parasitic capillariid species formed a clade with *P. plica*, *P. neoplica* n. sp., and *P. feliscati* while remaining distinct from *Liniscus himizu*, a species found in the urinary bladder of the Japanese shrew mole. New capillariid species from the Japanese badger were also identified, including *Echinocoleus yokoyamae* n. sp. in the stomach and *Eucoleus kaneshiroi* n. sp. in the small intestine. As demonstrated in this study, nearly complete SSU rDNA sequencing is a powerful tool for differentiating closely related species and clarifying the phylogenetic relationships among morphologically similar capillariid worms. Nucleotide changes, including substitutions and insertions or deletions (indels), occur unevenly throughout the gene and are localized in certain areas, depending on the genus. Consequently, arbitrarily selecting partial gene sequences may yield insufficient information and risk synonymizing distinct capillariid species. Additionally, most capillariid worms detected in dogs and cats are suspected to be shared with their respective wildlife reservoir host mammals. Therefore, molecular characterization, combined with the microscopic observation of these parasites in wildlife mammals, provides a robust framework for accurate species identification, reliable classification, and epidemiological assessment.

## Figures and Tables

**Table 1:** Measurements of *Aonchotheca putorii* (types A and B) from different mammalian species.

Worm type	Type A	Type B	–^a^	–	–	–	–
Host species	*Martes melampus*; *Mustela sibirica*; *Felis silvastris catus*; *Erinaceus amurensis*	*Martes melampus*; *Mustela sibirica*; *Procyon lotor*	*Lutra lutra*; *Mustela* spp.; *Neogale vison* (syn. *Neovison vison*)*; Martes foina*	*Neogale vison* (syn. *Neovison vison*)	*Neogale vison* (syn. *Neovison vison*)	*Erinaceus europaeus*	*Procyon lotor*
Location	stomach, small intestine	stomach, small intestine	esophagus, stomach, small intestine	stomach	small intestine	stomach	stomach
Locality	Japan (Aomori, Akita, Shizuoka, Wakayama, Kochi)	Japan (Aomori, Akita, Kochi, Saga, Nagasaki)	Europe, North America	Canada (Ontario)	Canada (Ontario)	Canada (Ontario)	Canada (Ontario)
Reference	Present study	Present study	[2]	[4]	[4]	[4]	[4]
**Male worms**	(*n* = 44)	(*n* = 14)	–	(*n* = 10)	(*n* = 10)	(*n* = 10)	(*n* = 10)
Worm length	4.47–7.56 (6.10)	4.47–6.74 (5.52)	5.4–8.0	5.6–8.0 (6.8)	6.9–9.6 (8.4)	5.1–6.9 (5.9)	4.5–7.1 (5.8)
Max. worm width	0.037–0.070 (0.047)	0.042–0.055 (0.046)	0.058–0.068	0.043–0.053 (0.046)	0.048–0.060 (0.053)	0.038–0.048 (0.040)	0.035–0.048 (0.041)
Esophagus length	2.25–3.78 (3.01)	2.17–3.01 (2.63)	2.41–2.55	2.6–3.5 (3.1)	3.2–3.9 (3.5)	2.2–3.5 (2.8)	2.3–3.4 (2.9)
P/A proportion ^c^	0.80–1.29 (1.00)	0.91–1.41 (0.96)	1.25–2.17	–	–	–	–
Spicule length	0.301–0.482 (0.363)	0.203–0.247 (0.216)	0.149–0.168	0.241–0.368 (0.323)	0.240–0.357 (0.288)	0.243–0.351 (0.309)	0.194–0.351 (0.242)
**Female worms**	(*n* = 70) ^b^		–	(*n* = 10)	(*n* = 10)	(*n* = 10)	(*n* = 10)
Worm length	5.32–12.16 (8.54)		9.4–15.0	7.1–11.8 (9.7)	9.6–14.1 (12.1)	8.9–12.4 (10.9)	5.4–11.1 (9.1)
Max. worm width	0.043–0.112 (0.067)		0.068–0.088	0.053–0.066 (0.058)	0.065–0.075 (0.072)	0.049–0.069 (0.056)	0.043–0.065 (0.058)
Esophagus length	2.19–4.33 (3.27)		3.02–3.59	2.6–3.9 (3.4)	3.1–4.2 (3.9)	3.3–4.1 (3.7)	2.7–4.3 (3.7)
P/A proportion ^c^	1.17–1.95 (1.60)		2.10–3.17	–	–	–	–
Vulva from the esophageal end	0.041–0.142 (0.081)		–	–	–	–	–
Egg length	0.053–0.070 (0062)		0.064–0.072	0.044–0.066 (0.061)	0.059–0.068 (0.064)	0.055–0.065 (0.059)	0.058–0.066 (0.063)
Egg width	0.022–0.036 (0028)		0.028–0.032	0.021–0.026 (0.024)	0.025–0.028 (0.026)	0.021–0.025 (0.024)	0.020–0.026 (0.024)

^a^ Data not available. ^b^ Types of worms undetermined. ^c^ P/A proportion: ratio of the posterior body to the anterior body.

**Table 2:** Comparison of nucleotide variations in the SSU rDNA sequences of *Aonchotheca putorii* from different mammalian species in Japan.

Genotype	Morphotype	Host	Locality	GenBank Accession No.	Relative Position of Nucleotide Where Nucleotide Changes Occur ^a^																																		
					108	167	171	172	174	210	212	213	214	218	220	224	225	226	229	—229/230—						230	234	859	1506	1545	1546	1547	1556	1701	1709	1712	1719	1736	1737
Ia	A	*Erinaceus amurensis*	Shizuoka	LC052349	T	G	C	G	T	C	C	A	T	T	G	C	T	C	T	–	–	–	–	–	–	G	A	G	A	A	A	T	C	C	C	T	T	T	T
	n.d. ^b^	*Erinaceus europaeus* ^c^	Beijing, China	OP028951	▪	▪	▪	▪	▪	▪	▪	▪	▪	▪	▪	▪	▪	▪	▪	▪	▪	▪	▪	▪	▪	▪	▪	▪	▪	▪	▪	▪	▪	▪	▪	▪	▪	▪	▪
Ib	A	*Martes m. melampus*	Kochi	LC052350	▪	▪	▪	▪	▪	▪	▪	▪	▪	C	▪	▪	▪	▪	▪	▪	▪	▪	▪	▪	▪	▪	▪	▪	▪	▪	▪	▪	▪	T	▪	▪	▪	▪	▪
	A	*Meles anakuma*	Wakayama	LC052363	▪	▪	▪	▪	▪	▪	▪	▪	▪	C	▪	▪	▪	▪	▪	▪	▪	▪	▪	▪	▪	▪	▪	▪	▪	▪	▪	▪	▪	T	▪	▪	▪	▪	▪
	n.d.	*Felis silvestris catus*	Wakayama	LC052358–LC052360	▪	▪	▪	▪	▪	▪	▪	▪	▪	C	▪	▪	▪	▪	▪	▪	▪	▪	▪	▪	▪	▪	▪	▪	▪	▪	▪	▪	▪	T	▪	▪	▪	▪	▪
	n.d.	*Meles anakuma*	Kochi	LC052364	▪	▪	▪	▪	▪	▪	▪	▪	▪	C	▪	▪	▪	▪	▪	▪	▪	▪	▪	▪	▪	▪	▪	▪	▪	▪	▪	▪	T	T	▪	▪	▪	▪	▪
	n.d.	*Procyon lotor*	Saga	LC052356	▪	▪	▪	▪	▪	▪	▪	▪	▪	C	▪	▪	▪	▪	▪	▪	▪	▪	▪	▪	▪	▪	▪	▪	▪	▪	▪	▪	▪	T	▪	▪	▪	▪	▪
	n.d.	*Procyon lotor*	Nagasaki	LC052357	▪	▪	▪	▪	▪	▪	▪	▪	▪	C	▪	▪	▪	▪	▪	▪	▪	▪	▪	▪	▪	▪	▪	▪	▪	▪	▪	▪	▪	T	▪	▪	▪	▪	▪
II	n.d.	*Meles anakuma*	Shiga	LC052361	A	▪	T	T	C	T	T	G	C	C	▪	G	▪	▪	▪	▪	▪	▪	▪	▪	▪	▪	▪	▪	G	▪	▪	▪	▪	T	T	C	C	G	–
	n.d.	*Meles anakuma*	Shiga	LC052362	A	▪	T	T	C	T	T	G	C	C	▪	G	▪	▪	▪	▪	▪	▪	▪	▪	▪	▪	▪	▪	G	▪	▪	▪	▪	T	T	C	C	G	–
III	B	*Procyon lotor*	Saga	LC052356	▪	T	T	▪	▪	T	G	▪	▪	C	A	▪	G	T	G	▪	▪	▪	▪	▪	G	T	T	C	▪	▪	▪	▪	▪	T	T	▪	C	G	–
	AB	*Meles anakuma*	Wakayama	LC052365	▪	T	T	▪	▪	T	G	▪	▪	C	A	▪	G	T	G	▪	▪	▪	▪	▪	G	T	T	C	▪	T	T	A	▪						
IV	B	*Martes m. melampus*	Kochi	LC052352	▪	▪	T	▪	▪	T	T	▪	▪	C	A	▪	▪	T	▪	T	G	C	G	G	G	T	T	C	▪	▪	▪	▪	▪	T	C	▪	C	G	–
	n.d.	*Martes m. melampus*	Kyoto	LC052353	▪	▪	T	▪	▪	T	T	▪	▪	C	A	▪	▪	T	▪	T	G	C	G	G	G	T	T	C	▪	▪	▪	▪	▪	T	C	▪	C	G	–
	B	*Martes m. melampus*	Wakayama	LC052354	▪	▪	T	▪	▪	T	T	▪	▪	C	A	▪	▪	T	▪	T	G	C	G	G	G	T	T	C	▪	▪	▪	▪	▪	T	C	▪	C	G	–
	B	*Procyon lotor*	Saga	LC052355	▪	▪	T	▪	▪	T	T	▪	▪	C	A	▪	▪	T	▪	T	G	C	G	G	G	T	T	C	▪	▪	▪	▪	▪	T	C	▪	C	G	–

^a^ Relative position of the 5′-terminus of the SSU rDNA sequence of *A. putorii* isolated from a feral alien Amur hedgehog in Shizuoka, Japan (DDBJ/EMBL/GenBank accession no. LC052349). ^b^ No data available. ^c^ Deng et al. [32] wrote that adult parasites were collected from the small intestine of dead European hedgehogs (*Erinaceus europaeus* L.) originated from Beijing, China. It is uncertain why European hedgehogs native to Europe came from Beijing.

**Table 3:** Pairwise comparison of nucleotide variations in the SSU rDNA sequences of different genotypes of *Aonchotheca putorii* from wild mammalian hosts in Japan ^a^.

Genotype	GenBank Accession No.	*Aonchotheca putorii* Genotype					
		Type Ia	Type Ib	Type II	Type IIIa	Type IIIb	Type IV
Type Ia	LC052349 (1813 bp)		2	16 (1)	14 (2)	14 (1)	12 (7)
Type Ib	LC052350 (1813 bp)	99.89		14 (1)	12 (2)	13 (1)	10 (7)
Type II	LC052361 (1812 bp)	99.12	99.23		16 (1)	18 (1)	12 (6)
Type IIIa	LC052356 (1813 bp)	99.23	99.34	99.12		3	4 (5)
Type IIIb	LC052365 (1590 bp)	99.12	99.18	98.87	99.81		6 (5)
Type IV	LC052352 (1818 bp)	99.34	99.45	99.34	99.79	99.62	

^a^ Number of nucleotide substitutions (number of indel sites) in upper diagonal and nucleotide identity (%) in lower diagonal.

**Table 4:** Measurements of *Aonchotheca suzukii* n. sp. from different mammalian species.

	*Aonchotheca suzukii* n. sp.				*Aonchotheca putorii*	
					Morphotype A	Morphotype B
Host species	*Procyon lotor*	*Nyctereutes procyonoides*	*Meles anakuma*	*Sus scrofa* *leucomystax*	*Martes melampus*; *Mustela sibirica*; *Felis silvastris catus*; *Erinaceus amurensis*	*Martes melampus*; *Mustela sibirica*; *Procyon lotor*
Locality	Japan (Saga, Nagasaki)	Japan (Gunma, Wakayama)	Japan (Kyoto, Wakayama, Saga)	Japan (Hyogo, Wakayama)	Japan (Aomori, Akita, Shizuoka, Wakayama, Kochi)	Japan (Aomori, Akita, Kochi, Saga, Nagasakai)
Male worms	(*n* = 4)	(*n* = 2)	(*n* = 2)	(*n* = 2)	(*n* = 44)	(*n* = 14)
Worm length	5.78–6.66 (6.13)	6.11–6.22	4.52–4.60	6.90–7.59	4.47–7.56 (6.10)	4.47–6.74 (5.52)
Max. worm width	0.042–0.050 (0.045)	0.040–0.042	0.032–0.034	0.050–0.061	0.037–0.070 (0.047)	0.042–0.055 (0.046)
Esophagus length	2.74–3.12 (2.93)	2.85–2.90	2.30–2.52	3.21–3.70	2.25–3.78 (3.01)	2.17–3.01 (2.63)
P/A proportion ^a^	1.05–1.13 (1.09)	1.10–1.18	0.83–0.97	1.05–1.15	0.80–1.29 (1.003)	0.91–1.41 (0.955)
Spicule length	0.370–0.455 (0.410)	0.364–0.381	0.240–0.357 (0.288)	0.390–0.460	0.301–0.482 (0.363)	0.203–0.247 (0.216)
Female worms	(*n* = 5)	(*n* = 5)	(*n* = 17)	(*n* = 6)		(*n* = 70) ^b^
Worm length	6.82–9.23 (8.26)	8.49–10.16 (9.23)	5.56–7.45 (6.24)	8.96–9.73 (9.22)		5.32–12.16 (8.54)
Max. worm width	0.049–0.057 (0.052)	0.0503–0.065 (0.056)	0.039–0.054 (0.047)	0.053–0.061 (0.056)		0.043–0.112 (0.067)
Esophagus length	2.44–3.29 (2.99)	2.82–3.07 (2.94)	2.16–2.79 (2.41)	2.66–3.12 (2.90)		2.19–4.33 (3.27)
P/A proportion ^a^	1.67–1.86 (1.77)	1.92–2.37 (2.14)	1.41–1.74 (1.59)	1.92–2.39 (2.18)		1.17–1.95 (1.60)
Vulva from the esophageal end	0.068–0.186 (0.135)	0.060–0.121 (0.087)	0.041–0.082 (0060)	0.082–0.132 (0.110)		0.041–0.142 (0.081)
Egg length	0.058–0.064 (0.060)	0.056–0.063 (0.060)	0.053–0.064 (0.058)	0.054–0.066 (0.059)		0.053–0.070 (0.062)
Egg width	0.023–0.029 (0.026)	0.024–0.028 (0.026)	0.021–0.030 (0.025)	0.024–0.027 (0.025)		0.022–0.036 (0.028)

^a^ P/A proportion: ratio of the posterior body to the anterior body. ^b^ Types of worms undetermined.

**Table 5:** Prevalence of capillariid worms from the urinary bladder of wild mammals in Japan.

Host	Locality	Date	No. of Animals Examined	No. of Positive Animals with Parasites		Parasite Species
*Procyon lotor*	Hyogo Pref.	Aug. 2009–Jul. 2010	154	9 (5.8%) ^a^	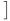	*Pearsonema neoplica* n. sp. *Pearsonema feliscati*
	Wakayama Pref.	Feb. 2015–Jul. 2017	477	26 (5.5%) ^a^		
*Nyctereutes procyonoides viverrinus*	Wayakama Pref.	Feb. 2015–Jan. 2016	32	14 (43.8%)		*Pearsonema neoplica* n. sp.
*Neogale vison*	Fukushima Pref.	Aug. 2010–Oct. 2010	76	31 (40.8%)		*Pearsonema iharai* n. sp.
*Meles anakuma*	Wakayama Pref.	Mar. 2010–Oct. 2010	29	13 (44.8%)		*Pearsonema toriii* n. sp.

^a^ Two parasite species were not differentiated.

**Table 6:** Measurements of *Pearsonema* spp. from the urinary bladder of Carnivora mammals (expressed in mm).

Species	*Pearsonema neoplica* n. sp.		*Pearsonema plica* (Rudolphi, 1819)				*Pearsonema feliscati* (Bellingham, 1845)	
	(with triangular bursa / no vulval appendage)	(without triangular bursa / no vulval appendage)^a^	(with triangular bursa / cylindrical vulval appendage)		(with triangular bursa / no vulval appendage)		(without triangular bursa / no vulval appendage)	
Host	*Nyctereutes procyonoides viverrinus*	*Nyctereutes procyonoides viverrinus*	*Vulpes vulpes*	*Procyon lotor*	*Meles meles*	*Vulpes* spp.	*Procyon lotor*	*Felis* spp.
Locality	Japan (Wakayama)	Japan (Wakayama)	Canada (Ontario)		Europe		Japan (Wakayama)	–
Reference	Present study	Present study	[4]		Rukhlyadev (1948) in [3]		Present study	[1]
Male worms	(*n* = 4)	(*n* = 8)	(*n* = 15)	(*n* = 15)	(*n* = ?)	(*n* = ?)	(*n* = 4)	(*n* = ?)
Worm length	19.29–23.85 (20.65)	16.76–22.21 (20.60)	28.6–53.3 (39.9)	16.7–31.2 (22.6)	21.06–32.94	27.00–37.80	20.54–24.23 (22.27)	25.5
Max. worm width	0.052–0.061 (0.056)	0.044–0.057 (0.051)	0.055–0.070 (0.060)	0.031–0.048 (0.041)	0.044–0.060	0.052–0.060	0.046–0.055 (0.050)	0.032–0.064
Esophagus length	4.97–7.39 (6.25)	5.23–7.47 (6.53)	7.7–11.7 (9.3)	3.5–6.8 (5.4)	6.66–7.92	9.00–10.26	4.06–7.17 (6.04)	6.7
P/A proportion ^b^	1.61–3.80 (2.41)	1.95–2.39 (2.17)	– (3.29)	– (3.19)	– ^c^	–	2.32–4.33 (2.85)	3
Spicule length	1.55–1.89 (1.78)	1.47–1.80 (1.61)	3.4–5.2 (4.5)	2.3–3.5 (2.9)	2.80–3.85	2.83–3.87	2.22–2.46 (2.33)	2.5
Female worms	(*n* = 7)	(*n* = 9)	(*n* = 15)	(*n* = 15)	(*n* = ?)	(*n* = ?)	(*n* = 10)	(*n* = ?)
Worm length	20.45–30.63 (26.34)	19.96–26.69 (21.80)	29.4–52.2 (42.7)	17.6–44.9 (25.9)	27.9–36.0	28.44–29.34	13.32–34.70 (22.91)	28.6–31.9
Max. worm width	0.070–0.103 (0.082)	0.067–0.087 (0.079)	0.089–0.114 (0.100)	0.060–0.109 (0.070)	0.081–0.085	0.088	0.047–0.108 (0.081)	0.032–0.144
Esophagus length	6.59–8.31 (7.79)	7.15–9.84 (8.10)	6.9–11.9 (9.9)	4.4–8.4 (6.6)	8.82–9.90	10.08–10.98	5.22–13.48 (9.22)	10.2–10.8
P/A proportion ^b^	2.06–2.97 (2.39)	1.48–2.02 (1.73)	– (3.31)	– (2.92)	–	–	1.21–2.08 (1.52)	2
Vulva from the esophageal end	0.052–0.235 (0.107)	0.058–0.208 (0.128)	–	–	–	–	0.073–0.525 (0.248)	0.034–0.544
Egg length	0.060–0.068 (0.064)	0.064–0.073 (0.068)	0.058–0.071 (0.065)	0.059–0.074 (0.064)	0.062–0.068	0.062–0.065	0.057–0.078 (0.063)	0.051–0.062
Egg width	0.027–0.030 (0.028)	0.026–0.029 (0.028)	0.025–0.031 (0.028)	0.023–0.028 (0.026)	0.029–0.031	0.026–0.028	0.022–0.031 (0.026)	0.024–0.032

^a^ Exceptional worms without triangular bursa (see text). ^b^ P/A proportion: ratio of the posterior body to the anterior body. ^c^ No available data.

**Table 7:** Comparison of nucleotide variations in the SSU rDNA sequences of *Pearsonema* spp. from different mammalian species in Japan.

Parasite ^a^			Host		GenBank Accession No.			Sequence Length (bp)		Relative position of nucleotide where nucleotide changes occur ^b^																									
										52	54	61	104	109	147	148	150	151	153	154	164	166	167	—167/168—				168	169	172	173	174	198	205	206
*P. neoplica* n. sp.			Raccoon dog		LC858133			1831		C	T	A	G	A	A	C	C	C	C	G	A	T	A	–	–		–	T	G	G	G	T	A	A	G
*P. neoplica* n. sp.			Raccoon dog		LC858134			1808		•	•	•	•	•	•	•	•	•	•	•	•	•	•	•	•		•	•	•	•	•	•	•	•	•
*P. neoplica* n. sp.			Raccoon dog		LC858135			1808		•	•	•	•	•	•	•	•	•	•	•	•	•	•	•	•		•	•	•	•	•	•	•	•	•
*P. neoplica* n. sp.			Raccoon dog		LC858136			1808		•	•	•	•	•	•	•	•	•	•	•	•	•	•	•	•		•	•	•	•	•	•	•	•	•
*P. neoplica* n. sp.			Raccoon		LC052390			1808		•	•	•	•	•	•	•	•	•	•	•	•	•	•	•	•		•	•	•	•	•	•	•	•	•
*P. plica*			Red foxa		MF621034			1743		•	•	•	•	•	•	•	G	T	•	•	•	C	T	•	•		•	•	•	•	•	•	•	G	•
*P. iharai* n. sp.			Mink		LC052386			1810		•	•	•	•	•	•	•	•	T	•	•	•	G	T	•	•		•	•	T	•	–	•	•	G	•
*P. iharai* n. sp.			Weasel		LC850896			1816		•	•	•	•	•	•	•	•	T	•	•	•	G	T	•	•		•	•	T	•	–	C	•	G	•
*P. toriii* n. sp.			Badger		LC052387			1813		G	•	T	A	G	G	T	•	T	T	•	•	G	C	•	•		•	•	•	•	•	C	G	•	•
*P. feliscati*			Raccoon		LC052388			1824		•	G	•	A	•	•	•	•	•	•	•	G	C	T	T	T		T	•	•	T	•	C	•	G	•
*P. feliscati*			Raccoon		LC052389			1827		•	G	•	A	•	•	•	•	•	•	•	G	C	T	T	T		T	•	•	T	•	C	•	G	•
*P. feliscati*			Raccoon		LC858137			1850		•	G	•	A	•	•	•	•	•	•	•	G	C	T	T	T		T	•	•	T	•	C	•	G	•
*P. feliscati*			Raccoon		LC858138			1850		•	G	•	A	•	•	•	•	•	•	•	G	C	T	T	T		T	•	•	T	•	C	•	G	•
*P. feliscati*			Raccoon		LC858139			1850		•	G	•	A	•	•	•	•	•	•	•	G	C	T	T	T		T	•	•	T	•	C	•	G	•
*P. feliscati*			Cat		LC850894			1827		•	G	•	A	•	•	•	•	•	•	•	G	C	T	T	T		T	•	•	T	•	C	•	G	•
*Liniscus himizu*			Shrew mole		LC850895			1798		•	•	•	A	•	•	•	•	•	•	A	•	•	T	G	•		•	C	T	•	T	•	•	•	A
Relative position of nucleotide where nucleotide changes occur ^b^																																			
207	209	210	211	212	213	216	217	218	219	220	221	222	223	224	225	226	—226/227—								227		228	—228/229—							229
C	T	A	T	G	G	G	G	C	T	A	C	C	T	T	G	C	–	–	–		–	–	–	–	G		T	–	–	–	–	–		–	G
•	•	•	•	•	•	•	•	•	•	•	•	•	•	•	•	•	•	•	•		•	•	•	•	•		•	•	•	•	•	•		•	•
•	•	•	•	•	•	•	•	•	•	•	•	•	•	•	•	•	•	•	•		•	•	•	•	•		•	•	•	•	•	•		•	•
•	•	•	•	•	•	•	•	•	•	•	•	•	•	•	•	•	•	•	•		•	•	•	•	•		•	•	•	•	•	•		•	•
•	•	•	•	•	•	•	•	•	•	•	•	•	•	•	•	•	•	•	•		•	•	•	•	•		•	•	•	•	•	•		•	•
T	•	•	•	•	•	•	•	•	•	•	•	G	•	•	•	•	•	•	•		•	•	•	•	•		•	•	•	•	•	•		•	•
•	•	G	•	T	•	•	•	T	•	G	•	•	•	G	C	T	•	•	•		•	•	•	A	A		•	G	G	G	C	A		•	A
•	•	G	•	T	•	•	•	T	•	G	•	•	•	A	C	T	T	C	T		A	G	T	A	T		•	G	G	G	C	A		•	A
•	C	G	•	T	•	•	•	T	•	G	G	•	•	G	C	T	•	•	•		•	•	•	A	A		•	G	G	C	C	A		•	A
•	•	G	•	T	•	T	A-	T	•	G	G	T	G	C	C	G	C	T	A		A	G	C	A	•		C	G	G	T	A	G		C	A
•	•	G	•	T	•	T	A	T	•	G	G	T	G	C	C	G	C	T	A		A	G	C	A	•		C	G	G	T	A	G		C	A
•	•	G	•	T	•	T	A	T	•	G	G	T	G	C	C	G	C	T	A		A	G	C	A	•		C	G	G	T	A	G		C	A
•	•	G	•	T	•	T	A	T	•	G	G	T	G	C	C	G	C	T	A		A	G	C	A	•		C	G	G	T	A	G		C	A
•	•	G	•	T	•	T	A	T	•	G	G	T	G	C	C	G	C	T	A		A	G	C	A	•		C	G	G	T	A	G		C	A
•	•	G	•	T	•	T	A	T	•	G	G	T	G	C	C	G	C	T	A		A	G	C	A	•		C	G	G	T	A	G		C	A
•	•	T	A	T	A	•	•	–	–	–	–	–	•	•	C	A	•	•	•		•	•	•	•	A		•	•	•	•	•	•		•	A
Relative position of nucleotide where nucleotide changes occur ^b^																																			
230	239	245	266	267	290	305	319	412	493	522	534	614	667	674	685	686	686/687	718	719		724	737	738	740	749	753	754	756	757	853	855	860	861	912	1075
G	C	A	A	T	C	G	A	C	T	T	T	C	T	A	T	C	–	G	C		C	T	G	C	T	T	G	G	A	G	G	A	A	T	T
•	•	•	•	•	•	•	•	•	•	•	•	•	•	•	•	•	•	•	•		•	•	•	•	•	•	•	•	•	•	•	•	•	•	•
•	•	•	•	•	•	•	•	•	•	•	•	•	•	•	•	•	•	•	•		•	•	•	•	•	•	•	•	•	•	•	•	•	•	•
•	•	•	•	•	•	•	•	•	•	•	•	•	•	•	•	•	•	•	•		•	•	•	•	•	•	•	•	•	•	•	•	•	•	•
•	•	•	•	•	•	•	•	•	•	•	•	•	•	•	•	•	•	•	•		•	•	•	•	•	•	•	•	•	•	•	•	•	•	•
•	•	•	T	•	T	•	•	•	•	•	C	•	•	•	•	•	•	•	•		•	•	•	•	•	•	•	•	•	•	•	T	•	C	C
–	•	•	A	•	•	•	•	•	•	•	C	•	C	G	•	•	•	•	•		•	•	A	•	C	•	•	•	•	•	•	–	G	•	C
–	•	•	A	•	•	•	•	•	•	•	C	•	C	G	•	•	•	•	•		•	•	A	•	C	•	•	•	•	•	•	–	G	•	C
A	•	G	A	•	•	•	•	•	•	•	C	•	C	G	•	•	•	•	•		•	•	•	•	C	•	•	•	•	•	•	•	G	•	C
•	•	•	•	C	•	•	•	•	C	C	C	•	C	G	C	T	G	•	•		T	•	•	•	•	C	•	•	•	•	•	•	G	•	G
•	•	•	•	C	•	•	•	•	C	C	C	•	C	G	C	T	G	•	•		T	•	•	•	•	C	•	•	•	•	•	•	G	•	G
•	•	•	•	C	•	•	•	•	C	C	C	•	C	G	C	T	G	•	•		T	•	•	•	•	C	•	•	•	•	•	•	G	•	G
•	•	•	•	C	•	•	•	•	C	C	C	•	C	G	C	T	G	•	•		T	•	•	•	•	C	•	•	•	•	•	•	G	•	G
•	•	•	•	C	•	•	•	•	C	C	C	•	C	G	C	T	G	•	•		T	•	•	•	•	C	•	•	•	•	•	•	G	•	G
•	•	•	•	C	•	•	•	•	C	C	C	•	C	G	C	T	G	•	•		T	•	•	•	•	C	•	•	•	•	•	•	G	•	G
A	T	•	•	•	•	A	T	G	•	•	C	T	•	•	•	•	•	T	G		•	C	•	T	•	•	A	C	G	A	C	–	•	•	C
Relative position of nucleotide where nucleotide changes occur ^b^																																			
1112	1222		—1373/1374—			1383		1386	1410	1520		1521	1524		1526	1527	1562		1565	1704	1715		1720	1721	1723		1724	1725		1726	1730	1735		1747	1750
C	C		–	–	–	T		C	G	T		T	A		A	G	G		C	G	G		G	C	C		G	T		T	A	A		C	G
•	•		•	•	•	•		•	•	•		•	•		•	•	•		•	•	•		•	•	•		•	•		•	•	•		•	•
•	•		•	•	•	•		•	•	•		•	•		•	•	•		•	•	•		•	•	•		•	•		•	•	•		•	•
•	•		•	•	•	•		•	•	•		•	•		•	•	•		•	•	•		•	•	•		•	•		•	•	•		•	•
•	•		•	•	•		•	•	•	•		•	•		•	•	•		•	C	C		•	•	T		•	•		•	T	G		T	A
•	•		•	•	•	•		•	•	•		•	•		•	•	•		•	T	T		•	•	T		•	•		•	T	G		T	A
•	•		•	•	•	–		•	•	•		C	•		G	•	•		•	C	C		•	•	•		•	•		•	T	G		T	A
•	•		•	•	•	–		•	•	•		C	•		G	•	•		•	C	C		•	•	•		•	•		•	T	G		T	A
G	•		•	•	•	–		T	•	C		C	G		G	C	•		•	C	C		•	T	•		C	•		•	T	•		T	A
G	•		G	T	C	–		•	•	•		•	•		•	•	•		•	•	•		•	•	•		–	–		–	•	•		•	•
G	•		G	T	C	–		•	•	•		•	•		•	•	•		•	•	•		•	•	•		•	•		•	•	•		•	•
G	T		G	T	C	–		T	A	•		•	•		•	•	•		•	•	•		•	•	•		•	•		•	•	•		•	•
G	•		G	T	C	–		•	•	•		•	•		•	•	A		G	•	•		•	•	•		•	•		•	•	•		•	•
G	•		G	T	C	–		•	•	•		•	•		•	•	•		•	•	•		•	•	•		•	•		•	•	•		•	•
G	•		G	T	C	–		•	•	•		•	•		•	•	•		•	•	•		•	•	C		•	•		•	•	•		•	•
•	•		•	•	•	•		•	•	•		•	•		•	•	•		•	C	C		A	•	C		•	•		•	T	G		T	A

^a^ Lines of the same species are painted in a single background color: *P. neopolica* n. sp., light green; *P. plica*, light blue; *P. iharai* n. sp. pink; *P. toriii* n. sp., orange; *P. feliscati*, light orange; and *Liniscus himizu*, light gray. ^b^ Nucleotide position relative to the 5’-terminus of LC858133 (*P. neoplica* n. sp.). Dots indicate homologous nucleotides with the uppermost sequence (LC858133); dash indicates absence of nucleotide.

**Table 8:** Measurements of capillariid species from the urinary bladder of wild Carnivorra mammals in Japan (expressed in mm).

Species	*Pearsonema iharai* n. sp.	*Pearsonema toriii* n. sp.	*Pearsonema neoplica* n. sp.	*P. feliscati* (Bellingham, 1845)	*Pearsonema plica* (Rudolphi, 1819)	
Host	*Neogale vison*	*Meles anakuma*	*Nyctereutes procyonoides viverrinus*	*Procyon lotor*	*Meles meles*	*Vulpes* spp.
Locality	Japan (Fukushima)	Japan (Wakayama)	Japan (Wakayama)	Japan (Wakayama)	Europe	
Reference	Present study	Present study	Present study	Present study	Rukhlyadev (1948) in [3]	
**Male worms**	(*n* = 10)	(*n* = 4)	(*n* = 6)	(*n* = 4)	(*n* = ?)	(*n* = ?)
Worm length	16.19–26.19 (21.09)	14.48–18.82 (15.86)	19.51–21.45 (20.69)	20.54–24.23 (22.27)	21.06–32.94	27.00–37.80
Max. worm width	0.054–0.070 (0.065)	0.035–0.049 (0.043)	0.051–0.057 (0.053)	0.046–0.055 (0.050)	0.044–0.060	0.052–0.060
Esophagus length	4.90–6.49 (5.80)	3.85–5.46 (4.91)	5.78–7.19 (6.42)	4.06–7.17 (6.04)	6.66–7.92	9.00–10.26
P/A proportion ^a^	1.99–3.12 (2.62)	1.73–2.76 (2.27)	1.97–2.39 (2.23)	2.32–4.33 (2.85)	– ^b^	–
Spicule length	1.60–2.46 (2.18)	1.95–2.10 (2.03)	1.52–1.89 (1.67)	2.22–2.46 (2.33)	2.80–3.85	2.83–3.87
**Female worms**	(*n* = 11)	(*n* = 7)	(*n* = 10)	(*n* = 10)	(*n* = ?)	(*n* = ?)
Worm length	18.68–29.07 (23.85)	18.32–26.53 (23.50)	20.68–30.63 (23.56)	13.32–34.70 (22.91)	27.9–36.0	28.44–29.34
Max. worm width	0.104–0.148 (0.118)	0.080–0.120 (0.103)	0.070–0.91 (0.080)	0.047–0.108 (0.081)	0.081–0.085	0.088
Esophagus length	7.45–9.40 (8.24)	7.29–9.02 (8.08)	6.59–8.31 (7.77)	5.22–13.48 (9.22)	8.82–9.90	10.08–10.98
P/A proportion ^a^	1.41–2.24 (1.89)	1.50–2.27 (1.91)	1.48–2.97 (2.05)	1.21–2.08 (1.52)	–	–
Vulva from the esophageal end	0.071–0.148 (0.118)	0.214–0.667 (0.346)	0.052–0.235 (0.122)	0.073–0.525 (0.248)	–	–
Egg length	0.064–0.075 (0.069)	0.058–0.074 (0.067)	0.060–0.074 (0.067)	0.057–0.078 (0.063)	0.062–0.068	0.062–0.065
Egg width	0.026–0.034 (0.030)	0.027–0.031 (0.029)	0.025–0.031 (0.028)	0.022–0.031 (0.026)	0.029–0.031	0.026–0.028

^a^ P/A proportion: ratio of the posterior body to the anterior body. ^b^ No available data.

**Table 9:** Pairwise comparison of nucleotide variations in the SSU rDNA sequences of urinary bladder worms (*Pearsonema* spp. and *Liniscus himizu*) ^a^.

	Species	GenBank Accession No.	*P neoplica* n. sp.					*P. plica*	*P. iharai* n. sp.		*P. toriii* n.sp.	*Pearsonema feliscati*						*L. himizu*
			1	2	3	4	5	6	7	8	9	10	11	12	13	14	15	16
1	*P neoplica* n.sp.	LC858133 (1831 bp)		0 (0)	0 (0)	0 (0)	7 (0)	19 (0)	29 (8)	30 (14)	46 (7)	30 (25)	30 (25)	31 (21)	33 (21)	30 (25)	30 (21)	40 (7)
2	*P neoplica* n.sp.	LC858134 (1808 bp)	100		0 (0)	0 (0)	7 (0)	19 (0)	29 (8)	30 (14)	46 (7)	30 (25)	30 (25)	31 (21)	33 (21)	30 (25)	30 (21)	40 (7)
3	*P neoplica* n.sp.	LC858135 (1808 bp)	100	100		0 (0)	7 (0)	19 (0)	29 (8)	30 (14)	46 (7)	30 (25)	30 (25)	31 (21)	33 (21)	30 (25)	30 (21)	40 (7)
4	*P neoplica* n.sp.	LC858136 (1808 bp)	100	100	100		7 (0)	19 (0)	29 (8)	30 (14)	46 (7)	30 (25)	30 (25)	31 (21)	33 (21)	30 (25)	30 (21)	40 (7)
5	*P neoplica* n.sp.	LC052390 (1808 bp)	99.6	99.6	99.6	99.6		14 (0)	24 (8)	24 (14)	43 (7)	36 (24)	36 (24)	40 (21)	40 (21)	36 (24)	37 (21)	35 (7)
6	*P. plica*	MF621034 (1743 bp)	98.9	98.9	98.9	98.9	99.2		24 (8)	25 (14)	50 (7)	39 (24)	39 (24)	42 (21)	42 (21)	39 (24)	40 (21)	42 (7)
7	*P. iharai* n sp.	LC052386 (1810 bp)	98.4	98.4	98.4	98.4	98.7	98.7		3 (0)	25 (3)	31 (20)	31 (20)	33 (17)	33 (17)	31 (20)	31 (17)	35 (13)
8	*P. iharai* n.sp.	LC850896 (1816 bp)	98.3	98.3	98.3	98.3	98.7	98.6	99.8		31 (7)	39 (14)	39 (14)	41 (11)	40 (11)	39 (14)	39 (11)	40 (19)
9	*P. toriii* n.sp.	LC052387 (1813 bp)	97.5	97.5	97.5	97.5	97.6	97.1	98.6	98.3		47 (17)	47 (17)	48 (14)	52 (13)	47 (17)	49 (14)	51 (14)
10	*P. feliscati*	LC052388 (1824 bp)	98.3	98.3	98.3	98.3	98.0	97.8	98.3	97.8	97.4		0 (0)	3 (3)	2 (3)	0 (0)	0 (3)	50 (29)
11	*P. feliscati*	LC052389 (1827 bp)	98.3	98.3	98.3	98.3	98.0	97.8	98.3	97.8	97.4	100		3 (3)	2 (3)	0 (0)	0 (3)	50 (29)
12	*P. feliscati*	LC858137 (1850 bp)	98.3	98.3	98.3	98.3	97.8	97.6	98.2	97.7	97.4	99.8	99.8		5 (0)	4 (0)	3 (0)	53 (26)
13	*P. feliscati*	LC858138 (1850 bp)	98.2	98.2	98.2	98.2	97.8	97.6	98.2	97.8	97.1	99.9	99.9	99.7		0 (0)	0 (3)	53 (26)
14	*P. feliscati*	LC858139 (1850 bp)	98.3	98.3	98.3	98.3	98.0	97.8	98.3	97.8	97.4	100	100	99.8	100		1 (0)	50 (29)
15	*P. feliscati*	LC850894 (1827 bp)	98.4	98.4	98.4	98.4	98.5	97.7	98.3	97.9	97.3	100	100	99.8	100	99.9		51 (26)
16	*L. himizu*	LC850895 (1798 bp)	97.8	97.8	97.8	97.8	98.1	97.6	98.0	97.8	97.2	97.2	97.2	97.1	97.1	97.2	97.2	

^a^ Number of nucleotide substitutions (number of indel sites) in upper diagonal and nucleotide identity (%) in lower diagonal. Lines and columns of the same species are painted in a single background color: *P. neopolica* n. sp., light green; *P. plica*, light blue; *P. iharai* n. sp. pink; *P. toriii* n. sp., orange; *P. feliscati*, light orange; and *Liniscus himizu*, light gray.

## Data Availability

Parasite specimens collected in the present work were deposited in the National Museum of Nature and Science, Tokyo, Japan (NSMT-As4071–As4292, and AS4504-As4574). The nucleotide sequences obtained in the present study are available from the DDBJ/ EMBL/GenBank databases under the accession nos. LC052349–LC052390, LC425007, LC425008, LC850894–LC850897, and LC858133–LC858139.

## Supplementary Materials

The following supporting information can be downloaded at https://www.mdpi.com/article/10.3390/pathogens14050455/s1: Supplementary Table S1: Capillariid worm recovery from the gastrointestinal tract of wild mammals examined in the preset study; Supplementary Table S2: Measurements of male *Aonchotheca putorii* (types A and B) from different mammalian species in Japan; Supplementary Table S3: Measurements of *Aonchotheca* spp. from the stomach of wild boars in Japan (expressed in mm); Supplementary Table S4: Measurements of *Aonchotheca* spp. from the abomasum/small intestine of ruminants (expressed in mm); Supplementary Table S5: Measurements of *Pearsonema* spp. from the urinary bladder of Carnivora mammals (expressed in mm); Supplementary Table S6: Measurements of *Echinocoleus* spp. from the alimentary tracts of mammals (expressed in mm); Supplementary Table S7: Measurements of *Eucoleus* spp. from mammals (expressed in mm); Supplementary Table S8: Comparison of nucleotide variations in the SSU rDNA sequences of *Eucoleus* spp. from different mammalian species in Japan; Supplementary Figure S1: Location of intraspecific nucleotide changes in *Aonchotheca putorii* (genotypes Ia, Ib, II, III, and IV; see Table 1) in a putative secondary structure of the SSU rDNA (DDBJ/EMBL/GenBank accession no. LC052349); Supplementary Figure S2: Morphology of *Aonchotheca bilobate*; Supplementary Figure S3: Morphology of *Liniscus himizu* male worm; Supplementary Figure S4: *Calodium hepaticum* infection in brown rat livers; Supplementary Figure S5: Morphology of *Trichosomoides crassicauda* female worm collected from the urinary bladder of a brown rat; and Supplementary Figure S6: Frequency of nucleotide changes (substitutions and indels) in capillariid SSU rDNA sequences.
