# Supplementary material for: Integrated Taxonomic Approaches to Gastrointestinal and Urinary Capillariid Nematodes from Wild and Domestic Mammals
Source: Pathogens. 2025 May 6;14(5):455. doi: 10.3390/pathogens14050455 (PMC12114645; doi:10.3390/pathogens14050455)
Supplement: Supplementary file 1 [file pathogens-14-00455-s001.zip › pathogens-3558869-supplementary.pdf]

Suppl. Table S1. Worm recovery of capillariid worms from the gastrointestinal tract of wild mammals examined in the preset study

Host	Locality	Date of animal collection	Number of animals examined	Number of positive animals (%)	Parasite species
Canidae					
Raccoon dog	Aomori and Akita Prefs	Sep. 1997–May 2000	34	2 (5.9 %)	
	Gunma Pref.	Apr. 2009	1	1 (100 %)	*A. putorii*
	Wakayama Pref.	May 2003–Feb. 2008	96	4 (4.2 %)	*A. suzukii* n. sp.
	Shikoku Is.	2004–2009	18	2 (11.1 %)	*P. neoplica* n. sp.
	Saga Pref.	Oct.–Nov. 2009	4	2 (50.0 %)	
				2 (50.0 %)	*Ec. yokoyamae* n. sp.
Raccoon	Wakayama Pref.	May 2003–Nov. 2006	919	1 (0.1 %)	
	Hyogo Pref.	Jan. 2004–Nov. 2006	262	8 (3.1 %)	
		Nov. 2008–Dec. 2010	305	8 (2.6 %)	*A. putorii*
	Osaka Pref.	Jan. 2005–Oct. 2006	121	10 (8.3 %)	*A. suzukii* n. sp.
	Shimane Pref.	Jul. 2009–Sep. 2012	155	12 (7.7 %)	
	Nagasaki Pref.	Feb. 2009–Nov. 2011	83	13 (15.7 %)	
	Saga Pref.	Aug. 2010–Nov. 2011	166	53 (31.9 %)	
Felidae					
Domestic cat	Wakayama Pref.	Feb. 2009	1	1 (100 %)	*A. putorii*<br>*A. suzukii* n. sp.
				1 (100 %)	*Ec. yokoyamae* n. sp.
Mustelidae					
Japanese badger	Shiga Pref.	Nov. 2009	1	1 (100 %)	
	Wakayama Pref.	Jul. 2003–Dec. 2009	21	5 (23.8 %)	*A. putorii*
	Kochi Pref.	Apr. 2004–Mar. 2008	12	1 (8.3 %)	*A. suzukii* n. sp.
	Saga Pref.	Oct. 2009	3	3 (100 %)	
	Wakayama Pref.	Jul. 2003–Dec. 2009	(21)	1 (4.8 %)	*Ec. yokoyamae* n. sp.
			(21)	4 (19.0 %)	*Eu. kaneshiroi* n. sp.
	Saga Pref.	Oct. 2009	(3)	3 (100 %)	*Eu. kaneshiroi* n. sp.
Siberian weasel	Wakayama Pref.	Jul. 2000–Jan. 2008	14	1 (7.1 %)	*A. putorii*
Japanese weasel	Wakayama Pref.	Jan. 2005–Dec. 2007	8	0	—
Japanese marten	Aomori and Akita Prefs	Sep. 1997–May 2000	67	48 (71.6 %)	*A. putorii*
	Wakayama Pref.	Mar.2003–Aug. 2008	34	6 (17.6 %)	
American mink	Fukushima Pref.	Aug.–Oct. 2010	74	1 (1.3 %)	*A. putorii*
				29 (39.2 %)	*P. iharai* n. sp.
Suidae (Cetartiodactyla)					
Japanese wild boar	Hyogo Pref.	Aug.2007–Dec. 2010	132	83 (62.9 %)	Capillariid worms
			14	9 (64.3 %)	*A. suis*
				11 (78.6 %)	*A. riukiuensis*
				2 (14.3 %)	*A. suzukii* n. sp.
	Wakayama Pref.	Dec. 2003-May 2005	25	17 (68.0 %)	*A. suis*
				20 (80.0 %)	*A. riukiuensis*
				4 (16.0 %)	*A. suzukii* n. sp.
				1 (4.0 %)	*Ec. yokoyamae* n. sp.
				1 (4.0 %)	*Eu. kaneshiroi* n. sp.
Erinaceidae (Insectivora)					
Manchurian hedgehog	Shizuoka Pref.	Sep.–Nov. 2007	31	18 (58.1 %)	*A. putorii*

Suppl. Table S2a. Measurements of male *Aonchotheca putorii* (types A and B) from different mammalian species in Japan

Type of worms	Type A	Type A	Type A	Type A	Type B	Type B	Type B
Host species	*Martes melampus*	*Felis silvastris catus*	*Erinaceus amurensis*	*Mustela sibirica*	*Procyon lotor*	*Martes melampus*	*Mustela sibirica*
Location	Stomach, S.I.	Stomach, S.I.	Stomach, S.I.	Stomach, S.I.	Stomach, S.I.	Stomach, S.I.	Stomach, S.I.
Locality	Aomori, Akita	Wakayama	Shizuoka	Kochi	Saga, Nagasaki	Aomori, Akita	Kochi
**Male worms**	(n = 14)	(n = 10)	(n = 10)	(n = 6)	(n = 3)	(n = 4)	(n = 3)
Worm length	6.00–7.56 (6.88)	4.47–6.60 (5.45)	5.73–7.26 (6.56)	4.58–5.32 (5.01)	5.48-6.05 (5.84)	6.00-6.74 (6.22)	4.47-4.82 (4.67)
Max. worm width	0.039–0.056 (0.046)	0.039–0.046 (0.042)	0.046–0.070 (0.061)	0.037–0.048 (0.041)	0.045-0.050 (0.047)	0.042-0.055 (0.047)	0.044-0.046 (0.045)
Esophagus length	2.82–3.78 (3.33)	2.25–3.56 (2.79)	2.60–3.40 (3.06)	2.44–2.71 (2.58)	2.60-3.01 (2.85)	2.74-2.82 (2.78)	2.16-2.47 (2.33)
P/A proportion	0.968–1.231 (1.070)	0.835–1.084 (0.955)	1.018–1.294 (1.146)	0.796–1.090 (0.943)	1.009-1.105 (1.051)	1.146-1.412 (1.236)	0.911-1.063 (1.007)
Spicule length	0.375–0.482 (0.425)	0.323–0.395 (0.365)	0.301–0.345 (0.321)	0.312–0.400 (0.339)	0.205-0.225 (0.215)	0.208-0.247 (0.227)	0.203-0.208 (0.205)

Suppl. Table S2b. Measurements of female *Aonchotheca putorii* from different mammalian species in Japan

Type of worms	Types A & B	Type A	Type A	Types A & B	Types A & B	Type B
Host species	*Martes melampus*	*Felis silvastris catus*	*Erinaceus amurensis*	*Mustela sibirica*	*Meles anakuma*	*Nyctereutes v. viverrinus*
Location	Stomach, S.I.	Stomach, S.I.	Stomach, S.I.	Stomach, S.I.	Stomach, S.I.	Stomach, S.I.
Locality	Aomori, Akita	Wakayama	Shizuoka	Kochi	Kyoto, Saga	Wakayama
**Female worms**[a]	(n = 19)	(n = 18)	(n = 9)	(n = 9)	(n = 7)	(n = 8)
Worm length	5.89–10.14 (9.06)	6.60–8.63 (7.83)	9.07–12.16 (11.00)	5.32–7.32 (6.42)	5.70–10.93 (9.34)	7.15–8.44 (7.84)
Max. worm width	0.058–0.069 (0.064)	0.057–0.070 (0.063)	0.079–0.112 (0.093)	0.051–0.066 (0.056)	0.043–0.077 (0.058)	0.054–0.058 (0.056)
Esophagus length	3.15–3.70 (3.42)	2.41–3.40 (3.08)	3.64–4.33 (3.97)	2.19–3.04 (2.72)	2.63–4.03 (3.58)	2.66–3.23 (2.90)
P/A proportion	1.44–1.93 (1.65)	1.38–1.743 (1.55)	1.47–1.95 (1.77)	1.21–1.56 (1.37)	1.17–1.76 (1.61)	154–1.94 (1.70)
Vulva from the esophageal end	0.049–0.126 (0082)	0.047–0.110 (0.081)	0.071–0.129 (0.101)	0.041–0.088 (0.061)	0.044–0.112 (0.068)	0.066–0.142 (0.094)
Egg length	0.058–0.069 (0.064)	0.053–0.064 (0.059)	0.059–0.070 (0.064)	0.059–0.070 (0.063)	0.056–0.068 (0.062)	0.060–0.068 (0.064)
Egg width	0.024–0.034 (0.028)	0.024–0.030 (0.026)	0.030–0.036 (0.026)	0.022–0.028 (0.026)	0.023–0.030 (0.026)	0.025–0.029 (0.027)

[a] Type of female worms were presumed based on the type of male worms isolated from the same animals.

Suppl. Table S3. Measurements of *Aonchotheca* spp. from the stomach of wild boars in Japan (expressed in mm)

Species	*Aonchotheca suis*			*Aonchotheca riukiuensis*		*Aonchotheca suzukii* n.sp.
Locality	Japan (Wakayama, Hyogo)	Japan (Wakayama)	Japan (Wakayama, Yamaguchi)	Japan (Wakayama, Hyogo)	Japan (Okinawa)	Japan (Wakayama, Hyogo)
Reference	Present study		[83]	Present study	[84]	
**Male worms**	(n = 8)	(n = 2)[a]	(n = ?)	(n = 9)	(n = 4)	(n = 2)
Worm length	11.89–13.53 (13.02)	9.56–9.84	13.0–14.5	7.29–13.75 (11.47)	10.3–12.9	6.90–7.59
Max. worm width	0.054–0.074 (0.066)	0.052–0.053	0.060–0.075	[+H7]	0.028–0.041	0.050–0.061
Esophagus length	4.60–5.89 (5.16)	4.08–4.41	5.4–5.7	3.59–5.18 (4.59)	4.0–5.8	3.21–3.70
P/A proportion	1.30–1.69 (1.53)	1.23–1.34	1.3–1.6	1.03–1.82 (1.49)	1.2–1.6	1.05–1.15
Spicule length	0.75–0.82 (0.79)	0.73–0.81	0.78–0.85	0.71–1.01 (0.86)	0.81–0.98	0.390–0.460
**Female worms**	(n = 23)		(n = ?)	(n = 34)	(n = 7)	(n = 6)
Worm length	13.32–22.77 (18.65)		19–22	13.92–36.11 (27.83)	18.8–24.3	8.96–9.73 (9.22)
Max. worm width	0.063–0.100 (0.083)		0.08–0.09	0.044–0.085 (0.069)	0.048–0.054	0.053–0.061 (0.056)
Esophagus length	4.88–6.44 (5.87)		5.8–6.5	4.38–6.27 (5.24)	4.3–4.9	2.66–3.12 (2.90)
P/A proportion	1.64–2.93 (2.18)		2.2–2.5	2.18–5.14 (4.29)	3.3–4.4	1.92–2.39 (2.18)
Vulva from the esophageal end	0.044–0.203 (0.139)		0.12–0.15	0–0.236 (0.104)	0.043–0.112	0.082–0.132 (0.110)
Egg length	0.050–0.070 (0.063)		0.057–0.072	0.049–0.064 (0.059)	0.050–0.056	0.054–0.066 (0.059)
Egg width	0.024–0.033 (0.028)		0.024–0.030	0.020–0.033 (0.027)	0.024–0.028	0.024–0.027 (0.025)

Suppl. Table S4. Measurements of *Aonchotheca* spp. from the abomasum / small intestine of ruminants (expressed in mm)

Species	*Aonchotheca bilobata* (Bhalerao, 1933) Moravec, 1982					*Aonchotheca musimon* Pisanu & Bain, 1999	*Aonchotheca bovis* (Schnyder, 1906) Moravec, 1982			
Original species name when reported	—		*Capillaria bilobata* Bhalerao, 1933	*Capillaria megrelica* Rodonaja, 1947	*Aonchotheca bilobata* (Bhalerao, 1933) Moravec, 1982	*Aonchotheca musimon* Pisanu & Bain, 1999	*Capillaria bovis* (Schnyder, 1906) Ransom, 1911	*Aonchotheca bovis* (Schnyder, 1906) Moravec, 1982	*Capillaria brevipes* Ransom, 1911	*Capillaria longipes* Ransom, 1911
Host species	*Capra hircus*		*Bos taurus*	*Capra hircus*	*Bison bonasus*	*Ovis musimon*	*Bos taurus, Ovis aries, Antilocapra americana*	*Cervus elaphus*	*Ovis aries*	*Ovis aries, Antilocapre americana*
Site of infection	Abomasum	Upper small intestine	Abomasum	Abomasum	Abomasum	Pyloric junction of the duodenum	Small intestine	Small intestine	Small intestine	Small intestine
Locality	Japan (Yamaguchi)		India	Georgia (Europe)	Poland (Natural Park, Bialowieza)	Kerguelen Archipelago, District of the French Southern and Antarctic Lands	Europe, U.S.A.	France (Ardennes)	U.S.A.	U.S.A.
Reference	Present study		[1]	[3]	[90]	[90]	[2]	[88]	[86]	[86]
**Male worms**	(n = 4)	(n = 10)	(n = ?)	(n = ?)	(n = 5)	(n = 5)	(n = ?)	(n = 4)	(n = ?)	(n = ?)
Worm length	12.03–12.71 (12.32)	9.53–13.64 (12.33)	10.1–16.5	11.8	12.5–14.5	12.1–14.9 [13.9]	11.9	14.00–16.60	8–9	11–13
Max. worm width	0.050–0.060 (0.053)	0.054–0.068 (0.062)	0.050–0.083	0.058	0.053–0.067	0.044–0.052 [0.047]	0.062	0.065	0.050	0.050–0.060
Esophagus length	4.49–5.34 (4.87)	4.03–5.34 (4.72)	5.2–8.0	5	6.20–7.20	4.64–5.00 [4.59]	–	5.90–6.80	4	4–5
P/A proportion	1.32–1.70 (1.54)	1.37–1.94 (1.62)	1.00	–	–	–	–	–	1.00–1.25	–
Spicule length	0.22–0.23 (0.22)	0.21–0.23 (0.22)	0.19–0.24	0.2	0.189–0.213	0.208–0.230 [0.220]	1.09	1.10–1.20	0.9	1.2
**Female worms**	(n = 4)	(n = 8)	(n = ?)	(n = ?)	(n = 5)	(n = 5)	(n = ?)	(n = 3)	(n = ?)	(n = ?)
Worm length	19.37–21.95 (20.68)	19.75–21.84 (20.88)	14.0–21.3	18–20	17.8–19.7	17.5–21.4 [20.2]	18.72–21.83	24.70–28.90	12	20
Max. worm width	0.066–0.080 (0.071)	0.064–0.086 (0.074)	0.072–0.085	0.090	0.036–0.046	0.056–0.058 [0.055]	0.078-0.100	0.070–0.080	0.060	0.08
Esophagus length	4.96–5.21 (5.05)	4.66–5.53 (5.02)	6.5–9.4	(7.5–8.0)	7.25–8.28	4.92–5.28 [4.84]	6.68–8.12	7.20–8.30	5	6–7
P/A proportion	2.80–3.35 (3.09)	2.69–3.48 (3.17)	1.02	–	–	–	–	–	1.4	1.86–2.33
Vulva from the esophageal end	0.055–0.068 (0.063)	0.060–0.099 (0.076)	0.055–0.075	–	–	– [0.075]	0.179–0.232	0.150–0.230	–	–
Egg length	0.054–0.061 (0.057)	0.054–0.063 (0.058)	0.033–0.053	0.053–0.055	0.052–0.056	0.059–0.060 [0.060]	0.045–0.052	0.050–0.054	0.050	0.045–0.050
Egg width	0.024–0.030 (0.026)	0.023–0.032 (0.027)	0.014–0.021	0.018–0.020	0.022–0.024	0.026–0.028 [0.028]	0.022–0.030	0.024–0.027	0.025	0.022–0.025

Suppl. Table S5. Measurements of *Pearsonema* spp. from the urinary bladder of Carnivora mammals (expressed in mm)

Species	*P. neoplica* n. sp.	*P. plica* (Rudolphi, 1819)		*P. feliscati* (Bellingham, 1845)		*P. linsi* (Freitas et Lent, 1935)	*P. mucronata* (Molin, 1858) Moravec, 1982	*P. pearsoni* Freitas et Mendonca, 1960
Host	*Nyctereutes procyonoides viverrinus*	*Vulpes vulpes*	*Procyon lotor*	*Procyon lotor*	*Felis* spp.	*Grison vittata* (Mustelidae)	Stone marten, ermine, sable, polecat, mink	*Procyon cancrivorous*
Locality	Japan (Wakayama)	Canada (Ontario)	Canada (Ontario)	Japan (Wakayama)				
Reference	Present study	[4]		Present study	[1]	[3]	{3]	[93]
**Male worms**	(n = 6)	(n = 15)	(n = 15)	(n = 4)	(n = ?)	(n = ?)	(n = ?)	(n = ?)
Worm length	19.51–21.45 (20.69)	28.6–53.3 (39.9)	16.7–31.2 (22.6)	20.54–24.23 (22.27)	25.5	15.6-18.0	28.70-34.82	9.61
Max. worm width	0.051–0.057 (0.053)	0.055–0.070 (0.060)	0.031–0.048 (0.041)	0.0467–0.055 (0.050)	0.032–0.064	0.024-0.048	0.065-0.074	0.043
Esophagus length	5.78–7.19 (6.42)	7.7–11.7 (9.3)	3.5–6.8 (5.4)	4.06–7.17 (6.04)	6.7	4.9-5.9	6.47-6.83	4.59
P/A proportion	1.97–2.39 (2.23)	– (3.29)	– (3.19)	2.32–4.33 (2.85)	3	2.1		1.09
Spicule length	1.52–1.89 (1.67)	3.4–5.2 (4.5)	2.3–3.5 (2.9)	2.22–2.46 (2.33)	2.5	1.64-1.74	3.062-6.178	1.00-1.07
**Female worms**	(n = 10)	(n = 15)	(n = 15)	(n = 10)	(n = ?)	(n = ?)	(n = ?)	(n = ?)
Worm length	20.68–30.63 (23.56)	29.4–52.2 (42.7)	17.6–44.9 (25.9)	13.32–34.70 (22.91)	28.6–31.9	18-21.5	35.17-38.24	9.58
Max. worm width	0.070–0.91 (0.080)	0.089–0.114 (0.100)	0.060–0.109 (0.070)	0.047–0.108 (0.081)	0.032–0.144	0.057-0.115	0.13	0.087
Esophagus length	6.59–8.31 (7.77)	6.9–11.9 (9.9)	4.4–8.4 (6.6)	5.22–13.48 (9.22)	10.2–10.8	7.7-8	7.97	5.66
P/A proportion	1.48–2.97 (2.05)	– (3.31)	– (2.92)	1.21–2.08 (1.52)	2	1.6		0.69
Vulva from the esophageal end	0.052–0.235 (0.122)	–	–	0.073–0.525 (0.248)	0.034–0.544	0.112-0.256		
Egg length	0.060–0.074 (0.067)	0.058–0.071 (0.065)	0.059–0.074 (0.064)	0.057–0.078 (0.063)	0.051–0.062	0.057-0.068	0.065-0.068	0.063-0.067
Egg width	0.025–0.031 (0.028)	0.025–0.031 (0.028)	0.023–0.028 (0.026)	0.022–0.031 (0.026)	0.024–0.032	0.024-0.040	0.028-0.031	0.027-0.034

Suppl. Table S6. Measurements of *Echinocoleus* spp. from the alimentary ducts of mammals (expressed in mm)

Species	*Echinocoleus yokoyamae* n. sp. Badger (stomach)	*Echinocoleus yokoyamae* n. sp. Wild boar (stomach)	*Echinocoleus hydrochoerid* (Travassos, 1916) Moravec, 1982	*Echinocoleus auritae* (Travassos, 1914) López-Neyra, 1947
Host	*Meles anakuma, Nyctereutes procyonoides viverrinus,* cat	*Sus scrofa leucomystax*	*Hydrochoerus hydrochaeris*	*Didelphis aurita*
Location	Stomach	Stomach	Stomach and small intestine	Small intestine
Locality	Japan (Wakayama, Saga)	Japan (Wakayama)	Argentina	Brazil
Reference	Present study	Present study	[107]	[2, 107]
**Male worms**	(n = 9)	(n = 4)	(n = 6)	(n = ?)
Worm length	5.75–8.44 (7.21)	9.92–10.66 (10.29)	16.67–20.35 (18.65)	10.2–11.5
Max. worm width	0.035–0.050 (0.041)	0.042–0.054 (0.047)	0.050–0.060 (0.055)	0.021–0.078
Esophagus length	3.07–3.97 (3.49)	3.62–4.88 (4.06)	6.85–8.42 (7.58)	5.0–5.5
P/A proportion	0.88–1.20 (1.07)	1.19– 1.78 (1.56)	– (1.46)	1.04 – 1.09 (1.07)
Spicule length	weakly sclerotized, unclear	weakly sclerotized, unclear	1.33–2.00 (1.55)	– (1.1)
**Female worms**	(n = 9)	(n = 7)	(n = 6)	(n = ?)
Worm length	7.32–9.26 (8.61)	10.47–11.53 (10.95)	32.28–41.93 (37.58)	12.9–16.8
Max. worm width	0.046–0.060 (0.052)	0.052–0.068 (0.059)	0.070–0.100 (0.085)	0.042–0.114
Esophagus length	3.32–4.11 (3.90)	3.89–4.47 (4.17)	7.61–8.72 (8.20)	4.9–7.0
P/A proportion	1.11–1.30 (1.21)	1.47 – 1.75 (1.63)	– (3.58)	1.40 – 1.63 (1.50)
Vulva from the esophageal end	0.027–0.079 (0.057)	0.030 – 0.148 (0.099)	0.040–0.100 (0.069)	0.071–0.157
Egg length	0.053–0.063 (0.058)	0.050–0.058 (0.053)	0.046–0.050	0.052–0.062
Egg width	0.024–0.032 (0.027)	0.025–0.028 (0.026)	0.023–0.030	0.020–0.024

a

Suppl. Table S7. Measurements of *Eucoleus* spp. from mammals (expressed in mm)

Species	*Eucoleus kaneshiroi* n. sp. Toda-2010b_Badger (small intestine)	*Eucoleus auritae* (Travassos, 1914) comb. n.	*Eucoleus procyonis* (Pence, 1975) Moravec, 1982	*Eucoleus procyonis* (Pence, 1975) Moravec, 1982
Host	*Meles anakuma*	*Didelphis aurita, Metachirops opossum*	*Procyon lotor, Mephitis mephitis*	*Procyon lotor*
Location	Small intestine	Small intestine	Esophageal epithelium	Esophagus
Locality	Japan (Wakayama, Saga)	Brazil	USA (Louisiana)	Canada (Ontario)
Reference	Present study	[3]	[115]	[4]
**Male worms**	(n = 17)	(n = ?)	(n = 5)	(n ≥ 5)
Worm length	9.12–12.19 (10.42)	11.2–11.5	4.90–7.56 (6.28)	7.7–10.5 (9.2)
Max. worm width	0.044–0.059 (0.052)	0.021–0.078	0.047–0.050 (0.049)	0.054–0.061 (0.057)
Esophagus length	4.41–5.86 (5.31)	5.0–5.5	1.89–2.03 (1.88)	2.4–3.1 (2.8)
P/A proportion	0.78 – 1.17 (0.97)	– (1.07)	2.5–3.4 (2.8)	– (2.29)
Spicule length	weakly sclerotized, unclear	1.104 (weakly sclerotized, unclear)	0.800–0.850	0.732–1.551 (0.954)
**Female worms**	(n = 8)	(n = ?)	(n = 10)	(n ≥ 5)
Worm length	14.19–18.33 (16.05)	12.9–16.8	7.25–11.78 (9.05)	11.4–17.9 (14.1)
Max. worm width	0.070–0.091 (0.077)	0.042–0.144	0.048–0.075 (0.062)	0.069–0.100 (0.084)
Esophagus length	4.49–6.41 (5.50)	4.9–7.0	2.00–2.85 (2.53)	3.2–4.4 (3.7)
P/A proportion	1.49–2.19 (1.94)	–	3.3–4.3 (3.6)	– (2.81)
Vulva from the esophageal end	0.033–0.164 (0.082)	0.00.071–0.157	–	–
Egg length	0.062–0.079 (0.069)	0.052–0.062	0.053–0.060 (0.056)	0.054–0.068 (0.061)
Egg width	0.028–0.037 (0.033)	0.020–0.024	0.021–0.035 (0.026)	0.023–0.029 (0.025)

(Continued)

Species	*Eucoleus schvalovoj* Kontrimavichus, 1963	*Eucoleus aerophilus* (Creplin, 1839) Dujardin, 1845	*Eucoleus didelphis* (Butterworth et Beverley-Burton, 1977) Moravec, 1982	*Eucoleus boehmi* (Supperer, 1953) Moravec, 1982
Host	*Lutra lutra*	*Vulpes vulpes*	*Didelphis virginiana*	*Vulpes vulpes, Vulves argentina*
Location	Esophageal epithelium	Trachea, bronchus	Bronchioles, Pulmonary alveolus	Nasal sinuses
Locality	Russian Far East (Khabarovsk), Spain (various provinces)	Canada (Ontario)	Canada (Ontario)	Europe (Austria)
Reference	[116]	[4]	[4]	[111]
**Male worms**	(n = 9)	(n ≥ 5)	(n = ?)	(n = 3?)
Worm length	7.2–8.3 (7.7)	17.7–25.1 (21.6)	8.8–9.7	15–25
Max. worm width	0.057–0.067 (0.061)	0.076–0.098 (0.087)	0.058–0.068 (0.063)	0.075–0.113
Esophagus length	2.2–3.1 (2.7)	5.4–7.6 (6.8)	3.9–4.5	–
P/A proportion	1.6–2.3 (1.8)	– (2.18)	–	1.5
Spicule length	0.660–0.845 (0.724)	0.605–1.410 (1.049)	0.106–0.189 (0.143)	1.175
**Female worms**	(n = 10)	(n ≥ 5)	(n = ?)	(n = 5?)
Worm length	9.1–11.3 (9.8)	21.0–47.6 (37.1)	21.9–34.6	30–41.3
Max. worm width	0.082–0.103 (0.091)	0.104–0.186 (0.140)	0.104–0.125	0.120–0.210
Esophagus length	3.0–3.6 (3.3)	4.5–9.2 (7.1)	4.6–5.6	6–7
P/A proportion	1.7–2.3 (2.0)	– (4.23)	–	5–5.9
Vulva from the esophageal end	0.035–0.0725 (0.053)	–	–	–
Egg length	0.056–0.064 (0.060)	0.064–0.083 (0.073)	0.054–0.069	0.063–0.065
Egg width	0.026–0.031 (0.0286)	0.026–0.038 (0.034)	0.025–0.029	0.030–0.034

(Continued)

Species	*Eucoleus eberthi* (Freitas et Lent, 1935) López-Neyra, 1947	*Eucoleus fluminensis* (Freitas, 1946) López-Neyra, 1947	*Eucoleus garfiai* (Gallego et Mas-Coma, 1975) Moravec, 1982	*Eucoleus tenuis* Dujardin, 1845
Host	*Metachirops opossum*	*Didelphis marsupialis*	*Sus scrofa*	*Erinaceus europaeus*
Location	Esophagus	Frondal sinuses	Tongue	Bronchus
Locality	Brazil	Brazil	Europe (Spain, Austria), Japan	Europe
Reference	[3]	[3]	[19, 114]	[3]
**Male worms**	(n = 1)	(n = 1)	(n = 15)	(n = ?)
Worm length	15.86	38.86	6.84–10.73 (9.50)	8.5–15.5
Max. worm width	0.016–0.110	0.1	0.062–0.087 (0.070)	0.068–0.096
Esophagus length	4.24	8.21	2.79–3.60 (3.19)	2.5
P/A proportion	1.27	3.7	1.31–2.35 (1.98)	5
Spicule length	0.328	weakly sclerotized, unclear	weakly sclerotized, unclear	weakly sclerotized, unclear
**Female worms**	(n = 1)	(n = ?)	(n = 15)	(n = ?)
Worm length	21.45	69.85–71.56	11.02–14.80 (12.76)	12.9
Max. worm width	0.049–0.139	0.133–0.149	0.074–0.107 (0.093)	0.108–0.112
Esophagus length	4.84	9.04–9.11	2.94–3.42 (3.16)	–
P/A proportion	3.4	6.1–6.7	2.63–3.55 (3.03)	3
Vulva from the esophageal end	–	0.19–0.29	0.034–0.091 (0.064)	–
Egg length	0.059–0.062	0.055–0.059	0.047–0.056 (0.052)	0.056–0.072
Egg width	0.024–0.029	0.023–0.027	0.021–0.029 (0.025)	0.029–0.033

Suppl. Table 8. Comparison of nucleotide variations in the SSU rDNA sequences of *Eucoleus* spp. from different mammalian species in Japan

Parasite	Host	GenBank accession no.	Sequence length (bp)	Relative position of nucleotide where nucleotide changes occur[a]																								
				51	52	53	54	55	92	117	210	214	215	219	220	221	222	223	224	226	227	228	230	236	237	238	239	240
Eucoleus sp. Toda-2010d	*Procyon lotor* (Japan)	LC052384	1,819	G	G	C	A	A	T	G	T	C	G	G	C	G	C	G	C	C	T	G	C	T	C	G	T	C
Eucoleus aerophilus	*Mustela sibirica* (Japan)	LC052385	1,814	•	–	T	G	T	•	A	C	•	T	–	–	–	–	–	–	G	A	A	A	G	–	–	–	–
Eucoleus aerophilus	*Vulpes vulpes* (Australia)	MW709573	1,814	•	–	T	•	T	•	A	C	•	T	–	–	–	–	–	–	G	A	A	A	A	–	–	–	–
Eucoleus aerophilus	*Vulpes vulpes* (Germany)	MF599385	1,749	•	–	T	•	T	C	A	C	•	T	–	–	–	–	–	–	G	A	A	A	A	–	–	–	–
Capillariid sp. cat-2018	*Felis catus* (Australia)	MW709574	1,820	A	–	T	G	T	•	A	C	T	T	–	–	–	–	–	–	G	A	A	G	A	–	–	–	–

(Suppl. Table S8—*continued*)

Parasite	GenBank accession no.	Sequence length (bp)	Relative position of nucleotide where nucleotide changes occur[a]																							
			247	251/ 252	251/ 252	252	253	270	271	272	547	548	749	754	836	869/ 870	869/ 870	930	1,383	1,389	1,399	1,736	1,739	1,739/ 1,740	1,739/ 1,740	1,739/ 1,740	1,739/ 1,740
Eucoleus sp. Toda-2010d	LC052384	1,819	T	–	–	A	A	G	A	A	C	G	T	A	C	–	–	G	A	G	T	T	T	–	–	–	–
Eucoleus aerophilus	LC052385	1,814	•	G	G	•	•	A	G	•	T	A	C	T	•	A	A	•	G	C	C	•	-	•	T	T	G
Eucoleus aerophilus	MW709573	1,814	•	•	•	•	•	A	G	•	T	A	C	T	•	A	A	•	G	•	C	C	C	T	T	T	G
Eucoleus aerophilus	MF599385	1,749	•	•	•	•	•	A	G	•	T	A	C	T	T	A	A	A	G	•	C	C	C	T	T	T	G
Capillariid sp. cat-2018	MW709574	1,820	C	•	•	G	G	A	G	G	•	•	C	•	•	A	A	•	G	•	C	C	•	•	•	•	•

[a] Nucleotide position relative to the 5′-terminus of LC052384 (*Eucoleus* sp. Toda-2010d). Dots indicate homologous nucleotides with the uppermost sequence (LC052384); dash indicates absence of nucleotide.

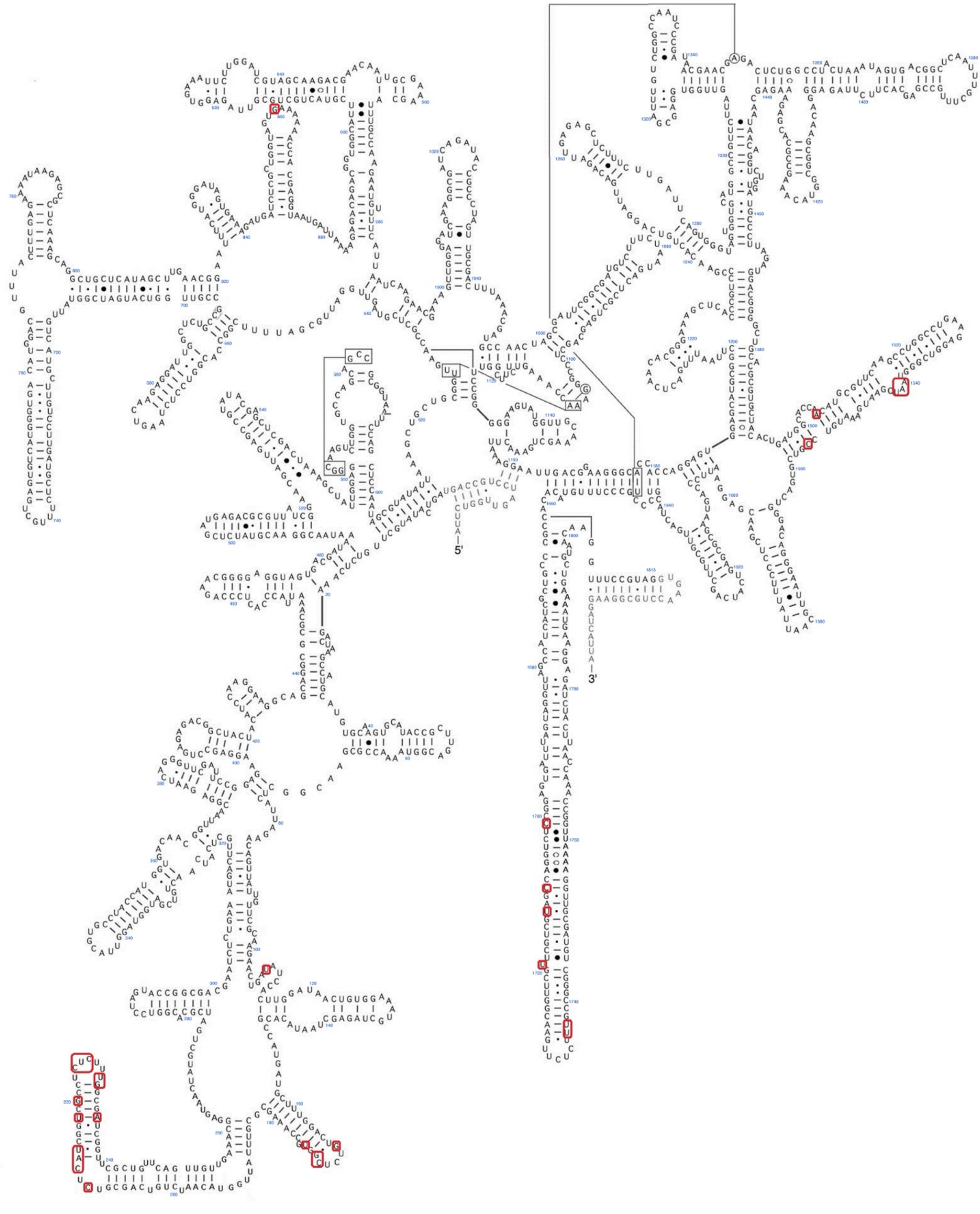

Suppl. Fig. S1. Location of intraspecific nucleotide changes of *Aonchotheca putorii* (genotypes Ia, Ib, II, III, and IV; see Table 1) on a putative secondary structure of the SSU rDNA (DDBJ/EMBL/ GenBank accession no. LC052349). Sites of nucleotide substitutions and indels by different genotypes, relative to the genotype Ia (LC052349), are indicated by red circles.

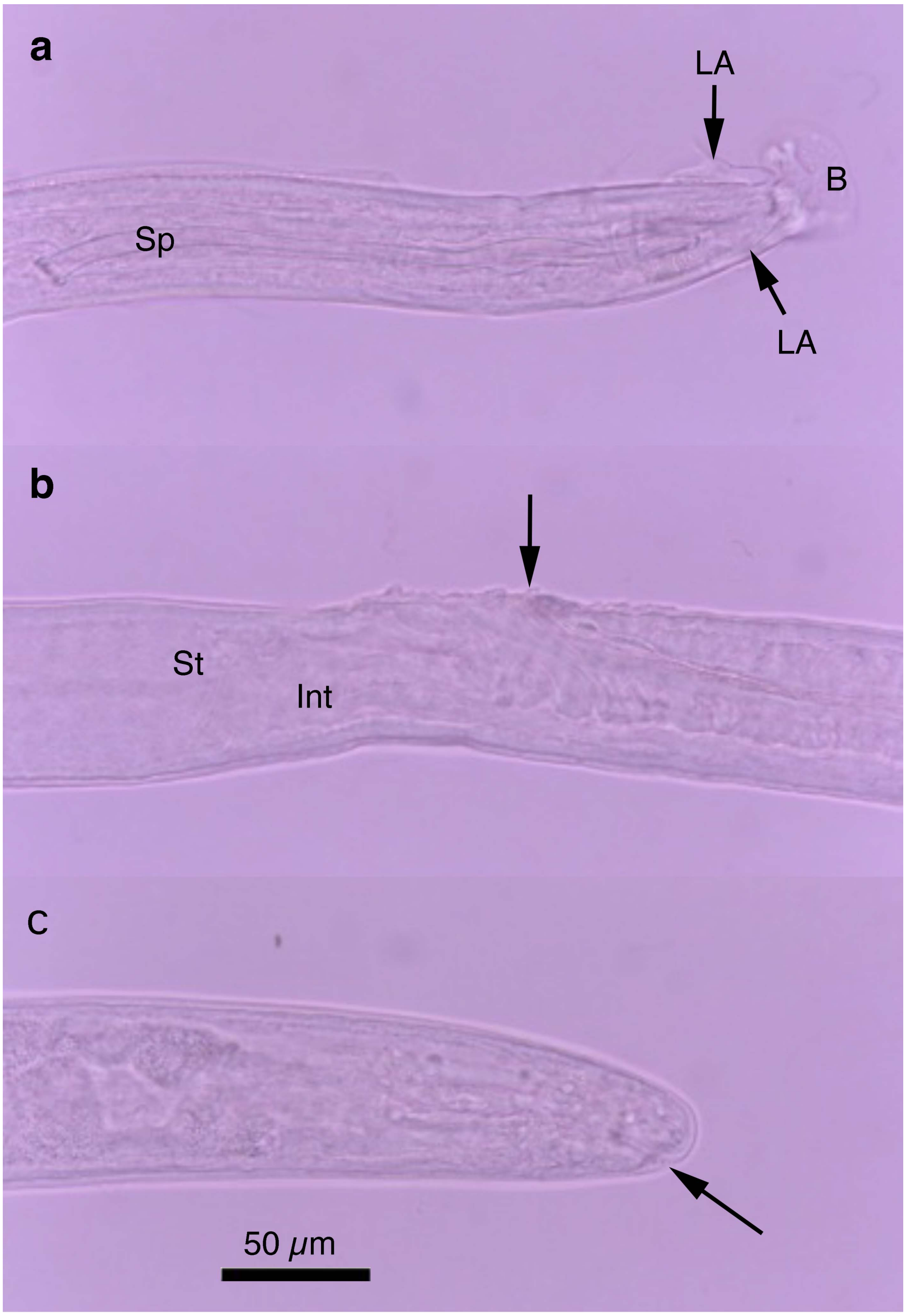

Suppl. Fig. S2. Morphology of *Aonchotheca bilobata*. (**a**) Caudal end of male worm with small triangular lateral alae (LA) and membranous bursa (B) supported by a pair of bifurcated ventrolateral projections. Spicule (Sp). (**b**) Roughened cuticular surface around the vulva (arrow). End of stichosome (St), and beginning of intestine (int). (**c**) Posterior end of female worm with subterminal anus (arrow). Photographs (**a**–**c**) at the same magnification, and scale bar is shown in (**c**).

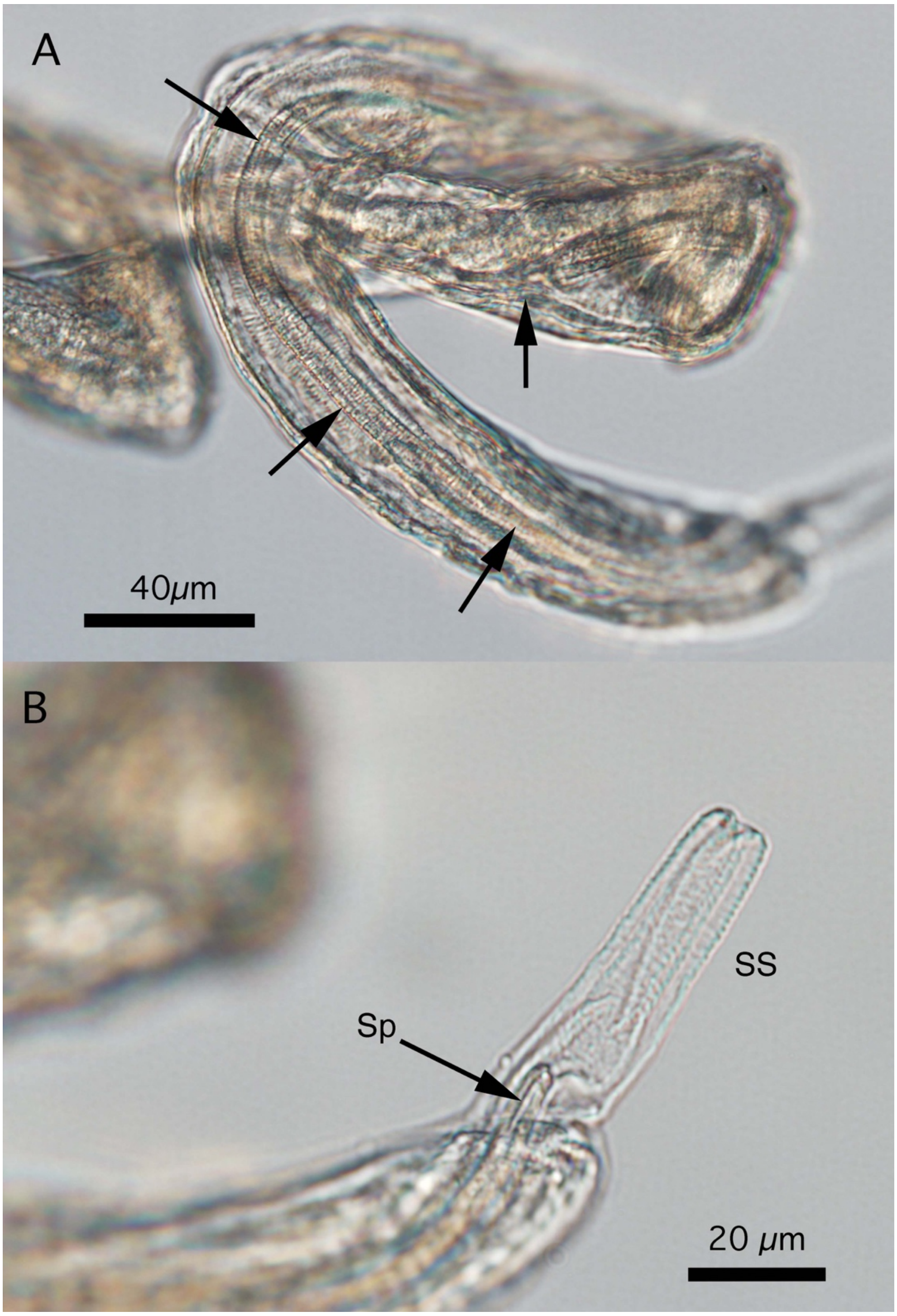

Suppl. Fig. S3. Morphology of *Liniscus himizu* male worm. (**a**) Caudal end of male worm. Spicule are indicated by arrows. (**b**) Extruded spicular sheath (SS) from cloaca, and a tip of spicule (Sp). Scale bar is shown for each photograph.

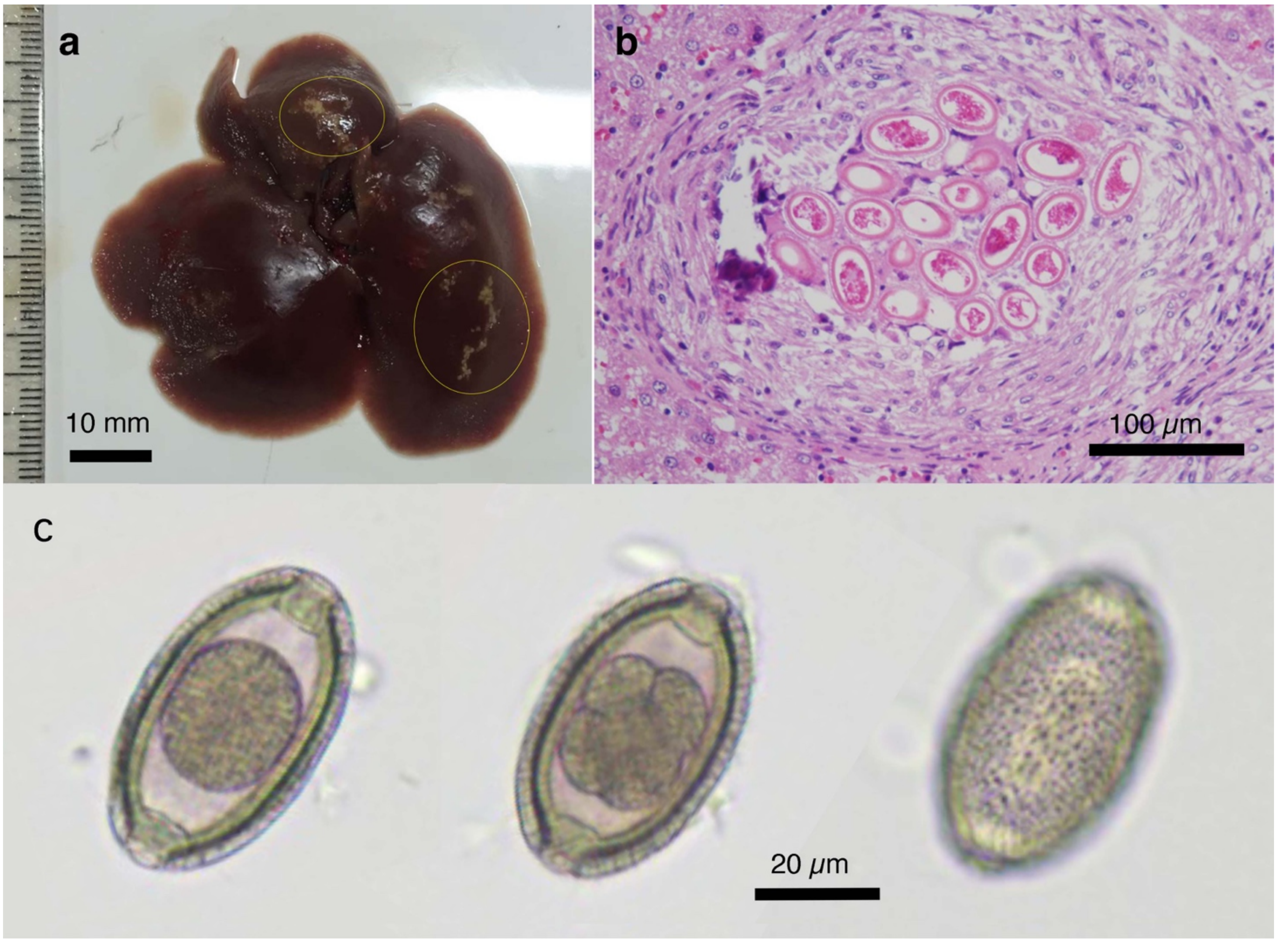

Suppl. Fig. S4. *Calodium hepaticum* infection in the liver of a brown rat. (**a**) Gross lesion of hepatic capillariasis with milky-white patchy lesions on the surface of liver (circled). (**b**) Histology of chronic granuloma around aggregated eggs. Hematoxylin-eosin stain. (**c**) Bi-operculated oval eggs of C. hepaticum with punctuated egg-shell surface (see the rightmost egg).

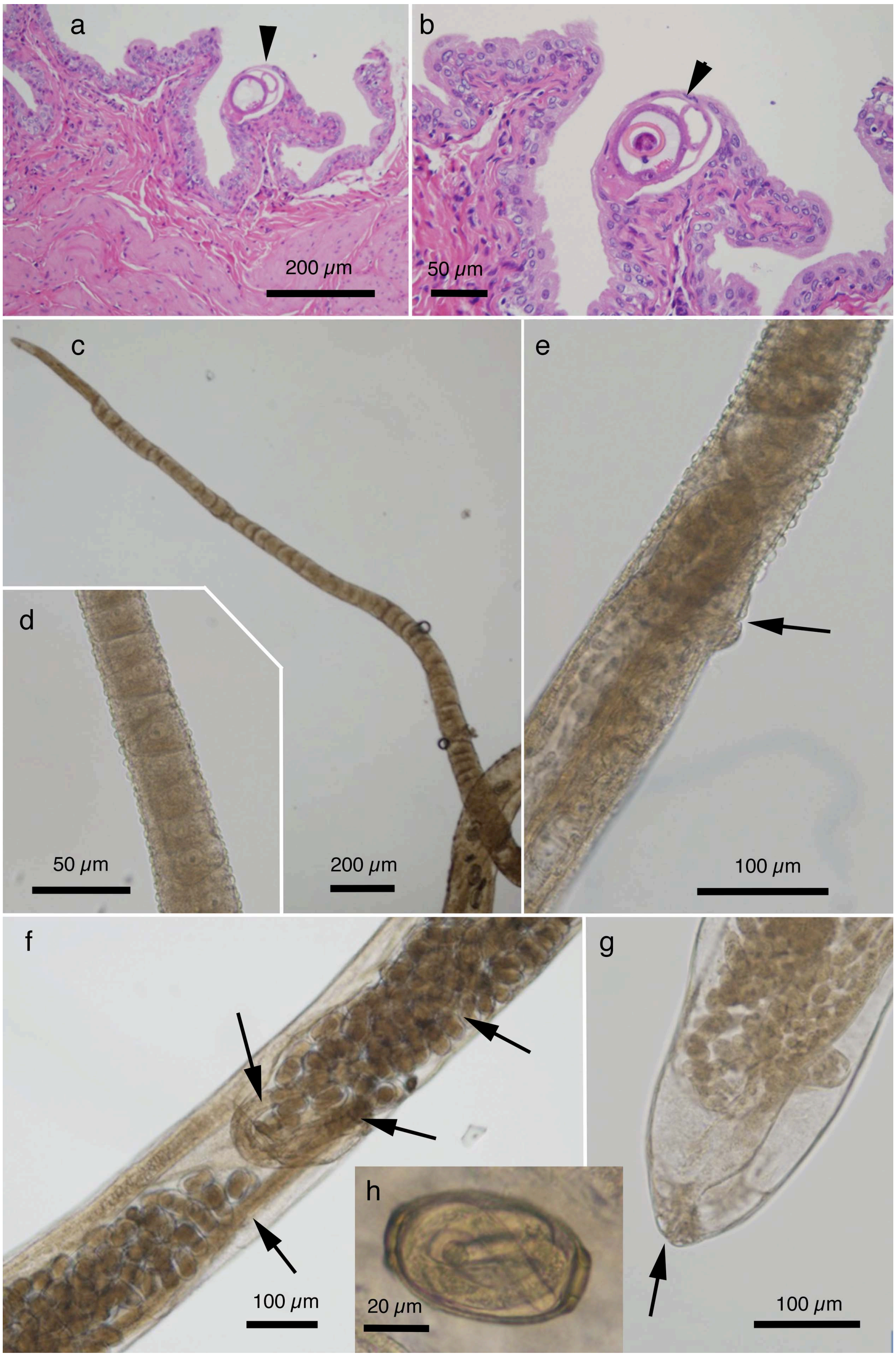

Suppl. Fig. S5. Morphology of a female *Trichosomoides crassicauda* worm collected from the urinary bladder of a brown rat. (**a, b**) Histology of the epithelium of urinary bladder parasitized with *T. crassicauda* (arrowheads). (**c**) Anterior part. (**d**) Stichocytes. (**e**) Vulva (arrow). (**f**) Uterus containing eggs and a male worm (arrows). (**g**) Caudal end of female worm with teminal anus (arrow). (**h**) larvated eggs.

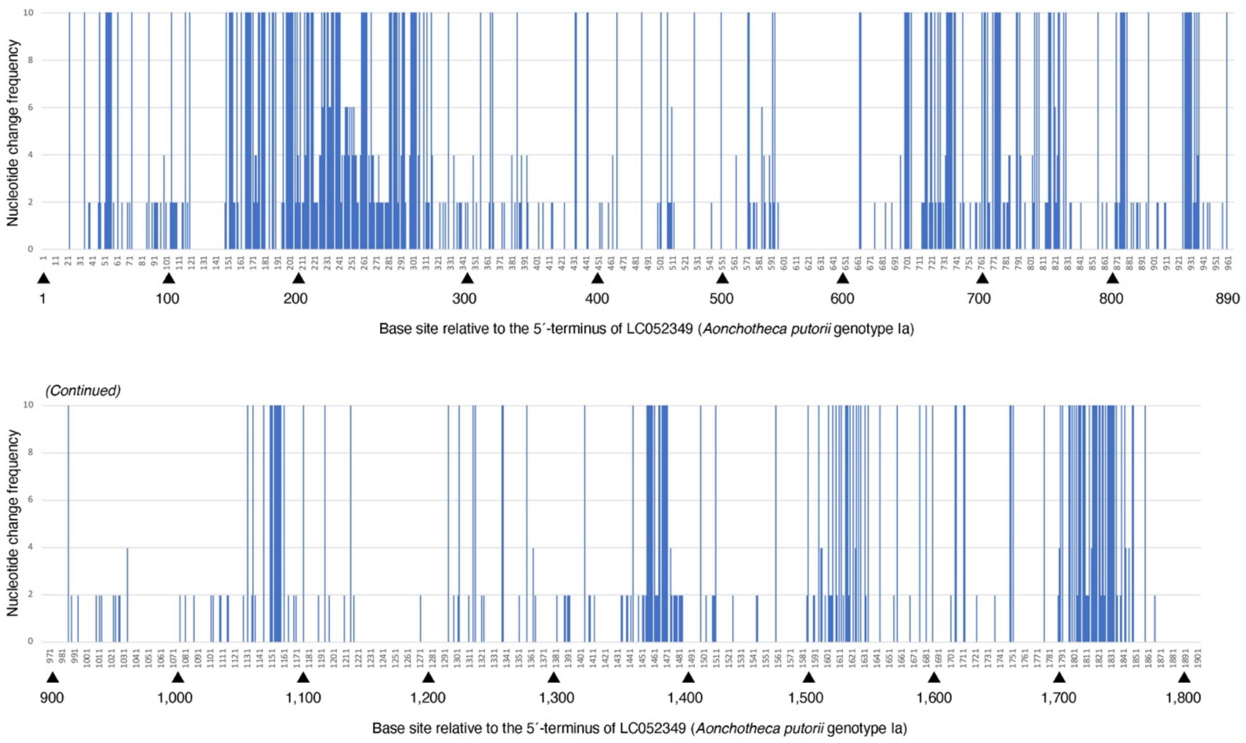

Suppl. Fig. S6. Frequence of nucleotide changes (substitutions and indels) over the capillariid SSU rDNA sequence. Base location is expressed using a SSU rDNA sequence of *A. putorii* (DDBJ/EMBL/GenBank accession no. LC052349). Interspecific and intraspecific nucleotide changes were compared using 88 isolates (classified into 28 species of 9 genera) as shown below. Intergeneric and interspecific variation are ranked as 10 (maximum), nucleotide changes in any one sequence as two, nucleotide changes by less than ten sequences as four, and nucleotide changes by ≥10 but less than 20 sequences as six, and those by more than 20 or more as eight. Used sequences for the analysis were as follows: *Aonchotheca* (*A. bilobate* [LC052379]; *A. bursata* [LC425006]; *A. paranalis* [MF621021]; *A .putorii* [LC052349–LCLC052364, OP028951]; *A. riukiuensis* [C052377, LC052378]; *A. suis* [LC052375, LC052376]; and *A._suzukii* n. sp. [LC052366–LC052374]), *Baruscapillaria* (*B. obsignata* [LC052336, LC425002–LC425005]), *Capillaria* (*C. anatis* [LC052334, LC052335, LC425001]; *C. madseni* [LC052344–LC052346, LC052348]; *C. phasianina* [LC777440]; *C. pudendotecta* [LC052339–LC052343]; and *C. spinulosa* [LC424999, LC425000]), *Echinocoleus* (*E. yokoyamae* n. sp. [LC052380, LC052381]), *Eucoleus* (*E. aerophilus* [MF599385, MW709573, LC052385]; *E. contortus* [LC424996]; *E. dispar* [EU004821]; *E. kaneshiroi* n. sp. [LC052382, LC052383]; *E. perforans* [LC424997, LC424998, LC777439]; *Eucoleus* sp. cat-2018 [MW709574]; *Eucoleus* sp. K33-S [PP951251]; and *Eucoleus* sp. Toda-2010c [LC052384]), *Pseudocapillaria* (*P._tomentosa* [KU987805]), *Calodium* (*C. hepaticum* [LC425008]), *Pearsonema* (*P. feliscati* [LC052388, LC052389, LC858137–LC858139, LC850894]; *P. iharai* n. sp. [LC052386, LC850896]; *P. neoplica* n. sp. [LC052390, LC858133–LC858136]; *P. plica* [MF621034]; *P. toriii* n. sp. [LC052387]), and *Liniscus* (*L. himizu* [LC850895]).
